# Electron efficiency measurements with the ATLAS detector using 2012 LHC proton–proton collision data

**DOI:** 10.1140/epjc/s10052-017-4756-2

**Published:** 2017-03-27

**Authors:** M. Aaboud, G. Aad, B. Abbott, J. Abdallah, O. Abdinov, B. Abeloos, O. S. AbouZeid, N. L. Abraham, H. Abramowicz, H. Abreu, R. Abreu, Y. Abulaiti, B. S. Acharya, S. Adachi, L. Adamczyk, D. L. Adams, J. Adelman, S. Adomeit, T. Adye, A. A. Affolder, T. Agatonovic-Jovin, J. A. Aguilar-Saavedra, S. P. Ahlen, F. Ahmadov, G. Aielli, H. Akerstedt, T. P. A. Åkesson, A. V. Akimov, G. L. Alberghi, J. Albert, S. Albrand, M. J. Alconada Verzini, M. Aleksa, I. N. Aleksandrov, C. Alexa, G. Alexander, T. Alexopoulos, M. Alhroob, B. Ali, M. Aliev, G. Alimonti, J. Alison, S. P. Alkire, B. M. M. Allbrooke, B. W. Allen, P. P. Allport, A. Aloisio, A. Alonso, F. Alonso, C. Alpigiani, A. A. Alshehri, M. Alstaty, B. Alvarez Gonzalez, D. Álvarez Piqueras, M. G. Alviggi, B. T. Amadio, Y. Amaral Coutinho, C. Amelung, D. Amidei, S. P. Amor Dos Santos, A. Amorim, S. Amoroso, G. Amundsen, C. Anastopoulos, L. S. Ancu, N. Andari, T. Andeen, C. F. Anders, J. K. Anders, K. J. Anderson, A. Andreazza, V. Andrei, S. Angelidakis, I. Angelozzi, A. Angerami, F. Anghinolfi, A. V. Anisenkov, N. Anjos, A. Annovi, C. Antel, M. Antonelli, A. Antonov, D. J. Antrim, F. Anulli, M. Aoki, L. Aperio Bella, G. Arabidze, Y. Arai, J. P. Araque, A. T. H. Arce, F. A. Arduh, J.-F. Arguin, S. Argyropoulos, M. Arik, A. J. Armbruster, L. J. Armitage, O. Arnaez, H. Arnold, M. Arratia, O. Arslan, A. Artamonov, G. Artoni, S. Artz, S. Asai, N. Asbah, A. Ashkenazi, B. Åsman, L. Asquith, K. Assamagan, R. Astalos, M. Atkinson, N. B. Atlay, K. Augsten, G. Avolio, B. Axen, M. K. Ayoub, G. Azuelos, M. A. Baak, A. E. Baas, M. J. Baca, H. Bachacou, K. Bachas, M. Backes, M. Backhaus, P. Bagiacchi, P. Bagnaia, Y. Bai, J. T. Baines, M. Bajic, O. K. Baker, E. M. Baldin, P. Balek, T. Balestri, F. Balli, W. K. Balunas, E. Banas, Sw. Banerjee, A. A. E. Bannoura, L. Barak, E. L. Barberio, D. Barberis, M. Barbero, T. Barillari, M.-S. Barisits, T. Barklow, N. Barlow, S. L. Barnes, B. M. Barnett, R. M. Barnett, Z. Barnovska-Blenessy, A. Baroncelli, G. Barone, A. J. Barr, L. Barranco Navarro, F. Barreiro, J. Barreiro Guimarães da Costa, R. Bartoldus, A. E. Barton, P. Bartos, A. Basalaev, A. Bassalat, R. L. Bates, S. J. Batista, J. R. Batley, M. Battaglia, M. Bauce, F. Bauer, H. S. Bawa, J. B. Beacham, M. D. Beattie, T. Beau, P. H. Beauchemin, P. Bechtle, H. P. Beck, K. Becker, M. Becker, M. Beckingham, C. Becot, A. J. Beddall, A. Beddall, V. A. Bednyakov, M. Bedognetti, C. P. Bee, L. J. Beemster, T. A. Beermann, M. Begel, J. K. Behr, A. S. Bell, G. Bella, L. Bellagamba, A. Bellerive, M. Bellomo, K. Belotskiy, O. Beltramello, N. L. Belyaev, O. Benary, D. Benchekroun, M. Bender, K. Bendtz, N. Benekos, Y. Benhammou, E. Benhar Noccioli, J. Benitez, D. P. Benjamin, J. R. Bensinger, S. Bentvelsen, L. Beresford, M. Beretta, D. Berge, E. Bergeaas Kuutmann, N. Berger, J. Beringer, S. Berlendis, N. R. Bernard, C. Bernius, F. U. Bernlochner, T. Berry, P. Berta, C. Bertella, G. Bertoli, F. Bertolucci, I. A. Bertram, C. Bertsche, D. Bertsche, G. J. Besjes, O. Bessidskaia Bylund, M. Bessner, N. Besson, C. Betancourt, A. Bethani, S. Bethke, A. J. Bevan, R. M. Bianchi, M. Bianco, O. Biebel, D. Biedermann, R. Bielski, N. V. Biesuz, M. Biglietti, J. Bilbao De Mendizabal, T. R. V. Billoud, H. Bilokon, M. Bindi, A. Bingul, C. Bini, S. Biondi, T. Bisanz, D. M. Bjergaard, C. W. Black, J. E. Black, K. M. Black, D. Blackburn, R. E. Blair, T. Blazek, I. Bloch, C. Blocker, A. Blue, W. Blum, U. Blumenschein, S. Blunier, G. J. Bobbink, V. S. Bobrovnikov, S. S. Bocchetta, A. Bocci, C. Bock, M. Boehler, D. Boerner, J. A. Bogaerts, D. Bogavac, A. G. Bogdanchikov, C. Bohm, V. Boisvert, P. Bokan, T. Bold, A. S. Boldyrev, M. Bomben, M. Bona, M. Boonekamp, A. Borisov, G. Borissov, J. Bortfeldt, D. Bortoletto, V. Bortolotto, K. Bos, D. Boscherini, M. Bosman, J. D. Bossio Sola, J. Boudreau, J. Bouffard, E. V. Bouhova-Thacker, D. Boumediene, C. Bourdarios, S. K. Boutle, A. Boveia, J. Boyd, I. R. Boyko, J. Bracinik, A. Brandt, G. Brandt, O. Brandt, U. Bratzler, B. Brau, J. E. Brau, W. D. Breaden Madden, K. Brendlinger, A. J. Brennan, L. Brenner, R. Brenner, S. Bressler, T. M. Bristow, D. Britton, D. Britzger, F. M. Brochu, I. Brock, R. Brock, G. Brooijmans, T. Brooks, W. K. Brooks, J. Brosamer, E. Brost, J. H. Broughton, P. A. Bruckman de Renstrom, D. Bruncko, R. Bruneliere, A. Bruni, G. Bruni, L. S. Bruni, B. H. Brunt, M. Bruschi, N. Bruscino, P. Bryant, L. Bryngemark, T. Buanes, Q. Buat, P. Buchholz, A. G. Buckley, I. A. Budagov, F. Buehrer, M. K. Bugge, O. Bulekov, D. Bullock, H. Burckhart, S. Burdin, C. D. Burgard, A. M. Burger, B. Burghgrave, K. Burka, S. Burke, I. Burmeister, J. T. P. Burr, E. Busato, D. Büscher, V. Büscher, P. Bussey, J. M. Butler, C. M. Buttar, J. M. Butterworth, P. Butti, W. Buttinger, A. Buzatu, A. R. Buzykaev, S. Cabrera Urbán, D. Caforio, V. M. Cairo, O. Cakir, N. Calace, P. Calafiura, A. Calandri, G. Calderini, P. Calfayan, G. Callea, L. P. Caloba, S. Calvente Lopez, D. Calvet, S. Calvet, T. P. Calvet, R. Camacho Toro, S. Camarda, P. Camarri, D. Cameron, R. Caminal Armadans, C. Camincher, S. Campana, M. Campanelli, A. Camplani, A. Campoverde, V. Canale, A. Canepa, M. Cano Bret, J. Cantero, T. Cao, M. D. M. Capeans Garrido, I. Caprini, M. Caprini, M. Capua, R. M. Carbone, R. Cardarelli, F. Cardillo, I. Carli, T. Carli, G. Carlino, B. T. Carlson, L. Carminati, R. M. D. Carney, S. Caron, E. Carquin, G. D. Carrillo-Montoya, J. R. Carter, J. Carvalho, D. Casadei, M. P. Casado, M. Casolino, D. W. Casper, E. Castaneda-Miranda, R. Castelijn, A. Castelli, V. Castillo Gimenez, N. F. Castro, A. Catinaccio, J. R. Catmore, A. Cattai, J. Caudron, V. Cavaliere, E. Cavallaro, D. Cavalli, M. Cavalli-Sforza, V. Cavasinni, F. Ceradini, L. Cerda Alberich, A. S. Cerqueira, A. Cerri, L. Cerrito, F. Cerutti, A. Cervelli, S. A. Cetin, A. Chafaq, D. Chakraborty, S. K. Chan, Y. L. Chan, P. Chang, J. D. Chapman, D. G. Charlton, A. Chatterjee, C. C. Chau, C. A. Chavez Barajas, S. Che, S. Cheatham, A. Chegwidden, S. Chekanov, S. V. Chekulaev, G. A. Chelkov, M. A. Chelstowska, C. Chen, H. Chen, S. Chen, S. Chen, X. Chen, Y. Chen, H. C. Cheng, H. J. Cheng, Y. Cheng, A. Cheplakov, E. Cheremushkina, R. Cherkaoui El Moursli, V. Chernyatin, E. Cheu, L. Chevalier, V. Chiarella, G. Chiarelli, G. Chiodini, A. S. Chisholm, A. Chitan, M. V. Chizhov, K. Choi, A. R. Chomont, S. Chouridou, B. K. B. Chow, V. Christodoulou, D. Chromek-Burckhart, J. Chudoba, A. J. Chuinard, J. J. Chwastowski, L. Chytka, G. Ciapetti, A. K. Ciftci, D. Cinca, V. Cindro, I. A. Cioara, C. Ciocca, A. Ciocio, F. Cirotto, Z. H. Citron, M. Citterio, M. Ciubancan, A. Clark, B. L. Clark, M. R. Clark, P. J. Clark, R. N. Clarke, C. Clement, Y. Coadou, M. Cobal, A. Coccaro, J. Cochran, L. Colasurdo, B. Cole, A. P. Colijn, J. Collot, T. Colombo, P. Conde Muiño, E. Coniavitis, S. H. Connell, I. A. Connelly, V. Consorti, S. Constantinescu, G. Conti, F. Conventi, M. Cooke, B. D. Cooper, A. M. Cooper-Sarkar, F. Cormier, K. J. R. Cormier, T. Cornelissen, M. Corradi, F. Corriveau, A. Cortes-Gonzalez, G. Cortiana, G. Costa, M. J. Costa, D. Costanzo, G. Cottin, G. Cowan, B. E. Cox, K. Cranmer, S. J. Crawley, G. Cree, S. Crépé-Renaudin, F. Crescioli, W. A. Cribbs, M. Crispin Ortuzar, M. Cristinziani, V. Croft, G. Crosetti, A. Cueto, T. Cuhadar Donszelmann, J. Cummings, M. Curatolo, J. Cúth, H. Czirr, P. Czodrowski, G. D’amen, S. D’Auria, M. D’Onofrio, M. J. Da Cunha Sargedas De Sousa, C. Da Via, W. Dabrowski, T. Dado, T. Dai, O. Dale, F. Dallaire, C. Dallapiccola, M. Dam, J. R. Dandoy, N. P. Dang, A. C. Daniells, N. S. Dann, M. Danninger, M. Dano Hoffmann, V. Dao, G. Darbo, S. Darmora, J. Dassoulas, A. Dattagupta, W. Davey, C. David, T. Davidek, M. Davies, P. Davison, E. Dawe, I. Dawson, K. De, R. de Asmundis, A. De Benedetti, S. De Castro, S. De Cecco, N. De Groot, P. de Jong, H. De la Torre, F. De Lorenzi, A. De Maria, D. De Pedis, A. De Salvo, U. De Sanctis, A. De Santo, J. B. De Vivie De Regie, W. J. Dearnaley, R. Debbe, C. Debenedetti, D. V. Dedovich, N. Dehghanian, I. Deigaard, M. Del Gaudio, J. Del Peso, T. Del Prete, D. Delgove, F. Deliot, C. M. Delitzsch, A. Dell’Acqua, L. Dell’Asta, M. Dell’Orso, M. Della Pietra, D. della Volpe, M. Delmastro, P. A. Delsart, D. A. DeMarco, S. Demers, M. Demichev, A. Demilly, S. P. Denisov, D. Denysiuk, D. Derendarz, J. E. Derkaoui, F. Derue, P. Dervan, K. Desch, C. Deterre, K. Dette, P. O. Deviveiros, A. Dewhurst, S. Dhaliwal, A. Di Ciaccio, L. Di Ciaccio, W. K. Di Clemente, C. Di Donato, A. Di Girolamo, B. Di Girolamo, B. Di Micco, R. Di Nardo, K. F. Di Petrillo, A. Di Simone, R. Di Sipio, D. Di Valentino, C. Diaconu, M. Diamond, F. A. Dias, M. A. Diaz, E. B. Diehl, J. Dietrich, S. Díez Cornell, A. Dimitrievska, J. Dingfelder, P. Dita, S. Dita, F. Dittus, F. Djama, T. Djobava, J. I. Djuvsland, M. A. B. do Vale, D. Dobos, M. Dobre, C. Doglioni, J. Dolejsi, Z. Dolezal, M. Donadelli, S. Donati, P. Dondero, J. Donini, J. Dopke, A. Doria, M. T. Dova, A. T. Doyle, E. Drechsler, M. Dris, Y. Du, J. Duarte-Campderros, E. Duchovni, G. Duckeck, O. A. Ducu, D. Duda, A. Dudarev, A. Chr. Dudder, E. M. Duffield, L. Duflot, M. Dührssen, M. Dumancic, A. K. Duncan, M. Dunford, H. Duran Yildiz, M. Düren, A. Durglishvili, D. Duschinger, B. Dutta, M. Dyndal, C. Eckardt, K. M. Ecker, R. C. Edgar, N. C. Edwards, T. Eifert, G. Eigen, K. Einsweiler, T. Ekelof, M. El Kacimi, V. Ellajosyula, M. Ellert, S. Elles, F. Ellinghaus, A. A. Elliot, N. Ellis, J. Elmsheuser, M. Elsing, D. Emeliyanov, Y. Enari, O. C. Endner, J. S. Ennis, J. Erdmann, A. Ereditato, G. Ernis, J. Ernst, M. Ernst, S. Errede, E. Ertel, M. Escalier, H. Esch, C. Escobar, B. Esposito, A. I. Etienvre, E. Etzion, H. Evans, A. Ezhilov, F. Fabbri, L. Fabbri, G. Facini, R. M. Fakhrutdinov, S. Falciano, R. J. Falla, J. Faltova, Y. Fang, M. Fanti, A. Farbin, A. Farilla, C. Farina, E. M. Farina, T. Farooque, S. Farrell, S. M. Farrington, P. Farthouat, F. Fassi, P. Fassnacht, D. Fassouliotis, M. Faucci Giannelli, A. Favareto, W. J. Fawcett, L. Fayard, O. L. Fedin, W. Fedorko, S. Feigl, L. Feligioni, C. Feng, E. J. Feng, H. Feng, A. B. Fenyuk, L. Feremenga, P. Fernandez Martinez, S. Fernandez Perez, J. Ferrando, A. Ferrari, P. Ferrari, R. Ferrari, D. E. Ferreira de Lima, A. Ferrer, D. Ferrere, C. Ferretti, F. Fiedler, A. Filipčič, M. Filipuzzi, F. Filthaut, M. Fincke-Keeler, K. D. Finelli, M. C. N. Fiolhais, L. Fiorini, A. Fischer, C. Fischer, J. Fischer, W. C. Fisher, N. Flaschel, I. Fleck, P. Fleischmann, G. T. Fletcher, R. R. M. Fletcher, T. Flick, B. M. Flierl, L. R. Flores Castillo, M. J. Flowerdew, G. T. Forcolin, A. Formica, A. Forti, A. G. Foster, D. Fournier, H. Fox, S. Fracchia, P. Francavilla, M. Franchini, D. Francis, L. Franconi, M. Franklin, M. Frate, M. Fraternali, D. Freeborn, S. M. Fressard-Batraneanu, F. Friedrich, D. Froidevaux, J. A. Frost, C. Fukunaga, E. Fullana Torregrosa, T. Fusayasu, J. Fuster, C. Gabaldon, O. Gabizon, A. Gabrielli, A. Gabrielli, G. P. Gach, S. Gadatsch, G. Gagliardi, L. G. Gagnon, P. Gagnon, C. Galea, B. Galhardo, E. J. Gallas, B. J. Gallop, P. Gallus, G. Galster, K. K. Gan, S. Ganguly, J. Gao, Y. Gao, Y. S. Gao, F. M. Garay Walls, C. García, J. E. García Navarro, M. Garcia-Sciveres, R. W. Gardner, N. Garelli, V. Garonne, A. Gascon Bravo, K. Gasnikova, C. Gatti, A. Gaudiello, G. Gaudio, L. Gauthier, I. L. Gavrilenko, C. Gay, G. Gaycken, E. N. Gazis, Z. Gecse, C. N. P. Gee, Ch. Geich-Gimbel, M. Geisen, M. P. Geisler, K. Gellerstedt, C. Gemme, M. H. Genest, C. Geng, S. Gentile, C. Gentsos, S. George, D. Gerbaudo, A. Gershon, S. Ghasemi, M. Ghneimat, B. Giacobbe, S. Giagu, P. Giannetti, S. M. Gibson, M. Gignac, M. Gilchriese, T. P. S. Gillam, D. Gillberg, G. Gilles, D. M. Gingrich, N. Giokaris, M. P. Giordani, F. M. Giorgi, P. F. Giraud, P. Giromini, D. Giugni, F. Giuli, C. Giuliani, M. Giulini, B. K. Gjelsten, S. Gkaitatzis, I. Gkialas, E. L. Gkougkousis, L. K. Gladilin, C. Glasman, J. Glatzer, P. C. F. Glaysher, A. Glazov, M. Goblirsch-Kolb, J. Godlewski, S. Goldfarb, T. Golling, D. Golubkov, A. Gomes, R. Gonçalo, J. Goncalves Pinto Firmino Da Costa, G. Gonella, L. Gonella, A. Gongadze, S. González de la Hoz, S. Gonzalez-Sevilla, L. Goossens, P. A. Gorbounov, H. A. Gordon, I. Gorelov, B. Gorini, E. Gorini, A. Gorišek, A. T. Goshaw, C. Gössling, M. I. Gostkin, C. R. Goudet, D. Goujdami, A. G. Goussiou, N. Govender, E. Gozani, L. Graber, I. Grabowska-Bold, P. O. J. Gradin, P. Grafström, J. Gramling, E. Gramstad, S. Grancagnolo, V. Gratchev, P. M. Gravila, H. M. Gray, E. Graziani, Z. D. Greenwood, C. Grefe, K. Gregersen, I. M. Gregor, P. Grenier, K. Grevtsov, J. Griffiths, A. A. Grillo, K. Grimm, S. Grinstein, Ph. Gris, J. -F. Grivaz, S. Groh, E. Gross, J. Grosse-Knetter, G. C. Grossi, Z. J. Grout, L. Guan, W. Guan, J. Guenther, F. Guescini, D. Guest, O. Gueta, B. Gui, E. Guido, T. Guillemin, S. Guindon, U. Gul, C. Gumpert, J. Guo, W. Guo, Y. Guo, R. Gupta, S. Gupta, G. Gustavino, P. Gutierrez, N. G. Gutierrez Ortiz, C. Gutschow, C. Guyot, C. Gwenlan, C. B. Gwilliam, A. Haas, C. Haber, H. K. Hadavand, A. Hadef, S. Hageböck, M. Hagihara, H. Hakobyan, M. Haleem, J. Haley, G. Halladjian, G. D. Hallewell, K. Hamacher, P. Hamal, K. Hamano, A. Hamilton, G. N. Hamity, P. G. Hamnett, L. Han, S. Han, K. Hanagaki, K. Hanawa, M. Hance, B. Haney, P. Hanke, R. Hanna, J. B. Hansen, J. D. Hansen, M. C. Hansen, P. H. Hansen, K. Hara, A. S. Hard, T. Harenberg, F. Hariri, S. Harkusha, R. D. Harrington, P. F. Harrison, F. Hartjes, N. M. Hartmann, M. Hasegawa, Y. Hasegawa, A. Hasib, S. Hassani, S. Haug, R. Hauser, L. Hauswald, M. Havranek, C. M. Hawkes, R. J. Hawkings, D. Hayakawa, D. Hayden, C. P. Hays, J. M. Hays, H. S. Hayward, S. J. Haywood, S. J. Head, T. Heck, V. Hedberg, L. Heelan, S. Heim, T. Heim, B. Heinemann, J. J. Heinrich, L. Heinrich, C. Heinz, J. Hejbal, L. Helary, S. Hellman, C. Helsens, J. Henderson, R. C. W. Henderson, Y. Heng, S. Henkelmann, A. M. Henriques Correia, S. Henrot-Versille, G. H. Herbert, H. Herde, V. Herget, Y. Hernández Jiménez, G. Herten, R. Hertenberger, L. Hervas, G. G. Hesketh, N. P. Hessey, J. W. Hetherly, E. Higón-Rodriguez, E. Hill, J. C. Hill, K. H. Hiller, S. J. Hillier, I. Hinchliffe, E. Hines, M. Hirose, D. Hirschbuehl, O. Hladik, X. Hoad, J. Hobbs, N. Hod, M. C. Hodgkinson, P. Hodgson, A. Hoecker, M. R. Hoeferkamp, F. Hoenig, D. Hohn, T. R. Holmes, M. Homann, S. Honda, T. Honda, T. M. Hong, B. H. Hooberman, W. H. Hopkins, Y. Horii, A. J. Horton, J-Y. Hostachy, S. Hou, A. Hoummada, J. Howarth, J. Hoya, M. Hrabovsky, I. Hristova, J. Hrivnac, T. Hryn’ova, A. Hrynevich, P. J. Hsu, S. -C. Hsu, Q. Hu, S. Hu, Y. Huang, Z. Hubacek, F. Hubaut, F. Huegging, T. B. Huffman, E. W. Hughes, G. Hughes, M. Huhtinen, P. Huo, N. Huseynov, J. Huston, J. Huth, G. Iacobucci, G. Iakovidis, I. Ibragimov, L. Iconomidou-Fayard, E. Ideal, P. Iengo, O. Igonkina, T. Iizawa, Y. Ikegami, M. Ikeno, Y. Ilchenko, D. Iliadis, N. Ilic, G. Introzzi, P. Ioannou, M. Iodice, K. Iordanidou, V. Ippolito, N. Ishijima, M. Ishino, M. Ishitsuka, C. Issever, S. Istin, F. Ito, J. M. Iturbe Ponce, R. Iuppa, H. Iwasaki, J. M. Izen, V. Izzo, S. Jabbar, B. Jackson, P. Jackson, V. Jain, K. B. Jakobi, K. Jakobs, S. Jakobsen, T. Jakoubek, D. O. Jamin, D. K. Jana, R. Jansky, J. Janssen, M. Janus, P. A. Janus, G. Jarlskog, N. Javadov, T. Javůrek, M. Javurkova, F. Jeanneau, L. Jeanty, J. Jejelava, G. -Y. Jeng, P. Jenni, C. Jeske, S. Jézéquel, H. Ji, J. Jia, H. Jiang, Y. Jiang, Z. Jiang, S. Jiggins, J. Jimenez Pena, S. Jin, A. Jinaru, O. Jinnouchi, H. Jivan, P. Johansson, K. A. Johns, C. A. Johnson, W. J. Johnson, K. Jon-And, G. Jones, R. W. L. Jones, S. Jones, T. J. Jones, J. Jongmanns, P. M. Jorge, J. Jovicevic, X. Ju, A. Juste Rozas, M. K. Köhler, A. Kaczmarska, M. Kado, H. Kagan, M. Kagan, S. J. Kahn, T. Kaji, E. Kajomovitz, C. W. Kalderon, A. Kaluza, S. Kama, A. Kamenshchikov, N. Kanaya, S. Kaneti, L. Kanjir, V. A. Kantserov, J. Kanzaki, B. Kaplan, L. S. Kaplan, A. Kapliy, D. Kar, K. Karakostas, A. Karamaoun, N. Karastathis, M. J. Kareem, E. Karentzos, M. Karnevskiy, S. N. Karpov, Z. M. Karpova, K. Karthik, V. Kartvelishvili, A. N. Karyukhin, K. Kasahara, L. Kashif, R. D. Kass, A. Kastanas, Y. Kataoka, C. Kato, A. Katre, J. Katzy, K. Kawade, K. Kawagoe, T. Kawamoto, G. Kawamura, V. F. Kazanin, R. Keeler, R. Kehoe, J. S. Keller, J. J. Kempster, H. Keoshkerian, O. Kepka, B. P. Kerševan, S. Kersten, R. A. Keyes, M. Khader, F. Khalil-zada, A. Khanov, A. G. Kharlamov, T. Kharlamova, T. J. Khoo, V. Khovanskiy, E. Khramov, J. Khubua, S. Kido, C. R. Kilby, H. Y. Kim, S. H. Kim, Y. K. Kim, N. Kimura, O. M. Kind, B. T. King, M. King, J. Kirk, A. E. Kiryunin, T. Kishimoto, D. Kisielewska, F. Kiss, K. Kiuchi, O. Kivernyk, E. Kladiva, M. H. Klein, M. Klein, U. Klein, K. Kleinknecht, P. Klimek, A. Klimentov, R. Klingenberg, T. Klioutchnikova, E. -E. Kluge, P. Kluit, S. Kluth, J. Knapik, E. Kneringer, E. B. F. G. Knoops, A. Knue, A. Kobayashi, D. Kobayashi, T. Kobayashi, M. Kobel, M. Kocian, P. Kodys, T. Koffas, E. Koffeman, N. M. Köhler, T. Koi, H. Kolanoski, M. Kolb, I. Koletsou, A. A. Komar, Y. Komori, T. Kondo, N. Kondrashova, K. Köneke, A. C. König, T. Kono, R. Konoplich, N. Konstantinidis, R. Kopeliansky, S. Koperny, A. K. Kopp, K. Korcyl, K. Kordas, A. Korn, A. A. Korol, I. Korolkov, E. V. Korolkova, O. Kortner, S. Kortner, T. Kosek, V. V. Kostyukhin, A. Kotwal, A. Koulouris, A. Kourkoumeli-Charalampidi, C. Kourkoumelis, V. Kouskoura, A. B. Kowalewska, R. Kowalewski, T. Z. Kowalski, C. Kozakai, W. Kozanecki, A. S. Kozhin, V. A. Kramarenko, G. Kramberger, D. Krasnopevtsev, M. W. Krasny, A. Krasznahorkay, A. Kravchenko, M. Kretz, J. Kretzschmar, K. Kreutzfeldt, P. Krieger, K. Krizka, K. Kroeninger, H. Kroha, J. Kroll, J. Kroseberg, J. Krstic, U. Kruchonak, H. Krüger, N. Krumnack, M. C. Kruse, M. Kruskal, T. Kubota, H. Kucuk, S. Kuday, J. T. Kuechler, S. Kuehn, A. Kugel, F. Kuger, T. Kuhl, V. Kukhtin, R. Kukla, Y. Kulchitsky, S. Kuleshov, M. Kuna, T. Kunigo, A. Kupco, O. Kuprash, H. Kurashige, L. L. Kurchaninov, Y. A. Kurochkin, M. G. Kurth, V. Kus, E. S. Kuwertz, M. Kuze, J. Kvita, T. Kwan, D. Kyriazopoulos, A. La Rosa, J. L. La Rosa Navarro, L. La Rotonda, C. Lacasta, F. Lacava, J. Lacey, H. Lacker, D. Lacour, E. Ladygin, R. Lafaye, B. Laforge, T. Lagouri, S. Lai, S. Lammers, W. Lampl, E. Lançon, U. Landgraf, M. P. J. Landon, M. C. Lanfermann, V. S. Lang, J. C. Lange, A. J. Lankford, F. Lanni, K. Lantzsch, A. Lanza, S. Laplace, C. Lapoire, J. F. Laporte, T. Lari, F. Lasagni Manghi, M. Lassnig, P. Laurelli, W. Lavrijsen, A. T. Law, P. Laycock, T. Lazovich, M. Lazzaroni, B. Le, O. Le Dortz, E. Le Guirriec, E. P. Le Quilleuc, M. LeBlanc, T. LeCompte, F. Ledroit-Guillon, C. A. Lee, S. C. Lee, L. Lee, B. Lefebvre, G. Lefebvre, M. Lefebvre, F. Legger, C. Leggett, A. Lehan, G. Lehmann Miotto, X. Lei, W. A. Leight, A. G. Leister, M. A. L. Leite, R. Leitner, D. Lellouch, B. Lemmer, K. J. C. Leney, T. Lenz, B. Lenzi, R. Leone, S. Leone, C. Leonidopoulos, S. Leontsinis, G. Lerner, C. Leroy, A. A. J. Lesage, C. G. Lester, C. M. Lester, M. Levchenko, J. Levêque, D. Levin, L. J. Levinson, M. Levy, D. Lewis, M. Leyton, B. Li, C. Li, H. Li, L. Li, L. Li, Q. Li, S. Li, X. Li, Y. Li, Z. Liang, B. Liberti, A. Liblong, P. Lichard, K. Lie, J. Liebal, W. Liebig, A. Limosani, S. C. Lin, T. H. Lin, B. E. Lindquist, A. E. Lionti, E. Lipeles, A. Lipniacka, M. Lisovyi, T. M. Liss, A. Lister, A. M. Litke, B. Liu, D. Liu, H. Liu, H. Liu, J. Liu, J. B. Liu, K. Liu, L. Liu, M. Liu, Y. L. Liu, Y. Liu, M. Livan, A. Lleres, J. Llorente Merino, S. L. Lloyd, F. Lo Sterzo, E. M. Lobodzinska, P. Loch, F. K. Loebinger, K. M. Loew, A. Loginov, T. Lohse, K. Lohwasser, M. Lokajicek, B. A. Long, J. D. Long, R. E. Long, L. Longo, K. A. Looper, J. A. Lopez, D. Lopez Mateos, B. Lopez Paredes, I. Lopez Paz, A. Lopez Solis, J. Lorenz, N. Lorenzo Martinez, M. Losada, P. J. Lösel, X. Lou, A. Lounis, J. Love, P. A. Love, H. Lu, N. Lu, H. J. Lubatti, C. Luci, A. Lucotte, C. Luedtke, F. Luehring, W. Lukas, L. Luminari, O. Lundberg, B. Lund-Jensen, P. M. Luzi, D. Lynn, R. Lysak, E. Lytken, V. Lyubushkin, H. Ma, L. L. Ma, Y. Ma, G. Maccarrone, A. Macchiolo, C. M. Macdonald, B. Maček, J. Machado Miguens, D. Madaffari, R. Madar, H. J. Maddocks, W. F. Mader, A. Madsen, J. Maeda, S. Maeland, T. Maeno, A. Maevskiy, E. Magradze, J. Mahlstedt, C. Maiani, C. Maidantchik, A. A. Maier, T. Maier, A. Maio, S. Majewski, Y. Makida, N. Makovec, B. Malaescu, Pa. Malecki, V. P. Maleev, F. Malek, U. Mallik, D. Malon, C. Malone, S. Maltezos, S. Malyukov, J. Mamuzic, G. Mancini, L. Mandelli, I. Mandić, J. Maneira, L. Manhaes de Andrade Filho, J. Manjarres Ramos, A. Mann, A. Manousos, B. Mansoulie, J. D. Mansour, R. Mantifel, M. Mantoani, S. Manzoni, L. Mapelli, G. Marceca, L. March, G. Marchiori, M. Marcisovsky, M. Marjanovic, D. E. Marley, F. Marroquim, S. P. Marsden, Z. Marshall, S. Marti-Garcia, B. Martin, T. A. Martin, V. J. Martin, B. Martin dit Latour, M. Martinez, V. I. Martinez Outschoorn, S. Martin-Haugh, V. S. Martoiu, A. C. Martyniuk, A. Marzin, L. Masetti, T. Mashimo, R. Mashinistov, J. Masik, A. L. Maslennikov, I. Massa, L. Massa, P. Mastrandrea, A. Mastroberardino, T. Masubuchi, P. Mättig, J. Mattmann, J. Maurer, S. J. Maxfield, D. A. Maximov, R. Mazini, I. Maznas, S. M. Mazza, N. C. Mc Fadden, G. Mc Goldrick, S. P. Mc Kee, A. McCarn, R. L. McCarthy, T. G. McCarthy, L. I. McClymont, E. F. McDonald, J. A. Mcfayden, G. Mchedlidze, S. J. McMahon, R. A. McPherson, M. Medinnis, S. Meehan, S. Mehlhase, A. Mehta, K. Meier, C. Meineck, B. Meirose, D. Melini, B. R. Mellado Garcia, M. Melo, F. Meloni, S. B. Menary, L. Meng, X. T. Meng, A. Mengarelli, S. Menke, E. Meoni, S. Mergelmeyer, P. Mermod, L. Merola, C. Meroni, F. S. Merritt, A. Messina, J. Metcalfe, A. S. Mete, C. Meyer, C. Meyer, J-P. Meyer, J. Meyer, H. Meyer Zu Theenhausen, F. Miano, R. P. Middleton, S. Miglioranzi, L. Mijović, G. Mikenberg, M. Mikestikova, M. Mikuž, M. Milesi, A. Milic, D. W. Miller, C. Mills, A. Milov, D. A. Milstead, A. A. Minaenko, Y. Minami, I. A. Minashvili, A. I. Mincer, B. Mindur, M. Mineev, Y. Minegishi, Y. Ming, L. M. Mir, K. P. Mistry, T. Mitani, J. Mitrevski, V. A. Mitsou, A. Miucci, P. S. Miyagawa, A. Mizukami, J. U. Mjörnmark, M. Mlynarikova, T. Moa, K. Mochizuki, P. Mogg, S. Mohapatra, S. Molander, R. Moles-Valls, R. Monden, M. C. Mondragon, K. Mönig, J. Monk, E. Monnier, A. Montalbano, J. Montejo Berlingen, F. Monticelli, S. Monzani, R. W. Moore, N. Morange, D. Moreno, M. Moreno Llácer, P. Morettini, S. Morgenstern, D. Mori, T. Mori, M. Morii, M. Morinaga, V. Morisbak, S. Moritz, A. K. Morley, G. Mornacchi, J. D. Morris, L. Morvaj, P. Moschovakos, M. Mosidze, H. J. Moss, J. Moss, K. Motohashi, R. Mount, E. Mountricha, E. J. W. Moyse, S. Muanza, R. D. Mudd, F. Mueller, J. Mueller, R. S. P. Mueller, T. Mueller, D. Muenstermann, P. Mullen, G. A. Mullier, F. J. Munoz Sanchez, J. A. Murillo Quijada, W. J. Murray, H. Musheghyan, M. Muškinja, A. G. Myagkov, M. Myska, B. P. Nachman, O. Nackenhorst, K. Nagai, R. Nagai, K. Nagano, Y. Nagasaka, K. Nagata, M. Nagel, E. Nagy, A. M. Nairz, Y. Nakahama, K. Nakamura, T. Nakamura, I. Nakano, R. F. Naranjo Garcia, R. Narayan, D. I. Narrias Villar, I. Naryshkin, T. Naumann, G. Navarro, R. Nayyar, H. A. Neal, P. Yu. Nechaeva, T. J. Neep, A. Negri, M. Negrini, S. Nektarijevic, C. Nellist, A. Nelson, S. Nemecek, P. Nemethy, A. A. Nepomuceno, M. Nessi, M. S. Neubauer, M. Neumann, R. M. Neves, P. Nevski, P. R. Newman, D. H. Nguyen, T. Nguyen Manh, R. B. Nickerson, R. Nicolaidou, J. Nielsen, V. Nikolaenko, I. Nikolic-Audit, K. Nikolopoulos, J. K. Nilsen, P. Nilsson, Y. Ninomiya, A. Nisati, R. Nisius, T. Nobe, M. Nomachi, I. Nomidis, T. Nooney, S. Norberg, M. Nordberg, N. Norjoharuddeen, O. Novgorodova, S. Nowak, M. Nozaki, L. Nozka, K. Ntekas, E. Nurse, F. Nuti, F. O’grady, D. C. O’Neil, A. A. O’Rourke, V. O’Shea, F. G. Oakham, H. Oberlack, T. Obermann, J. Ocariz, A. Ochi, I. Ochoa, J. P. Ochoa-Ricoux, S. Oda, S. Odaka, H. Ogren, A. Oh, S. H. Oh, C. C. Ohm, H. Ohman, H. Oide, H. Okawa, Y. Okumura, T. Okuyama, A. Olariu, L. F. Oleiro Seabra, S. A. Olivares Pino, D. Oliveira Damazio, A. Olszewski, J. Olszowska, A. Onofre, K. Onogi, P. U. E. Onyisi, M. J. Oreglia, Y. Oren, D. Orestano, N. Orlando, R. S. Orr, B. Osculati, R. Ospanov, G. Otero y Garzon, H. Otono, M. Ouchrif, F. Ould-Saada, A. Ouraou, K. P. Oussoren, Q. Ouyang, M. Owen, R. E. Owen, V. E. Ozcan, N. Ozturk, K. Pachal, A. Pacheco Pages, L. Pacheco Rodriguez, C. Padilla Aranda, S. Pagan Griso, M. Paganini, F. Paige, P. Pais, K. Pajchel, G. Palacino, S. Palazzo, S. Palestini, M. Palka, D. Pallin, E. St. Panagiotopoulou, I. Panagoulias, C. E. Pandini, J. G. Panduro Vazquez, P. Pani, S. Panitkin, D. Pantea, L. Paolozzi, Th. D. Papadopoulou, K. Papageorgiou, A. Paramonov, D. Paredes Hernandez, A. J. Parker, M. A. Parker, K. A. Parker, F. Parodi, J. A. Parsons, U. Parzefall, V. R. Pascuzzi, E. Pasqualucci, S. Passaggio, Fr. Pastore, G. Pásztor, S. Pataraia, J. R. Pater, T. Pauly, J. Pearce, B. Pearson, L. E. Pedersen, S. Pedraza Lopez, R. Pedro, S. V. Peleganchuk, O. Penc, C. Peng, H. Peng, J. Penwell, B. S. Peralva, M. M. Perego, D. V. Perepelitsa, E. Perez Codina, L. Perini, H. Pernegger, S. Perrella, R. Peschke, V. D. Peshekhonov, K. Peters, R. F. Y. Peters, B. A. Petersen, T. C. Petersen, E. Petit, A. Petridis, C. Petridou, P. Petroff, E. Petrolo, M. Petrov, F. Petrucci, N. E. Pettersson, A. Peyaud, R. Pezoa, P. W. Phillips, G. Piacquadio, E. Pianori, A. Picazio, E. Piccaro, M. Piccinini, M. A. Pickering, R. Piegaia, J. E. Pilcher, A. D. Pilkington, A. W. J. Pin, M. Pinamonti, J. L. Pinfold, A. Pingel, S. Pires, H. Pirumov, M. Pitt, L. Plazak, M. -A. Pleier, V. Pleskot, E. Plotnikova, D. Pluth, R. Poettgen, L. Poggioli, D. Pohl, G. Polesello, A. Poley, A. Policicchio, R. Polifka, A. Polini, C. S. Pollard, V. Polychronakos, K. Pommès, L. Pontecorvo, B. G. Pope, G. A. Popeneciu, A. Poppleton, S. Pospisil, K. Potamianos, I. N. Potrap, C. J. Potter, C. T. Potter, G. Poulard, J. Poveda, V. Pozdnyakov, M. E. Pozo Astigarraga, P. Pralavorio, A. Pranko, S. Prell, D. Price, L. E. Price, M. Primavera, S. Prince, K. Prokofiev, F. Prokoshin, S. Protopopescu, J. Proudfoot, M. Przybycien, D. Puddu, M. Purohit, P. Puzo, J. Qian, G. Qin, Y. Qin, A. Quadt, W. B. Quayle, M. Queitsch-Maitland, D. Quilty, S. Raddum, V. Radeka, V. Radescu, S. K. Radhakrishnan, P. Radloff, P. Rados, F. Ragusa, G. Rahal, J. A. Raine, S. Rajagopalan, M. Rammensee, C. Rangel-Smith, M. G. Ratti, D. M. Rauch, F. Rauscher, S. Rave, T. Ravenscroft, I. Ravinovich, M. Raymond, A. L. Read, N. P. Readioff, M. Reale, D. M. Rebuzzi, A. Redelbach, G. Redlinger, R. Reece, R. G. Reed, K. Reeves, L. Rehnisch, J. Reichert, A. Reiss, C. Rembser, H. Ren, M. Rescigno, S. Resconi, E. D. Resseguie, O. L. Rezanova, P. Reznicek, R. Rezvani, R. Richter, S. Richter, E. Richter-Was, O. Ricken, M. Ridel, P. Rieck, C. J. Riegel, J. Rieger, O. Rifki, M. Rijssenbeek, A. Rimoldi, M. Rimoldi, L. Rinaldi, B. Ristić, E. Ritsch, I. Riu, F. Rizatdinova, E. Rizvi, C. Rizzi, R. T. Roberts, S. H. Robertson, A. Robichaud-Veronneau, D. Robinson, J. E. M. Robinson, A. Robson, C. Roda, Y. Rodina, A. Rodriguez Perez, D. Rodriguez, S. Roe, C. S. Rogan, O. Røhne, J. Roloff, A. Romaniouk, M. Romano, S. M. Romano Saez, E. Romero Adam, N. Rompotis, M. Ronzani, L. Roos, E. Ros, S. Rosati, K. Rosbach, P. Rose, N. -A. Rosien, V. Rossetti, E. Rossi, L. P. Rossi, J. H. N. Rosten, R. Rosten, M. Rotaru, I. Roth, J. Rothberg, D. Rousseau, A. Rozanov, Y. Rozen, X. Ruan, F. Rubbo, M. S. Rudolph, F. Rühr, A. Ruiz-Martinez, Z. Rurikova, N. A. Rusakovich, A. Ruschke, H. L. Russell, J. P. Rutherfoord, N. Ruthmann, Y. F. Ryabov, M. Rybar, G. Rybkin, S. Ryu, A. Ryzhov, G. F. Rzehorz, A. F. Saavedra, G. Sabato, S. Sacerdoti, H. F-W. Sadrozinski, R. Sadykov, F. Safai Tehrani, P. Saha, M. Sahinsoy, M. Saimpert, T. Saito, H. Sakamoto, Y. Sakurai, G. Salamanna, A. Salamon, J. E. Salazar Loyola, D. Salek, P. H. Sales De Bruin, D. Salihagic, A. Salnikov, J. Salt, D. Salvatore, F. Salvatore, A. Salvucci, A. Salzburger, D. Sammel, D. Sampsonidis, J. Sánchez, V. Sanchez Martinez, A. Sanchez Pineda, H. Sandaker, R. L. Sandbach, M. Sandhoff, C. Sandoval, D. P. C. Sankey, M. Sannino, A. Sansoni, C. Santoni, R. Santonico, H. Santos, I. Santoyo Castillo, K. Sapp, A. Sapronov, J. G. Saraiva, B. Sarrazin, O. Sasaki, K. Sato, E. Sauvan, G. Savage, P. Savard, N. Savic, C. Sawyer, L. Sawyer, J. Saxon, C. Sbarra, A. Sbrizzi, T. Scanlon, D. A. Scannicchio, M. Scarcella, V. Scarfone, J. Schaarschmidt, P. Schacht, B. M. Schachtner, D. Schaefer, L. Schaefer, R. Schaefer, J. Schaeffer, S. Schaepe, S. Schaetzel, U. Schäfer, A. C. Schaffer, D. Schaile, R. D. Schamberger, V. Scharf, V. A. Schegelsky, D. Scheirich, M. Schernau, C. Schiavi, S. Schier, C. Schillo, M. Schioppa, S. Schlenker, K. R. Schmidt-Sommerfeld, K. Schmieden, C. Schmitt, S. Schmitt, S. Schmitz, B. Schneider, U. Schnoor, L. Schoeffel, A. Schoening, B. D. Schoenrock, E. Schopf, M. Schott, J. F. P. Schouwenberg, J. Schovancova, S. Schramm, M. Schreyer, N. Schuh, A. Schulte, M. J. Schultens, H. -C. Schultz-Coulon, H. Schulz, M. Schumacher, B. A. Schumm, Ph. Schune, A. Schwartzman, T. A. Schwarz, H. Schweiger, Ph. Schwemling, R. Schwienhorst, J. Schwindling, T. Schwindt, G. Sciolla, F. Scuri, F. Scutti, J. Searcy, P. Seema, S. C. Seidel, A. Seiden, F. Seifert, J. M. Seixas, G. Sekhniaidze, K. Sekhon, S. J. Sekula, D. M. Seliverstov, N. Semprini-Cesari, C. Serfon, L. Serin, L. Serkin, T. Serre, M. Sessa, R. Seuster, H. Severini, T. Sfiligoj, F. Sforza, A. Sfyrla, E. Shabalina, N. W. Shaikh, L. Y. Shan, R. Shang, J. T. Shank, M. Shapiro, P. B. Shatalov, K. Shaw, S. M. Shaw, A. Shcherbakova, C. Y. Shehu, P. Sherwood, L. Shi, S. Shimizu, C. O. Shimmin, M. Shimojima, S. Shirabe, M. Shiyakova, A. Shmeleva, D. Shoaleh Saadi, M. J. Shochet, S. Shojaii, D. R. Shope, S. Shrestha, E. Shulga, M. A. Shupe, P. Sicho, A. M. Sickles, P. E. Sidebo, E. Sideras Haddad, O. Sidiropoulou, D. Sidorov, A. Sidoti, F. Siegert, Dj. Sijacki, J. Silva, S. B. Silverstein, V. Simak, Lj. Simic, S. Simion, E. Simioni, B. Simmons, D. Simon, M. Simon, P. Sinervo, N. B. Sinev, M. Sioli, G. Siragusa, I. Siral, S. Yu. Sivoklokov, J. Sjölin, M. B. Skinner, H. P. Skottowe, P. Skubic, M. Slater, T. Slavicek, M. Slawinska, K. Sliwa, R. Slovak, V. Smakhtin, B. H. Smart, L. Smestad, J. Smiesko, S. Yu. Smirnov, Y. Smirnov, L. N. Smirnova, O. Smirnova, J. W. Smith, M. N. K. Smith, R. W. Smith, M. Smizanska, K. Smolek, A. A. Snesarev, I. M. Snyder, S. Snyder, R. Sobie, F. Socher, A. Soffer, D. A. Soh, G. Sokhrannyi, C. A. Solans Sanchez, M. Solar, E. Yu. Soldatov, U. Soldevila, A. A. Solodkov, A. Soloshenko, O. V. Solovyanov, V. Solovyev, P. Sommer, H. Son, H. Y. Song, A. Sood, A. Sopczak, V. Sopko, V. Sorin, D. Sosa, C. L. Sotiropoulou, R. Soualah, A. M. Soukharev, D. South, B. C. Sowden, S. Spagnolo, M. Spalla, M. Spangenberg, F. Spanò, D. Sperlich, F. Spettel, R. Spighi, G. Spigo, L. A. Spiller, M. Spousta, R. D. St. Denis, A. Stabile, R. Stamen, S. Stamm, E. Stanecka, R. W. Stanek, C. Stanescu, M. Stanescu-Bellu, M. M. Stanitzki, S. Stapnes, E. A. Starchenko, G. H. Stark, J. Stark, S. H. Stark, P. Staroba, P. Starovoitov, S. Stärz, R. Staszewski, P. Steinberg, B. Stelzer, H. J. Stelzer, O. Stelzer-Chilton, H. Stenzel, G. A. Stewart, J. A. Stillings, M. C. Stockton, M. Stoebe, G. Stoicea, P. Stolte, S. Stonjek, A. R. Stradling, A. Straessner, M. E. Stramaglia, J. Strandberg, S. Strandberg, A. Strandlie, M. Strauss, P. Strizenec, R. Ströhmer, D. M. Strom, R. Stroynowski, A. Strubig, S. A. Stucci, B. Stugu, N. A. Styles, D. Su, J. Su, S. Suchek, Y. Sugaya, M. Suk, V. V. Sulin, S. Sultansoy, T. Sumida, S. Sun, X. Sun, J. E. Sundermann, K. Suruliz, C. J. E. Suster, M. R. Sutton, S. Suzuki, M. Svatos, M. Swiatlowski, S. P. Swift, I. Sykora, T. Sykora, D. Ta, K. Tackmann, J. Taenzer, A. Taffard, R. Tafirout, N. Taiblum, H. Takai, R. Takashima, T. Takeshita, Y. Takubo, M. Talby, A. A. Talyshev, J. Tanaka, M. Tanaka, R. Tanaka, S. Tanaka, R. Tanioka, B. B. Tannenwald, S. Tapia Araya, S. Tapprogge, S. Tarem, G. F. Tartarelli, P. Tas, M. Tasevsky, T. Tashiro, E. Tassi, A. Tavares Delgado, Y. Tayalati, A. C. Taylor, G. N. Taylor, P. T. E. Taylor, W. Taylor, F. A. Teischinger, P. Teixeira-Dias, K. K. Temming, D. Temple, H. Ten Kate, P. K. Teng, J. J. Teoh, F. Tepel, S. Terada, K. Terashi, J. Terron, S. Terzo, M. Testa, R. J. Teuscher, T. Theveneaux-Pelzer, J. P. Thomas, J. Thomas-Wilsker, P. D. Thompson, A. S. Thompson, L. A. Thomsen, E. Thomson, M. J. Tibbetts, R. E. Ticse Torres, V. O. Tikhomirov, Yu. A. Tikhonov, S. Timoshenko, E. Tiouchichine, P. Tipton, S. Tisserant, K. Todome, T. Todorov, S. Todorova-Nova, J. Tojo, S. Tokár, K. Tokushuku, E. Tolley, L. Tomlinson, M. Tomoto, L. Tompkins, K. Toms, B. Tong, P. Tornambe, E. Torrence, H. Torres, E. Torró Pastor, J. Toth, F. Touchard, D. R. Tovey, T. Trefzger, A. Tricoli, I. M. Trigger, S. Trincaz-Duvoid, M. F. Tripiana, W. Trischuk, B. Trocmé, A. Trofymov, C. Troncon, M. Trottier-McDonald, M. Trovatelli, L. Truong, M. Trzebinski, A. Trzupek, J. C-L. Tseng, P. V. Tsiareshka, G. Tsipolitis, N. Tsirintanis, S. Tsiskaridze, V. Tsiskaridze, E. G. Tskhadadze, K. M. Tsui, I. I. Tsukerman, V. Tsulaia, S. Tsuno, D. Tsybychev, Y. Tu, A. Tudorache, V. Tudorache, T. T. Tulbure, A. N. Tuna, S. A. Tupputi, S. Turchikhin, D. Turgeman, I. Turk Cakir, R. Turra, P. M. Tuts, G. Ucchielli, I. Ueda, M. Ughetto, F. Ukegawa, G. Unal, A. Undrus, G. Unel, F. C. Ungaro, Y. Unno, C. Unverdorben, J. Urban, P. Urquijo, P. Urrejola, G. Usai, J. Usui, L. Vacavant, V. Vacek, B. Vachon, C. Valderanis, E. Valdes Santurio, N. Valencic, S. Valentinetti, A. Valero, L. Valery, S. Valkar, J. A. Valls Ferrer, W. Van Den Wollenberg, P. C. Van Der Deijl, H. van der Graaf, N. van Eldik, P. van Gemmeren, J. Van Nieuwkoop, I. van Vulpen, M. C. van Woerden, M. Vanadia, W. Vandelli, R. Vanguri, A. Vaniachine, P. Vankov, G. Vardanyan, R. Vari, E. W. Varnes, T. Varol, D. Varouchas, A. Vartapetian, K. E. Varvell, J. G. Vasquez, G. A. Vasquez, F. Vazeille, T. Vazquez Schroeder, J. Veatch, V. Veeraraghavan, L. M. Veloce, F. Veloso, S. Veneziano, A. Ventura, M. Venturi, N. Venturi, A. Venturini, V. Vercesi, M. Verducci, W. Verkerke, J. C. Vermeulen, A. Vest, M. C. Vetterli, O. Viazlo, I. Vichou, T. Vickey, O. E. Vickey Boeriu, G. H. A. Viehhauser, S. Viel, L. Vigani, M. Villa, M. Villaplana Perez, E. Vilucchi, M. G. Vincter, V. B. Vinogradov, C. Vittori, I. Vivarelli, S. Vlachos, M. Vlasak, M. Vogel, P. Vokac, G. Volpi, M. Volpi, H. von der Schmitt, E. von Toerne, V. Vorobel, K. Vorobev, M. Vos, R. Voss, J. H. Vossebeld, N. Vranjes, M. Vranjes Milosavljevic, V. Vrba, M. Vreeswijk, R. Vuillermet, I. Vukotic, P. Wagner, W. Wagner, H. Wahlberg, S. Wahrmund, J. Wakabayashi, J. Walder, R. Walker, W. Walkowiak, V. Wallangen, C. Wang, C. Wang, F. Wang, H. Wang, H. Wang, J. Wang, J. Wang, K. Wang, R. Wang, S. M. Wang, T. Wang, W. Wang, C. Wanotayaroj, A. Warburton, C. P. Ward, D. R. Wardrope, A. Washbrook, P. M. Watkins, A. T. Watson, M. F. Watson, G. Watts, S. Watts, B. M. Waugh, S. Webb, M. S. Weber, S. W. Weber, S. A. Weber, J. S. Webster, A. R. Weidberg, B. Weinert, J. Weingarten, C. Weiser, H. Weits, P. S. Wells, T. Wenaus, T. Wengler, S. Wenig, N. Wermes, M. D. Werner, P. Werner, M. Wessels, J. Wetter, K. Whalen, N. L. Whallon, A. M. Wharton, A. White, M. J. White, R. White, D. Whiteson, F. J. Wickens, W. Wiedenmann, M. Wielers, C. Wiglesworth, L. A. M. Wiik-Fuchs, A. Wildauer, F. Wilk, H. G. Wilkens, H. H. Williams, S. Williams, C. Willis, S. Willocq, J. A. Wilson, I. Wingerter-Seez, F. Winklmeier, O. J. Winston, B. T. Winter, M. Wittgen, T. M. H. Wolf, R. Wolff, M. W. Wolter, H. Wolters, S. D. Worm, B. K. Wosiek, J. Wotschack, M. J. Woudstra, K. W. Wozniak, M. Wu, M. Wu, S. L. Wu, X. Wu, Y. Wu, T. R. Wyatt, B. M. Wynne, S. Xella, Z. Xi, D. Xu, L. Xu, B. Yabsley, S. Yacoob, D. Yamaguchi, Y. Yamaguchi, A. Yamamoto, S. Yamamoto, T. Yamanaka, K. Yamauchi, Y. Yamazaki, Z. Yan, H. Yang, H. Yang, Y. Yang, Z. Yang, W-M. Yao, Y. C. Yap, Y. Yasu, E. Yatsenko, K. H. Yau Wong, J. Ye, S. Ye, I. Yeletskikh, E. Yildirim, K. Yorita, R. Yoshida, K. Yoshihara, C. Young, C. J. S. Young, S. Youssef, D. R. Yu, J. Yu, J. M. Yu, J. Yu, L. Yuan, S. P. Y. Yuen, I. Yusuff, B. Zabinski, G. Zacharis, R. Zaidan, A. M. Zaitsev, N. Zakharchuk, J. Zalieckas, A. Zaman, S. Zambito, L. Zanello, D. Zanzi, C. Zeitnitz, M. Zeman, A. Zemla, J. C. Zeng, Q. Zeng, O. Zenin, T. Ženiš, D. Zerwas, D. Zhang, F. Zhang, G. Zhang, H. Zhang, J. Zhang, L. Zhang, L. Zhang, M. Zhang, R. Zhang, R. Zhang, X. Zhang, Y. Zhang, Z. Zhang, X. Zhao, Y. Zhao, Z. Zhao, A. Zhemchugov, J. Zhong, B. Zhou, C. Zhou, L. Zhou, L. Zhou, M. Zhou, M. Zhou, N. Zhou, C. G. Zhu, H. Zhu, J. Zhu, Y. Zhu, X. Zhuang, K. Zhukov, A. Zibell, D. Zieminska, N. I. Zimine, C. Zimmermann, S. Zimmermann, Z. Zinonos, M. Zinser, M. Ziolkowski, L. Živković, G. Zobernig, A. Zoccoli, M. Zur Nedden, L. Zwalinski, ATLAS Collaboration

**Affiliations:** 1 Department of Physics, University of Adelaide, Adelaide, SA Australia; 2 Physics Department, SUNY Albany, Albany, NY USA; 3 Department of Physics, University of Alberta, Edmonton, AB Canada; 4 Department of Physics, Ankara University, Ankara, Turkey; 5 Istanbul Aydin University, Istanbul, Turkey; 6 Division of Physics, TOBB University of Economics and Technology, Ankara, Turkey; 7 LAPP, CNRS/IN2P3 and Université Savoie Mont Blanc, Annecy-le-Vieux, France; 8 High Energy Physics Division, Argonne National Laboratory, Argonne, IL USA; 9 Department of Physics, University of Arizona, Tucson, AZ USA; 10 Department of Physics, The University of Texas at Arlington, Arlington, TX USA; 11 Physics Department, National and Kapodistrian University of Athens, Athens, Greece; 12 Physics Department, National Technical University of Athens, Zografou, Greece; 13 Department of Physics, The University of Texas at Austin, Austin, TX USA; 14 Institute of Physics, Azerbaijan Academy of Sciences, Baku, Azerbaijan; 15 Institut de Física d’Altes Energies (IFAE), The Barcelona Institute of Science and Technology, Barcelona, Spain; 16 Institute of Physics, University of Belgrade, Belgrade, Serbia; 17 Department for Physics and Technology, University of Bergen, Bergen, Norway; 18 Physics Division, Lawrence Berkeley National Laboratory and University of California, Berkeley, CA USA; 19 Department of Physics, Humboldt University, Berlin, Germany; 20 Albert Einstein Center for Fundamental Physics and Laboratory for High Energy Physics, University of Bern, Bern, Switzerland; 21 School of Physics and Astronomy, University of Birmingham, Birmingham, UK; 22 Department of Physics, Bogazici University, Istanbul, Turkey; 23 Department of Physics Engineering, Gaziantep University, Gaziantep, Turkey; 24 Istanbul Bilgi University, Faculty of Engineering and Natural Sciences, Istanbul, Turkey; 25 Bahcesehir University, Faculty of Engineering and Natural Sciences, Istanbul, Turkey; 26 Centro de Investigaciones, Universidad Antonio Narino, Bogota, Colombia; 27 INFN Sezione di Bologna, Bologna, Italy; 28 Dipartimento di Fisica e Astronomia, Università di Bologna, Bologna, Italy; 29 Physikalisches Institut, University of Bonn, Bonn, Germany; 30 Department of Physics, Boston University, Boston, MA USA; 31 Department of Physics, Brandeis University, Waltham, MA USA; 32 Universidade Federal do Rio De Janeiro COPPE/EE/IF, Rio de Janeiro, Brazil; 33 Electrical Circuits Department, Federal University of Juiz de Fora (UFJF), Juiz de Fora, Brazil; 34 Federal University of Sao Joao del Rei (UFSJ), Sao Joao del Rei, Brazil; 35 Instituto de Fisica, Universidade de Sao Paulo, Sao Paulo, Brazil; 36 Physics Department, Brookhaven National Laboratory, Upton, NY USA; 37 Transilvania University of Brasov, Brasov, Romania; 38 Horia Hulubei National Institute of Physics and Nuclear Engineering, Bucharest, Romania; 39 Physics Department, National Institute for Research and Development of Isotopic and Molecular Technologies, Cluj Napoca, Romania; 40 University Politehnica Bucharest, Bucharest, Romania; 41 West University in Timisoara, Timisoara, Romania; 42 Departamento de Física, Universidad de Buenos Aires, Buenos Aires, Argentina; 43 Cavendish Laboratory, University of Cambridge, Cambridge, UK; 44 Department of Physics, Carleton University, Ottawa, ON Canada; 45 CERN, Geneva, Switzerland; 46 Enrico Fermi Institute, University of Chicago, Chicago, IL USA; 47 Departamento de Física, Pontificia Universidad Católica de Chile, Santiago, Chile; 48 Departamento de Física, Universidad Técnica Federico Santa María, Valparaiso, Chile; 49 Institute of High Energy Physics, Chinese Academy of Sciences, Beijing, China; 50 Department of Physics, Nanjing University, Jiangsu, China; 51 Physics Department, Tsinghua University, Beijing, 100084 China; 52 Department of Modern Physics, University of Science and Technology of China, Anhui, China; 53 School of Physics, Shandong University, Shandong, China; 54 Department of Physics and Astronomy, Key Laboratory for Particle Physics, Astrophysics and Cosmology, Ministry of Education, Shanghai Key Laboratory for Particle Physics and Cosmology, Shanghai Jiao Tong University (also at PKU-CHEP), Shanghai, China; 55 Laboratoire de Physique de Clermont-Ferrand (LPC), Université Clermont Auvergne, CNRS/IN2P3, Clermont-Ferrand, France; 56 Nevis Laboratory, Columbia University, Irvington, NY USA; 57 Niels Bohr Institute, University of Copenhagen, Copenhagen, Denmark; 58 INFN Gruppo Collegato di Cosenza, Laboratori Nazionali di Frascati, Frascati, Italy; 59 Dipartimento di Fisica, Università della Calabria, Rende, Italy; 60 Faculty of Physics and Applied Computer Science, AGH University of Science and Technology, Kraków, Poland; 61 Marian Smoluchowski Institute of Physics, Jagiellonian University, Kraków, Poland; 62 Institute of Nuclear Physics, Polish Academy of Sciences, Kraków, Poland; 63 Physics Department, Southern Methodist University, Dallas, TX USA; 64 Physics Department, University of Texas at Dallas, Richardson, TX USA; 65 DESY, Hamburg and Zeuthen, Germany; 66 Lehrstuhl für Experimentelle Physik IV, Technische Universität Dortmund, Dortmund, Germany; 67 Institut für Kern-und Teilchenphysik, Technische Universität Dresden, Dresden, Germany; 68 Department of Physics, Duke University, Durham, NC USA; 69 SUPA-School of Physics and Astronomy, University of Edinburgh, Edinburgh, UK; 70 INFN Laboratori Nazionali di Frascati, Frascati, Italy; 71 Fakultät für Mathematik und Physik, Albert-Ludwigs-Universität, Freiburg, Germany; 72 Departement de Physique Nucleaire et Corpusculaire, Université de Genève, Geneva, Switzerland; 73 INFN Sezione di Genova, Genoa, Italy; 74 Dipartimento di Fisica, Università di Genova, Genoa, Italy; 75 E. Andronikashvili Institute of Physics, Iv. Javakhishvili Tbilisi State University, Tbilisi, Georgia; 76 High Energy Physics Institute, Tbilisi State University, Tbilisi, Georgia; 77 II Physikalisches Institut, Justus-Liebig-Universität Giessen, Giessen, Germany; 78 SUPA-School of Physics and Astronomy, University of Glasgow, Glasgow, UK; 79 II Physikalisches Institut, Georg-August-Universität, Göttingen, Germany; 80 Laboratoire de Physique Subatomique et de Cosmologie, Université Grenoble-Alpes, CNRS/IN2P3, Grenoble, France; 81 Laboratory for Particle Physics and Cosmology, Harvard University, Cambridge, MA USA; 82 Kirchhoff-Institut für Physik, Ruprecht-Karls-Universität Heidelberg, Heidelberg, Germany; 83 Physikalisches Institut, Ruprecht-Karls-Universität Heidelberg, Heidelberg, Germany; 84 ZITI Institut für technische Informatik, Ruprecht-Karls-Universität Heidelberg, Mannheim, Germany; 85 Faculty of Applied Information Science, Hiroshima Institute of Technology, Hiroshima, Japan; 86 Department of Physics, The Chinese University of Hong Kong, Shatin, NT Hong Kong; 87 Department of Physics, The University of Hong Kong, Hong Kong, China; 88 Department of Physics and Institute for Advanced Study, The Hong Kong University of Science and Technology, Clear Water Bay, Kowloon, Hong Kong, China; 89 Department of Physics, National Tsing Hua University, Hsinchu, Taiwan Taiwan; 90 Department of Physics, Indiana University, Bloomington, IN USA; 91 Institut für Astro- und Teilchenphysik, Leopold-Franzens-Universität, Innsbruck, Austria; 92 University of Iowa, Iowa City, IA USA; 93 Department of Physics and Astronomy, Iowa State University, Ames, IA USA; 94 Joint Institute for Nuclear Research, JINR Dubna, Dubna, Russia; 95 KEK, High Energy Accelerator Research Organization, Tsukuba, Japan; 96 Graduate School of Science, Kobe University, Kobe, Japan; 97 Faculty of Science, Kyoto University, Kyoto, Japan; 98 Kyoto University of Education, Kyoto, Japan; 99 Department of Physics, Kyushu University, Fukuoka, Japan; 100 Instituto de Física La Plata, Universidad Nacional de La Plata and CONICET, La Plata, Argentina; 101 Physics Department, Lancaster University, Lancaster, UK; 102 INFN Sezione di Lecce, Lecce, Italy; 103 Dipartimento di Matematica e Fisica, Università del Salento, Lecce, Italy; 104 Oliver Lodge Laboratory, University of Liverpool, Liverpool, UK; 105 Department of Experimental Particle Physics, Jožef Stefan Institute and Department of Physics, University of Ljubljana, Ljubljana, Slovenia; 106 School of Physics and Astronomy, Queen Mary University of London, London, UK; 107 Department of Physics, Royal Holloway University of London, Surrey, UK; 108 Department of Physics and Astronomy, University College London, London, UK; 109 Louisiana Tech University, Ruston, LA USA; 110 Laboratoire de Physique Nucléaire et de Hautes Energies, UPMC and Université Paris-Diderot and CNRS/IN2P3, Paris, France; 111 Fysiska institutionen, Lunds universitet, Lund, Sweden; 112 Departamento de Fisica Teorica C-15, Universidad Autonoma de Madrid, Madrid, Spain; 113 Institut für Physik, Universität Mainz, Mainz, Germany; 114 School of Physics and Astronomy, University of Manchester, Manchester, UK; 115 CPPM, Aix-Marseille Université and CNRS/IN2P3, Marseille, France; 116 Department of Physics, University of Massachusetts, Amherst, MA USA; 117 Department of Physics, McGill University, Montreal, QC Canada; 118 School of Physics, University of Melbourne, Melbourne, VIC Australia; 119 Department of Physics, The University of Michigan, Ann Arbor, MI USA; 120 Department of Physics and Astronomy, Michigan State University, East Lansing, MI USA; 121 INFN Sezione di Milano, Milan, Italy; 122 Dipartimento di Fisica, Università di Milano, Milan, Italy; 123 B.I. Stepanov Institute of Physics, National Academy of Sciences of Belarus, Minsk, Republic of Belarus; 124 Research Institute for Nuclear Problems of Byelorussian State University, Minsk, Republic of Belarus; 125 Group of Particle Physics, University of Montreal, Montreal, QC Canada; 126 P.N. Lebedev Physical Institute of the Russian Academy of Sciences, Moscow, Russia; 127 Institute for Theoretical and Experimental Physics (ITEP), Moscow, Russia; 128 National Research Nuclear University MEPhI, Moscow, Russia; 129 D.V. Skobeltsyn Institute of Nuclear Physics, M.V. Lomonosov Moscow State University, Moscow, Russia; 130 Fakultät für Physik, Ludwig-Maximilians-Universität München, Munich, Germany; 131 Max-Planck-Institut für Physik (Werner-Heisenberg-Institut), Munich, Germany; 132 Nagasaki Institute of Applied Science, Nagasaki, Japan; 133 Graduate School of Science and Kobayashi-Maskawa Institute, Nagoya University, Nagoya, Japan; 134 INFN Sezione di Napoli, Naples, Italy; 135 Dipartimento di Fisica, Università di Napoli, Naples, Italy; 136 Department of Physics and Astronomy, University of New Mexico, Albuquerque, NM USA; 137 Institute for Mathematics, Astrophysics and Particle Physics, Radboud University Nijmegen/Nikhef, Nijmegen, The Netherlands; 138 Nikhef National Institute for Subatomic Physics and University of Amsterdam, Amsterdam, The Netherlands; 139 Department of Physics, Northern Illinois University, DeKalb, IL USA; 140 Budker Institute of Nuclear Physics, SB RAS, Novosibirsk, Russia; 141 Department of Physics, New York University, New York, NY USA; 142 Ohio State University, Columbus, OH USA; 143 Faculty of Science, Okayama University, Okayama, Japan; 144 Homer L. Dodge Department of Physics and Astronomy, University of Oklahoma, Norman, OK USA; 145 Department of Physics, Oklahoma State University, Stillwater, OK USA; 146 Palacký University, RCPTM, Olomouc, Czech Republic; 147 Center for High Energy Physics, University of Oregon, Eugene, OR USA; 148 LAL, Univ. Paris-Sud, CNRS/IN2P3, Université Paris-Saclay, Orsay, France; 149 Graduate School of Science, Osaka University, Osaka, Japan; 150 Department of Physics, University of Oslo, Oslo, Norway; 151 Department of Physics, Oxford University, Oxford, UK; 152 INFN Sezione di Pavia, Pavia, Italy; 153 Dipartimento di Fisica, Università di Pavia, Pavia, Italy; 154 Department of Physics, University of Pennsylvania, Philadelphia, PA USA; 155 National Research Centre “Kurchatov Institute” B.P.Konstantinov Petersburg Nuclear Physics Institute, St. Petersburg, Russia; 156 INFN Sezione di Pisa, Pisa, Italy; 157 Dipartimento di Fisica E. Fermi, Università di Pisa, Pisa, Italy; 158 Department of Physics and Astronomy, University of Pittsburgh, Pittsburgh, PA USA; 159 Laboratório de Instrumentação e Física Experimental de Partículas-LIP, Lisbon, Portugal; 160 Faculdade de Ciências, Universidade de Lisboa, Lisbon, Portugal; 161 Department of Physics, University of Coimbra, Coimbra, Portugal; 162 Centro de Física Nuclear da Universidade de Lisboa, Lisbon, Portugal; 163 Departamento de Fisica, Universidade do Minho, Braga, Portugal; 164 Departamento de Fisica Teorica y del Cosmos and CAFPE, Universidad de Granada, Granada, Spain; 165 Dep Fisica and CEFITEC of Faculdade de Ciencias e Tecnologia, Universidade Nova de Lisboa, Caparica, Portugal; 166 Institute of Physics, Academy of Sciences of the Czech Republic, Prague, Czech Republic; 167 Czech Technical University in Prague, Prague, Czech Republic; 168 Charles University, Faculty of Mathematics and Physics, Prague, Czech Republic; 169 State Research Center Institute for High Energy Physics (Protvino), NRC KI, Protvino, Russia; 170 Particle Physics Department, Rutherford Appleton Laboratory, Didcot, UK; 171 INFN Sezione di Roma, Rome, Italy; 172 Dipartimento di Fisica, Sapienza Università di Roma, Rome, Italy; 173 INFN Sezione di Roma Tor Vergata, Rome, Italy; 174 Dipartimento di Fisica, Università di Roma Tor Vergata, Rome, Italy; 175 INFN Sezione di Roma Tre, Rome, Italy; 176 Dipartimento di Matematica e Fisica, Università Roma Tre, Rome, Italy; 177 Faculté des Sciences Ain Chock, Réseau Universitaire de Physique des Hautes Energies-Université Hassan II, Casablanca, Morocco; 178 Centre National de l’Energie des Sciences Techniques Nucleaires, Rabat, Morocco; 179 Faculté des Sciences Semlalia, Université Cadi Ayyad, LPHEA-Marrakech, Marrakech, Morocco; 180 Faculté des Sciences, Université Mohamed Premier and LPTPM, Oujda, Morocco; 181 Faculté des Sciences, Université Mohammed V, Rabat, Morocco; 182 DSM/IRFU (Institut de Recherches sur les Lois Fondamentales de l’Univers), CEA Saclay (Commissariat à l’Energie Atomique et aux Energies Alternatives), Gif-sur-Yvette, France; 183 Santa Cruz Institute for Particle Physics, University of California Santa Cruz, Santa Cruz, CA USA; 184 Department of Physics, University of Washington, Seattle, WA USA; 185 Department of Physics and Astronomy, University of Sheffield, Sheffield, UK; 186 Department of Physics, Shinshu University, Nagano, Japan; 187 Fachbereich Physik, Universität Siegen, Siegen, Germany; 188 Department of Physics, Simon Fraser University, Burnaby, BC Canada; 189 SLAC National Accelerator Laboratory, Stanford, CA USA; 190 Faculty of Mathematics, Physics and Informatics, Comenius University, Bratislava, Slovak Republic; 191 Department of Subnuclear Physics, Institute of Experimental Physics of the Slovak Academy of Sciences, Kosice, Slovak Republic; 192 Department of Physics, University of Cape Town, Cape Town, South Africa; 193 Department of Physics, University of Johannesburg, Johannesburg, South Africa; 194 School of Physics, University of the Witwatersrand, Johannesburg, South Africa; 195 Department of Physics, Stockholm University, Stockholm, Sweden; 196 The Oskar Klein Centre, Stockholm, Sweden; 197 Physics Department, Royal Institute of Technology, Stockholm, Sweden; 198 Departments of Physics and Astronomy and Chemistry, Stony Brook University, Stony Brook, NY USA; 199 Department of Physics and Astronomy, University of Sussex, Brighton, UK; 200 School of Physics, University of Sydney, Sydney, NSW Australia; 201 Institute of Physics, Academia Sinica, Taipei, Taiwan; 202 Department of Physics, Technion: Israel Institute of Technology, Haifa, Israel; 203 Raymond and Beverly Sackler School of Physics and Astronomy, Tel Aviv University, Tel Aviv, Israel; 204 Department of Physics, Aristotle University of Thessaloniki, Thessaloniki, Greece; 205 International Center for Elementary Particle Physics and Department of Physics, The University of Tokyo, Tokyo, Japan; 206 Graduate School of Science and Technology, Tokyo Metropolitan University, Tokyo, Japan; 207 Department of Physics, Tokyo Institute of Technology, Tokyo, Japan; 208 Tomsk State University, Tomsk, Russia; 209 Department of Physics, University of Toronto, Toronto, ON Canada; 210 INFN-TIFPA, Povo, Italy; 211 University of Trento, Trento, Italy; 212 TRIUMF, Vancouver, BC Canada; 213 Department of Physics and Astronomy, York University, Toronto, ON Canada; 214 Faculty of Pure and Applied Sciences, and Center for Integrated Research in Fundamental Science and Engineering, University of Tsukuba, Tsukuba, Japan; 215 Department of Physics and Astronomy, Tufts University, Medford, MA USA; 216 Department of Physics and Astronomy, University of California Irvine, Irvine, CA USA; 217 INFN Gruppo Collegato di Udine, Sezione di Trieste, Udine, Italy; 218 ICTP, Trieste, Italy; 219 Dipartimento di Chimica Fisica e Ambiente, Università di Udine, Udine, Italy; 220 Department of Physics and Astronomy, University of Uppsala, Uppsala, Sweden; 221 Department of Physics, University of Illinois, Urbana, IL USA; 222 Instituto de Fisica Corpuscular (IFIC) and Departamento de Fisica Atomica, Molecular y Nuclear and Departamento de Ingeniería Electrónica and Instituto de Microelectrónica de Barcelona (IMB-CNM), University of Valencia and CSIC, Valencia, Spain; 223 Department of Physics, University of British Columbia, Vancouver, BC Canada; 224 Department of Physics and Astronomy, University of Victoria, Victoria, BC Canada; 225 Department of Physics, University of Warwick, Coventry, UK; 226 Waseda University, Tokyo, Japan; 227 Department of Particle Physics, The Weizmann Institute of Science, Rehovot, Israel; 228 Department of Physics, University of Wisconsin, Madison, WI USA; 229 Fakultät für Physik und Astronomie, Julius-Maximilians-Universität, Würzburg, Germany; 230 Fakultät für Mathematik und Naturwissenschaften, Fachgruppe Physik, Bergische Universität Wuppertal, Wuppertal, Germany; 231 Department of Physics, Yale University, New Haven, CT USA; 232 Yerevan Physics Institute, Yerevan, Armenia; 233 Centre de Calcul de l’Institut National de Physique Nucléaire et de Physique des Particules (IN2P3), Villeurbanne, France; 234 CERN, 1211 Geneva 23, Switzerland

## Abstract

This paper describes the algorithms for the reconstruction and identification of electrons in the central region of the ATLAS detector at the Large Hadron Collider (LHC). These algorithms were used for all ATLAS results with electrons in the final state that are based on the 2012 *pp* collision data produced by the LHC at $\sqrt{\mathrm {s}}$ = 8 $\text {TeV}$. The efficiency of these algorithms, together with the charge misidentification rate, is measured in data and evaluated in simulated samples using electrons from $Z\rightarrow ee$, $Z\rightarrow ee\gamma$ and $J/\psi \rightarrow ee$ decays. For these efficiency measurements, the full recorded data set, corresponding to an integrated luminosity of 20.3 fb$^{-1}$, is used. Based on a new reconstruction algorithm used in 2012, the electron reconstruction efficiency is 97% for electrons with $E_{\mathrm {T}}=15$ $\text {GeV}$ and 99% at $E_{\mathrm {T}}= 50$ $\text {GeV}$. Combining this with the efficiency of additional selection criteria to reject electrons from background processes or misidentified hadrons, the efficiency to reconstruct and identify electrons at the ATLAS experiment varies from 65 to 95%, depending on the transverse momentum of the electron and background rejection.

## Introduction

In the ATLAS detector [1], electrons in the central detector region are triggered by and reconstructed from energy deposits in the electromagnetic (EM) calorimeter that are matched to a track in the inner detector (ID). Electrons are distinguished from other particles using identification criteria with different levels of background rejection and signal efficiency. The identification criteria rely on the shapes of EM showers in the calorimeter as well as on tracking quantities and the quality of the matching of the tracks to the clustered energy deposits in the calorimeter. They are based either on independent requirements or on a single requirement, the output of a likelihood function built from these quantities.

In this document, measurements of the efficiency to reconstruct and identify prompt electrons and their EM charge in the central region of the ATLAS detector^1^ with pseudorapidity $|\eta |<2.47$ are presented for *pp* collision data produced by the Large Hadron Collider (LHC) in 2012 at a centre-of-mass energy of $\sqrt{\mathrm {s}}$ = 8 $\text {TeV}$, and compared to the prediction from Monte Carlo (MC) simulation. The goal is to extract correction factors and their uncertainties for measurements of final states with prompt electrons in order to adjust the MC efficiencies to those measured in data. Electrons from semileptonic heavy-flavour decays are treated as background.

The efficiency measurements follow the methods introduced in Ref. [2] for the 2011 ATLAS electron performance studies but are improved in several respects and adjusted for the 2012 data-taking conditions. The measurements are based on the tag-and-probe method using the $Z$ and the $J/\psi$ resonances, requiring the presence of an isolated identified electron as the *tag*. Additional selection criteria are applied to obtain a high purity sample of electron candidates that can be used as *probes* to measure the reconstruction or identification efficiency. The measurements span different but overlapping kinematic regions and are studied as a function of the electron’s transverse momentum and pseudorapidity. The results are combined taking into account bin-to-bin correlations.

After briefly describing the ATLAS detector in Sect. 2, the algorithms to reconstruct and identify electrons are summarized in Sects. 3 and 4. The general methodology of tag-and-probe efficiency measurements and the decomposition of the efficiency into its different components are reviewed in Sect. 5. The data and MC samples used in this work are summarized in Sect. 6. Sections 7 and 8 describe the identification efficiency measurements for signal electrons as well as backgrounds. In Sect. 9, the measurement of the electron charge misidentification rate is presented. Section 10 details the reconstruction efficiency measurement, which extends the identification measurement methodology, and Sect. 11 describes the final results of the combined identification and reconstruction efficiency measurements. Section 12 concludes with a summary of the results.

## The ATLAS detector

A complete description of the ATLAS detector is provided in Ref. [1]. A brief description of the subdetectors that are relevant for the detection of electrons is given in this section.

The ID provides precise reconstruction of tracks within $|\eta | < 2.5$. It consists of three layers of pixel detectors close to the beam-pipe, four layers of silicon microstrip detector modules with pairs of single-sided sensors glued back-to-back (SCT) providing eight hits per track at intermediate radii, and a transition radiation tracker (TRT) at the outer radii, providing on average 35 hits per track in the range $|\eta | < 2.0$. The TRT offers substantial discriminating power between electrons and charged hadrons between energies of 0.5 and 100 $\text {GeV}$, via the detection of X-rays produced by transition radiation. The innermost pixel layer in 2012 and earlier, also called the b-layer, is located outside the beam-pipe at a radius of 50 mm. Together with the other layers, it provides precise vertexing and significant rejection of photon conversions through the requirement that a track has a hit in this layer.

The ID is surrounded by a thin superconducting solenoid with a length of 5.3 m and diameter of 2.5 m. The solenoid provides a 2 T magnetic field for the measurement of the curvature of charged particles to determine their charge and momentum. The solenoid design attempts to minimize the amount of material by integrating it into a vacuum vessel shared with the LAr calorimeter. The magnet thus only contributes a total of 0.66 radiation lengths of material at normal incidence.

The main EM calorimeter is a lead/liquid-argon sampling calorimeter with accordion-shaped electrodes and lead absorber plates. It is divided into a barrel section (EMB) covering $|\eta |<1.475$ and two endcap sections (EMEC) covering $1.375<|\eta |<3.2$. For $|\eta |<2.5$, it is divided into three layers longitudinal in shower depth (called strip, middle and back layers) and offers a fine segmentation in the lateral direction of the showers. At high energy, most of the EM shower energy is collected in the middle layer which has a lateral granularity of 0.025 $\times$ 0.025 in $\eta$–$\phi$ space. The first (strip) layer consists of strips with a finer granularity in the $\eta$-direction and with a coarser granularity in $\phi$. It provides excellent $\gamma$–$\pi ^0$ discrimination and a precise estimation of the pseudorapidity of the impact point. The back layer collects the energy deposited in the tail of high-energy EM showers. A thin presampler detector, covering $|\eta |<1.8$, is used to correct for fluctuations in upstream energy losses. The transition region between the EMB and EMEC calorimeters, $1.37<|\eta |<1.52$, suffers from a large amount of material.

Hadronic calorimeters with at least three segments longitudinal in shower depth surround the EM calorimeter and are used in this context to reject hadronic jets. The forward calorimeters cover the range $3.1< |\eta | < 4.9$ and also have EM shower identification capabilities given their fine lateral granularity and longitudinal segmentation into three layers.

## Electron reconstruction

Electron reconstruction in the central region of the ATLAS detector ($|\eta |<2.47$) starts from energy deposits (clusters) in the EM calorimeter which are then matched to reconstructed tracks of charged particles in the ID.

### Electron seed-cluster reconstruction

The $\eta$–$\phi$ space of the EM calorimeter system is divided into a grid of $N_\eta \times N_\phi = 200 \times 256$ towers of size $\Delta \eta ^\mathrm {tower} \times \Delta \phi ^\mathrm {tower} = 0.025 \times 0.025$, corresponding to the granularity of the EM accordion calorimeter middle layer. The energy of the calorimeter cells in all shower-depth layers (the strip, middle and back EM accordion calorimeter layers and for $|\eta |<1.8$ also the presampler detector) is summed to get the tower energy. The energy of a cell which spans several towers is distributed evenly among the towers without taking into account any geometrical weighting.

To reconstruct the EM clusters, seed clusters of towers with total cluster transverse energy above 2.5 $\text {GeV}$ are searched for by a sliding-window algorithm [3]. The window size is $3 \times 5$ towers in $\eta$–$\phi$ space. A duplicate-removal algorithm is applied to nearby seed clusters.

Cluster reconstruction is expected to be very efficient for true electrons. In MC samples passing the full ATLAS simulation chain, the efficiency is about 95% for electrons with a transverse energy of $E_{\mathrm {T}}=7$ $\text {GeV}$ and reaches 99% at $E_{\mathrm {T}}=15$ $\text {GeV}$ and 99.9% at $E_{\mathrm {T}}=45$ $\text {GeV}$, placing a requirement only on the angular distance between the generated electron and the reconstructed electron cluster. The efficiency decreases with increasing pseudorapidity in the endcap region $|\eta |>1.37$.

### Electron-track candidate reconstruction

Track reconstruction for electrons was improved for the 2012 data-taking period with respect to the one used for 2011 data-taking, especially for electrons which undergo significant energy loss due to bremsstrahlung in the detector, to achieve a high and uniform efficiency.

Table 1 shows the definition of shower-shape and track-quality variables, including $R_\eta$ and $R_\mathrm {Had}$. For each seed EM cluster^2^ passing loose shower-shape requirements of $R_\eta > 0.65$ and $R_\mathrm {Had} < 0.1$ a region-of-interest (ROI) is defined as a cone of size $\Delta R$ = 0.3 around the seed cluster barycentre. The collection of these EM cluster ROIs is retained for use in the track reconstruction.

Track reconstruction proceeds in two steps: pattern recognition and track fit. In 2012, in addition to the standard track-pattern recognition and track fit, an electron-specific pattern recognition and track fit were introduced in order to recover losses from bremsstrahlung and therefore improve the reconstruction of electrons. Either of these algorithms, the pattern recognition and the track fit, use a particle-specific hypothesis for the particle mass and respective probability for the particle to undergo bremsstrahlung, referred to in the following either as pion or electron hypothesis.

The standard pattern recognition [4] uses the pion hypothesis for energy loss in the material of the detector. If a track^3^ seed (consisting of three hits in different layers of the silicon detectors) with a transverse momentum larger than 1 $\text {GeV}$ cannot be successfully extended to a full track with at least seven hits using the pion hypothesis and it falls within one of the EM cluster ROIs, it is retried with the new pattern recognition using an electron hypothesis that allows for energy loss. This modified pattern recognition algorithm (based on a Kalman filter–smoother formalism [5]) allows up to 30% energy loss at each material surface to account for bremsstrahlung. Below 1 $\text {GeV}$, no refitting is performed. Thus, an electron-specific algorithm has been integrated into the standard track reconstruction; it improves the performance for electrons and has minimal interference with the main track reconstruction.

Track candidates are fitted using either the pion or the electron hypothesis (according to the hypothesis used in the pattern recognition) with the ATLAS Global $\chi ^2$ Track Fitter [6]. The electron hypothesis employs the same track fit as for the pion hypothesis except that it adds an extra term to compensate for the increase in $\chi ^2$ due to bremsstrahlung losses, in order to be able to fit the track with an acceptable $\chi ^2$ such that it can be further used in the electron reconstruction. If a track candidate fails the pion hypothesis track fit due to a large $\chi ^2$ (for example caused by large energy losses), it is refitted using the electron hypothesis.

Tracks are then considered as loosely matched to an EM cluster, if they pass either of the following two requirements:

(i) Tracks with at least four silicon hits are extrapolated from their measured perigee to the middle layer of the EM accordion calorimeter. In the middle layer of the calorimeter, the extrapolated tracks have to be either within 0.2 in $\phi$ of the EM cluster on the side the track is bending towards or within 0.05 on the opposite side. They also have to be within 0.05 in $\eta$ of the EM cluster. TRT-only tracks, i.e. tracks with less than four silicon hits, are extrapolated from the last measurement point. They are retained at this early stage as they are used later in the reconstruction chain to reconstruct photon conversions. Clusters without any associated tracks with silicon hits are eventually considered as photons and are not used to reconstruct prompt-electron candidates. TRT-only tracks have to pass the same requirement for the difference in $\phi$ between track and cluster as tracks with silicon hits but no requirement is placed on the difference in $\eta$ between track and cluster at this stage as their $\eta$ coordinate is not measured precisely.
(ii) The track extrapolated to the middle layer of the EM accordion calorimeter, after rescaling its momentum to the measured cluster energy, has to be either within 0.1 in $\phi$ of the EM cluster on the side the track is bending towards or within 0.05 on the opposite side. Furthermore, non-TRT-only tracks must be within 0.05 in $\eta$ of the calorimeter cluster. As in (i), the track extrapolation is made from the last measurement point for TRT-only tracks and from the point of closest approach with respect to the primary collision vertex for tracks with silicon hits.

Criterion (ii) aims to recover tracks of typically large curvature that have potentially suffered significant energy loss before reaching the calorimeter. Rescaling the momentum of the track to that of the reconstructed cluster allows retention of tracks whose measured momentum in the ID does not match the energy reconstructed in the calorimeter because they have undergone bremsstrahlung. The bremsstrahlung is assumed to have occurred in the ID or the cryostat and solenoid before the calorimeter (for tracks with silicon hits) or in the cryostat and solenoid before the calorimeter (for TRT-only tracks).

At this point, all electron-track candidates are defined. The track parameters of these candidates, for all but the TRT-only tracks, are precisely re-estimated using an optimized electron track fitter, the Gaussian Sum Filter (GSF) [7] algorithm, which is a non-linear generalization of the Kalman filter [5] algorithm. It yields a better estimate of the electron track parameters, especially those in the transverse plane, by accounting for non-linear bremsstrahlung effects. TRT-only tracks and the very rare tracks (about 0.01%) that fail the GSF fit keep the parameters from the Global $\chi ^2$ Track Fit. These tracks are then used to perform the final track-cluster matching to build electron candidates and also to provide information for particle identification.

### Electron-candidate reconstruction

An electron is reconstructed if at least one track is matched to the seed cluster. The efficiency of this matching and subsequent track quality requirements is measured as the reconstruction efficiency in Sect. 10. The track-cluster matching proceeds as described for the previous step in Sect. 3.2, but with the GSF refitted tracks and tighter requirements: the separation in $\phi$ must be less than 0.1 (and not 0.2). Additionally, TRT-only tracks must satisfy loose track-cluster matching criteria in $\eta$ and tighter ones in $\phi$: in the TRT barrel $|\Delta \eta | < 0.35$ and in the TRT endcap $|\Delta \eta | < 0.02$. In both the barrel and the endcaps the requirements are $|\Delta \phi | < 0.03$ on the side the track is bending towards and $|\Delta \phi | < 0.02$ on the other side. In this procedure, more than one track can be associated with a cluster.

Although all tracks assigned to a cluster are kept for further analysis, the best-matched one is chosen as the primary track which is used to determine the kinematics and charge of the electron and to calculate the electron identification decision. Thus choosing the primary track is a crucial step in the electron reconstruction chain. To favour the primary electron track and to avoid random matches between nearby tracks in the case of cascades due to bremsstrahlung, tracks with at least one hit in the pixel detector are preferred. If more than one associated track has pixel hits, the following sorting criteria are considered. First, the distance between the track and the cluster is considered for any pair of tracks, which are referred to as *i* and *j* in the following. Then two angular distance variables are defined in the $\eta$–$\phi$ plane. $\Delta R$ is the distance between the cluster barycentre and the extrapolated track in the middle layer of the EM accordion calorimeter, while $\Delta R_\mathrm {rescaled}$ is the distance between the cluster barycentre and the extrapolated track when the track momentum is rescaled to the measured cluster energy before the extrapolation to the middle layer. If $|\Delta R_{\mathrm {rescaled},i} - \Delta R_{\mathrm {rescaled},j}| > 0.01$, the track with the smaller $\Delta R_\mathrm {rescaled}$ is chosen. If $| \Delta R_{\mathrm {rescaled},i} - \Delta R_{\mathrm {rescaled},j}| \le 0.01$ and $|\Delta R_i - \Delta R_j| > 0.01$, the track with smaller $\Delta R$ is taken. For the rest of the cases, the two tracks have both similar $\Delta R_\mathrm {rescaled}$ and similar $\Delta R$, and the track with more pixel hits^4^ is chosen as the primary track. A hit in the first layer of the pixel detector counts twice to prefer tracks with early hits. If there are two best tracks with exactly the same numbers of hits, the track with smaller $\Delta R$ is taken.

All seed clusters together with their matching tracks, if there is at least one of them, are treated as electron candidates. Each of these electron clusters is then rebuilt in all four layers sequentially, starting from the middle layer, using $3 \times 7$ ($5 \times 5$) cells in $\eta \times \phi$ in the barrel (endcaps) of the EM accordion calorimeter. The cluster position is adjusted in each layer to take into account the distribution of the deposited energy. The fixed sizes of $3 \times 7$ ($5 \times 5$) cells for electron clusters were optimized to take into account the different overall energy distributions in the barrel (endcap) accordion calorimeters specifically for electrons.^5^

Up to this point, neither the electron clusters nor the cells inside the clusters are calibrated. The energy calibration [8] is applied as the next step and was improved for 2012 data using multivariate analysis (MVA) techniques [9] and an improved description of the detector [10] by the GEANT4 [11] simulation. The calibration procedure is outlined briefly below.

After applying the electronic readout calibration to the calorimeter cells with a global energy scale factor corresponding to the electron response, a number of data pre-corrections are applied for measured effects of the bunch train structure and imperfectly corrected response in specific regions. The presampler energy scales and the EM accordion calorimeter strip-to-middle-layer energy-scale ratios are also corrected [8].

The cluster energy is then determined from the energy in the three layers of the EM accordion calorimeter by applying a correction factor determined by linear regression using an MVA trained on large samples of single-electron MC events produced with the full ATLAS simulation chain. The input quantities used for electrons and photons are the total energy measured in the accordion calorimeter, the ratio of the energy measured in the presampler to the energy measured in the accordion, the shower depth,^6^ the pseudorapidity of the cluster barycentre in the ATLAS coordinate system, and the $\eta$ and $\phi$ positions of the cluster barycentre in the local coordinate system of the calorimeter. Including the cluster barycentre position allows a correction to be made for the larger lateral energy leakage for particles that hit a cell close to the edge and for the variation of the response as a function of the particle impact point with respect to the calorimeter absorbers.

In the last step, correction factors are derived in situ using a large sample of collected $Z \rightarrow ee$ events. They are applied to the reconstructed electrons as a final energy calibration in data events. Electron energies are smeared in simulated events, as the simulated electrons have a better energy resolution than electrons in data.

The four-momentum of central electrons ($|\eta |<2.47$) is computed using information from both the final cluster and the track best matched to the original seed cluster. The energy is given by the cluster energy. The $\phi$ and $\eta$ directions are taken from the corresponding track parameters, except for TRT-only tracks for which the cluster $\phi$ and $\eta$ values are used.

## Electron identification

Not all objects built by the electron reconstruction algorithms are prompt electrons which are considered signal objects in this publication. Background objects include hadronic jets as well as electrons from photon conversions, Dalitz decays and from semileptonic heavy-flavour hadron decays. In order to reject as much of these backgrounds as possible while keeping the efficiency for prompt electrons high, electron identification algorithms are based on discriminating variables, which are combined into a menu of selections with various background rejection powers. Sequential requirements and MVA techniques are employed.

Variables describing the longitudinal and lateral shapes of the EM showers in the calorimeters, the properties of the tracks in the ID, as well as the matching between tracks and energy clusters are used to discriminate against the different background sources. These variables [2, 12, 13] are detailed in Table 1. Table 2 summarizes which variables are used for the different selections of the so-called cut-based and likelihood (LH) [14] identification menus.

**Table 1:** Definition of electron discriminating variables

Type	Description	Name
Hadronic leakage	Ratio of $E_{\mathrm {T}}$ in the first layer of the hadronic calorimeter to $E_{\mathrm {T}}$ of the EM cluster (used over the range $|\eta | < 0.8$ or $|\eta | > 1.37$)	$R_\mathrm {Had1}$
	Ratio of $E_{\mathrm {T}}$ in the hadronic calorimeter to $E_{\mathrm {T}}$ of the EM cluster (used over the range $0.8<|\eta | < 1.37$)	$R_\mathrm {Had}$
Back layer of EM calorimeter	Ratio of the energy in the back layer to the total energy in the EM accordion calorimeter	$f_3$
Middle layer of EM calorimeter	Lateral shower width, $\sqrt{(\Sigma E_i \eta _i^2)/(\Sigma E_i) -((\Sigma E_i\eta _i)/(\Sigma E_i))^2}$, where $E_i$ is the energy and $\eta _i$ is the pseudorapidity of cell *i* and the sum is calculated within a window of $3 \times 5$ cells	$w_{\eta 2}$
	Ratio of the energy in 3 $\times$ 3 cells to the energy in 3 $\times$ 7 cells centred at the electron cluster position	$R_\phi$
	Ratio of the energy in 3 $\times$ 7 cells to the energy in 7 $\times$ 7 cells centred at the electron cluster position	$R_\eta$
Strip layer of EM calorimeter	Shower width, $\sqrt{(\Sigma E_i (i-i_\mathrm {max})^2)/(\Sigma E_i)}$, where *i* runs over all strips in a window of $\Delta \eta \times \Delta \phi \approx 0.0625 \times 0.2$, corresponding typically to 20 strips in $\eta$, and $i_\mathrm {max}$ is the index of the highest-energy strip	${ w}_\mathrm{stot}$
	Ratio of the energy difference between the maximum energy deposit and the energy deposit in a secondary maximum in the cluster to the sum of these energies	$E_\text {ratio}$
	Ratio of the energy in the strip layer to the total energy in the EM accordion calorimeter	$f_1$
Track quality	Number of hits in the b-layer (discriminates against photon conversions)	$n_\mathrm {Blayer}$
	Number of hits in the pixel detector	$n_\mathrm {Pixel}$
	Total number of hits in the pixel and SCT detectors	$n_{\mathrm {Si}}$
	Transverse impact parameter	$d_0$
	Significance of transverse impact parameter defined as the ratio of the magnitude of $d_0$ to its uncertainty	$\sigma _{d_0}$
	Momentum lost by the track between the perigee and the last measurement point divided by the original momentum	$\Delta p/p$
TRT	Total number of hits in the TRT	$n_\mathrm {TRT}$
	Ratio of the number of high-threshold hits to the total number of hits in the TRT	$F_\text {HT}$
Track-cluster matching	$\Delta \eta$ between the cluster position in the strip layer and the extrapolated track	$\Delta \eta$
	$\Delta \phi$ between the cluster position in the middle layer and the extrapolated track	$\Delta \phi$
	Defined as $\Delta \phi$, but the track momentum is rescaled to the cluster energy before extrapolating the track to the middle layer of the calorimeter	$\Delta \phi _\text {res}$
	Ratio of the cluster energy to the track momentum	*E* / *p*
Conversions	Veto electron candidates matched to reconstructed photon conversions	isConv

**Table 2:** The variables used in the different selections of the electron identification menu

	Cut-based				Likelihood		
Name	Loose	Medium	Tight	Multilepton	Loose LH	Medium LH	Very Tight LH
$R_\mathrm {Had(1)}$	$\checkmark$	$\checkmark$	$\checkmark$	$\checkmark$	$\checkmark$	$\checkmark$	$\checkmark$
$f_3$		$\checkmark$	$\checkmark$	$\checkmark$	$\checkmark$	$\checkmark$	$\checkmark$
$w_{\eta 2}$	$\checkmark$	$\checkmark$	$\checkmark$	$\checkmark$	$\checkmark$	$\checkmark$	$\checkmark$
$R_\eta$	$\checkmark$	$\checkmark$	$\checkmark$	$\checkmark$	$\checkmark$	$\checkmark$	$\checkmark$
$R_\phi$					$\checkmark$	$\checkmark$	$\checkmark$
${ w}_\mathrm{stot}$	$\checkmark$	$\checkmark$	$\checkmark$	$\checkmark$			
$E_\text {ratio}$	$\checkmark$	$\checkmark$	$\checkmark$	$\checkmark$	$\checkmark$	$\checkmark$	$\checkmark$
$f_1$					$\checkmark$	$\checkmark$	$\checkmark$
$n_\mathrm {Blayer}$		$\checkmark$	$\checkmark$	$\checkmark$	$\checkmark$	$\checkmark$	$\checkmark$
$n_\mathrm {Pixel}$	$\checkmark$	$\checkmark$	$\checkmark$	$\checkmark$	$\checkmark$	$\checkmark$	$\checkmark$
$n_\mathrm {Si}$	$\checkmark$	$\checkmark$	$\checkmark$	$\checkmark$	$\checkmark$	$\checkmark$	$\checkmark$
$d_0$		$\checkmark$	$\checkmark$			$\checkmark$	$\checkmark$
$\sigma _{d_0}$						$\checkmark$	$\checkmark$
$\Delta p/p$				$\checkmark$	$\checkmark$	$\checkmark$	$\checkmark$
$n_\mathrm {TRT}$		$\checkmark$	$\checkmark$	$\checkmark$			
$F_\text {HT}$		$\checkmark$	$\checkmark$	$\checkmark$	$\checkmark$	$\checkmark$	$\checkmark$
$\Delta \eta$	$\checkmark$	$\checkmark$	$\checkmark$	$\checkmark$	$\checkmark$	$\checkmark$	$\checkmark$
$\Delta \phi$			$\checkmark$				
$\Delta \phi _\text {res}$				$\checkmark$	$\checkmark$	$\checkmark$	$\checkmark$
*E* / *p*			$\checkmark$				
isConv			$\checkmark$				$\checkmark$

### Cut-based identification

The cut-based selections, Loose, Medium, Tight and Multilepton, are optimized in 10 bins in $|\eta |$ and 11 bins in $E_{\mathrm {T}}$. This binning allows the identification to take into account the variation of the electrons’ characteristics due to e.g. the dependence of the shower shapes on the amount of passive material traversed before entering the EM calorimeter. Shower shapes and track properties also change with the energy of the particle. The electrons selected with Tight are a subset of the electrons selected with Medium, which in turn are a subset of Loose electrons. With increasing tightness, more variables are added and requirements are tightened on the variables already used in the looser selections.

Due to its simplicity, the cut-based electron identification [2, 12, 13], which is based on sequential requirements on selected variables, has been used by the ATLAS Collaboration for identifying electrons since the beginning of data-taking. In 2011, for $\sqrt{s} = 7$ $\text {TeV}$ collisions, its performance (defined in terms of efficiency and background rejection) was improved by loosening requirements and introducing additional variables, especially in the looser selections [2]. In 2012, for $\sqrt{s} = 8$ $\text {TeV}$ collisions, due to higher instantaneous luminosities provided by the LHC, the number of overlapping collisions (pile-up) and therefore the number of particles in an event^7^ increased. Due to the higher energy density per event, the shower shapes, even of isolated electrons, tend to look more background-like. In order to cope with this, requirements were loosened on the variables most sensitive to pile-up ($R_\mathrm {Had(1)}$ and $R_\eta$) and tightened on others to keep the performance (efficiency/background rejection) roughly constant as a function of the number of reconstructed primary vertices. A requirement on $f_3$ was added in 2012, as well. Furthermore, a new selection was added, called Multilepton, which is optimized for the low-energy electrons in the $H \rightarrow ZZ^* \rightarrow 4\ell$ ($\ell = e, \mu$) analysis. For these electrons, Multilepton has a similar efficiency to the Loose selection, but provides a better background rejection. In comparison to Loose, requirements on the shower shapes are loosened and more variables are added, including those sensitive to bremsstrahlung effects.

### Likelihood identification

MVA techniques are powerful, since they allow the combined evaluation of several properties when making a selection decision. Out of the different MVA techniques, the LH was chosen for electron identification because of its simple construction.

The electron LH makes use of signal and background probability density functions (pdfs) of the discriminating variables. Based on these pdfs, which are treated as uncorrelated, an overall probability is calculated for the object to be signal or background. The signal and background probabilities for a given electron candidate are combined into a discriminant $d_{\mathcal L}$:

1 $$\begin{aligned} d_{\mathcal L} = {\mathcal L_\mathrm{S} \over \mathcal L_\mathrm{S} + \mathcal L_\mathrm{B} },\quad \mathcal L_\mathrm{S(B)}(\vec {x}) = \prod \limits _{i=1}^{n} P_{\mathrm{S(B)},i}(x_i) \end{aligned}$$

where $\vec {x}$ is the vector of variable values and $P_{\mathrm{S},i}(x_i)$ is the value of the signal probability density function of the $i\text {th}$ variable evaluated at $x_i$. In the same way, $P_{\mathrm{B},i}(x_i)$ refers to the background probability density function.

Signal and background pdfs used for the electron LH identification are obtained from data. As in the Multilepton cut-based selection, variables sensitive to bremsstrahlung effects are included.

Furthermore, additional variables with significant discriminating power but also a large overlap between signal and background that prevents explicit requirements (like $R_\phi$ and $f_1$) are included. The variables counting the hits on the track are not used as pdfs in the LH, but are left as simple requirements, as every electron should have a high-quality track to allow a robust momentum measurement.

The Loose LH, Medium LH, and Very Tight LH selections are designed to roughly match the electron efficiencies of the Multilepton, Medium and Tight cut-based selections, but to have better rejection of light-flavour jets and conversions.^8^

Each LH selection places a requirement on a LH discriminant, made with a different set of variables. The Loose LH features variables most useful for discrimination against light-flavour jets (in addition, a requirement on $n_\mathrm {Blayer}$ is applied to reject conversions). In the Medium LH and Very Tight LH regimes, additional variables ($d_0$, isConv) are added for further rejection of heavy-flavour jets and conversions. Although different variables are used for the different selections, a sample of electrons selected using a tighter LH is a subset of the electron samples selected using the looser LH to a very good approximation.

The LH for each selection consists of 9 $\times$ 6 sets of pdfs, divided into 9 $|\eta |$ bins and 6 $E_{\mathrm {T}}$ bins. This binning is similar to, but coarser than, the binning used for the cut-based selections. It is chosen to balance the available number of events with the variation of the pdf shapes in $E_{\mathrm {T}}$ and $|\eta |$.

### Electron isolation

In order to further reject hadronic jets misidentified as electrons, most analyses require electrons to pass some isolation requirement in addition to the identification requirements described above. The two main isolation variables are:

- Calorimeter-based isolation:

The calorimetric isolation variable $E_\mathrm {T}^{\mathrm {cone} \Delta R}$ is defined as the sum of the transverse energy deposited in the calorimeter cells in a cone of size $\Delta R$ around the electron, excluding the contribution within $\Delta \eta \times \Delta \phi = 0.125 \times 0.175$ around the electron cluster barycentre. It is corrected for energy leakage from the electron shower into the isolation cone and for the effect of pile-up using a correction parameterized as a function of the number of reconstructed primary vertices.

- Track-based isolation:

The track isolation variable $p_\mathrm {T}^{\mathrm {cone} \Delta R}$ is the scalar sum of the transverse momentum of the tracks with $p_{\mathrm {T}}$ > 0.4 $\text {GeV}$ in a cone of $\Delta R$ around the electron, excluding the track of the electron itself. The tracks considered in the sum must originate from the primary vertex associated with the electron track and be of good quality; i.e. they must have at least nine silicon hits, one of which must be in the innermost pixel layer.

Both types of isolation are used in the tag-and-probe measurements, mainly in order to tighten the selection criteria of the tag. Whenever isolation is applied to the probe electron candidate in this work (this only happens in the $J/\psi$ analysis described in Sect. 7.2), the criteria are chosen such that the effect on the measured identification efficiency is estimated to be small.

## Efficiency measurement methodology

### The tag-and-probe method

Measuring the identification and reconstruction efficiency requires a clean and unbiased sample of electrons. The method of choice is the tag-and-probe method, which makes use of the characteristic signatures of $Z \rightarrow ee$ and $J/\psi \rightarrow ee$ decays. In both cases, strict selection criteria are applied on one of the two decay electrons, called tag, and the second electron, the probe, is used for the efficiency measurements. Additional event selection criteria are applied to further reject background. Only events satisfying data-quality criteria, in particular concerning the ID and the calorimeters, are considered. Furthermore, at least one reconstructed primary vertex with at least three tracks must be present in the event. The tag-and-probe pairs must also pass requirements on their reconstructed invariant mass. In order to not bias the selected probe sample, each valid combination of electron pairs in the event is considered; an electron can be the tag in one pair and the probe in another.

The probe samples are contaminated by background objects (for example, hadrons misidentified as electrons, electrons from semileptonic heavy flavour decays or from photon conversions). This contamination is estimated using either background template shapes or combined fits of background and signal analytical models to the data. The number of electrons is independently estimated at the probe level and at the level where the probe electron candidate satisfies the tested criteria. The efficiency $\epsilon$ is defined as the fraction of probe electrons satisfying the tested criteria.

The efficiency to detect an electron is divided into different components, namely trigger, reconstruction and identification efficiencies, as well as the efficiency to satisfy additional analysis criteria, like isolation. The full efficiency $\epsilon _{\mathrm {total}}$ for a single electron can be written as:

2 $$\begin{aligned} \epsilon _{\mathrm{total}}&= \epsilon _{\mathrm{reconstruction}}\times \epsilon _{\mathrm{identification}} \times \epsilon _{\mathrm{trigger}}\times \epsilon _{\mathrm{additional}} \nonumber \\&= \frac{N_\mathrm{reconstruction}}{N_\mathrm{clusters}} \times \frac{N_\mathrm{identification}}{N_\mathrm{reconstruction}} \times \frac{N_\mathrm{trigger}}{N_\mathrm{identification}} \times \frac{N_\mathrm{additional}}{N_\mathrm{trigger}}. \end{aligned}$$

The efficiency components are defined and measured in a specific order to preserve consistency: the reconstruction efficiency, $\epsilon _{\mathrm {reconstruction}}$, is measured with respect to electron clusters reconstructed in the EM calorimeter $N_\mathrm {clusters}$; the identification efficiency $\epsilon _{\mathrm {identification}}$ is determined with respect to reconstructed electrons $N_\mathrm {reconstruction}$. Trigger efficiencies are calculated for reconstructed electrons satisfying a given identification criterion $N_\mathrm {identification}$. Therefore, for each identification selection a dedicated set of trigger efficiency measurements is performed. Additional selection criteria are often imposed in analyses of collision data, for example on the isolation of electrons (introduced in Sect. 4.3). Neither trigger nor isolation efficiency measurements are covered here.

The determination of $\epsilon _{\mathrm {identification}}$ and $\epsilon _{\mathrm {reconstruction}}$ is described in Sects. 7 and 10. The efficiencies are measured in data and in simulated $Z \rightarrow ee$ and $J/\psi \rightarrow ee$ samples. To compare the data values with the estimates of the MC simulation, the same requirements are used to select the probe electrons. However, no background needs to be subtracted from the simulated samples; instead, the reconstructed electron track must be matched to an electron trajectory provided by the MC simulation within $\Delta R < 0.2$. Matched electrons from converted photons that are radiated off an electron originating from a *Z* or $J/\psi$ decay are also accepted by the analyses. The denominator of the reconstruction efficiency includes electrons that were not properly reconstructed. If electrons in the simulated $Z \rightarrow ee$ samples are reconstructed as clusters without a matching track, the *Z* decay electrons provided by the MC simulation are matched to the reconstructed cluster within $\Delta R < 0.2$.

#### Data-to-MC correction factors

The accuracy with which the MC detector simulation models the electron efficiency plays an important role in cross-section measurements and various searches for new physics. In order to achieve reliable results, the simulated MC samples need to be corrected to reproduce the measured data efficiencies as closely as possible. This is achieved by a multiplicative correction factor defined as the ratio of the efficiency measured in data to that in the simulation. These data-to-MC correction factors are usually close to unity. Deviations come from the mismodelling of tracking properties or shower shapes in the calorimeters.

Since the electron efficiencies depend on the transverse energy and pseudorapidity, the measurements are performed in two-dimensional bins in ($E_{\mathrm {T}}$, $\eta$). These bins follow the detector geometry and the binning used for optimization and are as narrow as the size of the respective data set allows. Residual effects, due to the finite bin widths and kinematic differences of the physics processes used in the measurements, are expected to cancel in the data-to-MC efficiency ratio. Therefore, the combination of the different efficiency measurements is carried out using the data-to-MC ratios instead of the efficiencies themselves. The procedure for the combination is described in Sect. 7.3.

### Determination of central values and uncertainties

For the evaluation of the results of the measurements and their uncertainties using a given final state ($Z \rightarrow ee$, $Z \rightarrow ee\gamma$ or $J/\psi \rightarrow ee$), the following approach was chosen. The details of the efficiency measurement methods are varied in order to estimate the impact of the analysis choices and potential imperfections in the background modelling. Examples of these variations are the selection of the tag electron or the background estimation method. For the measurement of the data-to-MC correction factors, the same variations of the selection are applied consistently in data and MC simulation. Uncertainties due to charge misidentification of the tag-and-probe pairs are neglected.

The final result (the central value) of a given efficiency measurement using one of the $Z \rightarrow ee$, $Z \rightarrow ee\gamma$ or $J/\psi \rightarrow ee$ processes is taken to be the average value of the results from all variations (including the use of different background subtraction methods, e.g. $Z_{\mathrm {iso}}$ and $Z_{\mathrm {mass}}$ for the $Z \rightarrow ee$ final state as described in Sects. 7.1.2, 7.1.3).

The systematic uncertainty is estimated to be equal to the root mean square (RMS) of the measurements with the intention of modelling a 68% confidence interval. However, in many bins the RMS does not cover at least 68% of all the variations, so an empirical factor of 1.2 is applied to the determined uncertainty in all bins.

The statistical uncertainty is taken to be the average of the statistical uncertainties over all investigated variations of the measurement. The statistical uncertainty in a single variation of the measurement is calculated following the approach in Ref. [15].

## Data and Monte Carlo samples

The results in this paper are based on 8 $\text {TeV}$ LHC *pp* collision data collected with the ATLAS detector in 2012. After requiring good data quality, in particular concerning the ID and the EM and hadronic calorimeters, the integrated luminosity used for the measurements is 20.3 fb$^{-1}$.

The measurements are compared to predictions from MC simulation. The $Z \rightarrow ee$ and $Z \rightarrow ee\gamma$ MC samples are generated with the POWHEG-BOX [16–18] generator interfaced to PYTHIA8 [19], using the CT10 NLO PDF set [20] for the hard process, the CTEQ6L1 PDF set [21] and a set of tuned parameters called the AU2CT10 showering tune [22] for the parton shower. The $J/\psi \rightarrow ee$ events are simulated using PYTHIA8 both for prompt ($pp \rightarrow J/\psi +X$) and for non-prompt ($b\bar{b} \rightarrow J/\psi +X$) production. The CTEQ6L1 LO PDF set is used, as well as the AU2CTEQ6L1 parameter set for the showering [22]. All MC samples are processed through the full ATLAS detector simulation [10] based on GEANT4 [11].

**Fig. 1:**
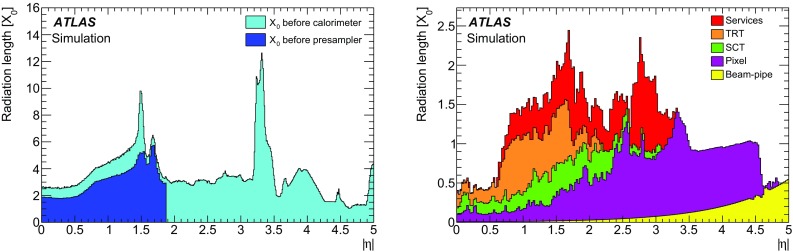
Amount of material, in units of radiation length $X_{0}$, traversed by a particle as a function of $|\eta |$: material in front of the presampler detector and the EM accordion calorimeter (*left*), and material up to the ID boundaries (*right*). The contributions of the different detector elements, including the services and thermal enclosures are shown separately by *filled colour areas*

The distribution of material in front of the presampler detector and the EM accordion calorimeter as a function of $|\eta |$ is shown in the left plot of Fig. 1. The contributions of the different detector elements up to the ID boundaries, including services and thermal enclosures, are detailed on the right. These material distributions are used as input to the MC simulation.

The peak at $|\eta | \approx 1.5$ in the left plot of Fig. 1, corresponding to the transition region between the barrel and endcap EM accordion calorimeters, is due to the cryostats, the corner of the barrel EM accordion calorimeter, the ID services and parts of the scintillator-tile hadronic calorimeter. The sudden increase of material at $|\eta | \approx 3.2$, corresponding to the transition between the endcap calorimeters and the forward calorimeter, is mostly due to the cryostat that acts also as a support structure.

**Fig. 2:**
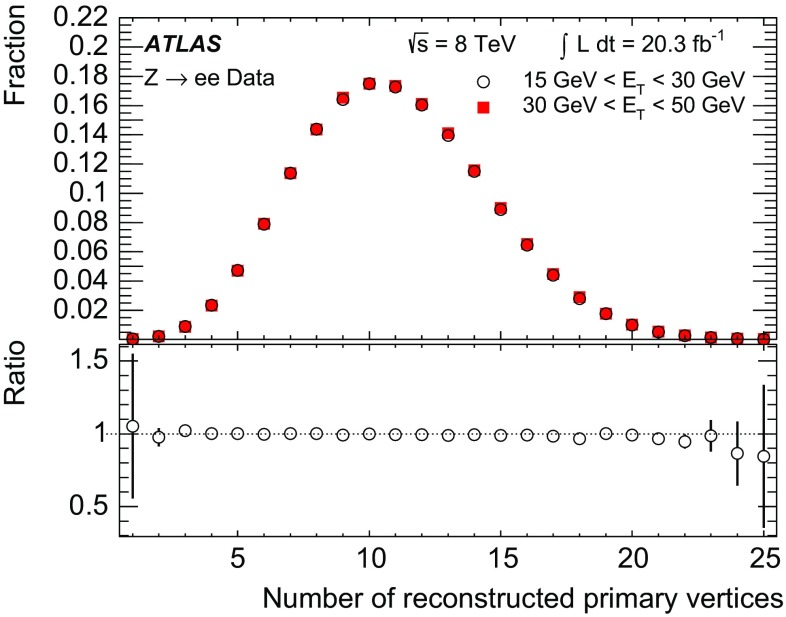
Number of reconstructed primary vertices in events with an electron cluster candidate with 15 $\text {GeV}$ < $E_{\mathrm {T}}$ < 30 $\text {GeV}$ (*open circles*) and 30 $\text {GeV}$ < $E_{\mathrm {T}}$ < 50 $\text {GeV}$ (*filled squares*) in the $Z \rightarrow ee$ data set used for the reconstruction efficiency measurement described in Sect. 10

The simulation also includes realistic modelling (tuned to the data) of the event pile-up from the same, previous, and subsequent bunch crossings. The energies of the electron candidates in simulation are smeared to match the resolution in data and the simulated MC events are weighted to reproduce the distributions of the primary-vertex *z*-position and the number of vertices in data, the latter being a good indicator of pile-up. Figure 2 shows the distribution of the number of primary collision vertices in events with an identified electron and an electron cluster candidate (with 15 $\text {GeV}$ < $E_{\mathrm {T}}$ < 30 $\text {GeV}$ and 30 $\text {GeV}$ < $E_{\mathrm {T}}$ < 50 $\text {GeV}$) in the $Z \rightarrow ee$ data set used for the reconstruction efficiency measurement described in Sect. 10. The distribution does not depend on the transverse energy of the cluster of the probe electron candidate.

## Identification efficiency measurement

The efficiencies of the identification criteria (Loose, Medium, Tight, Multilepton and Loose LH, Medium LH, Very Tight LH) are determined in data and in the simulated samples with respect to reconstructed electrons with associated tracks that have at least one hit in the pixel detector and at least seven total hits in the pixel and SCT detectors (this requirement is referred to as “track quality” below). The efficiencies are calculated as the ratio of the number of electrons passing a certain identification selection (numerator) to the number of electrons with a matching track satisfying the track quality requirements (denominator).

**Fig. 3:**
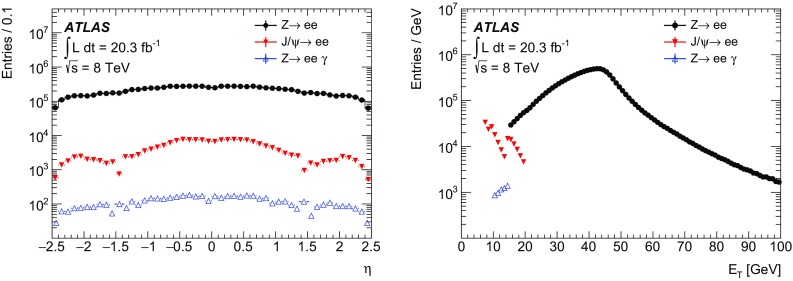
Pseudorapidity and transverse energy distributions of probe electron candidates satisfying the Tight identification criterion in the $Z \rightarrow ee$ (*full circles*), $Z \rightarrow ee\gamma$ (*empty triangles*) and the $J/\psi \rightarrow ee$ (*full triangles*) samples. The $E_{\mathrm {T}}$ distribution of probe electron candidates from $J/\psi \rightarrow ee$ is discontinuous, as the sample is selected by a number of triggers with different $E_{\mathrm {T}}$ thresholds

For the identification efficiencies determined in this paper, three different decays of resonances are used, and combined in the overlapping regions as described in Sect. 7.3: radiative decays of the *Z* boson, $Z \rightarrow ee\gamma$, for electrons with 10 $\text {GeV}$ < $E_{\mathrm {T}}$ < 15 $\text {GeV}$, $Z \rightarrow ee$ for electrons with $E_{\mathrm {T}}$ > 15 $\text {GeV}$ and $J/\psi \rightarrow ee$ for electrons with 7 $\text {GeV}$ < $E_{\mathrm {T}}$ < 20 $\text {GeV}$. The distributions of the probe electron candidates passing the Tight identification selection are depicted in Fig. 3 as a function of $\eta$ (left) and $E_{\mathrm {T}}$ (right), giving an indication of the number of events available for each of the measurements in the respective $\eta$ and $E_{\mathrm {T}}$ bin. The $E_{\mathrm {T}}$ spectrum of probe electron candidates from $J/\psi \rightarrow ee$ is discontinuous, as the sample is selected by a number of triggers with different $E_{\mathrm {T}}$ thresholds as discussed in Sect. 7.2.

### Tag-and-probe with $Z \rightarrow ee$ events

$Z \rightarrow ee$ $(\gamma )$ decays are used to measure the identification efficiency for electrons with $E_{\mathrm {T}}$ > 10 $\text {GeV}$. The tag-and-probe method using $Z \rightarrow ee$ decays provides a clean sample of electrons, especially when the probe electron candidates have $E_{\mathrm {T}}$ > 25 $\text {GeV}$. For lower transverse energies, background subtraction becomes important. Two different distributions are used to discriminate signal electrons from background: the invariant mass of the tag-and-probe pair is used in the $Z_{\mathrm {mass}}$ method and the isolation distribution of the probe electron candidate is used in the $Z_{\mathrm {iso}}$ method.

Probe electrons with $E_{\mathrm {T}}$ between 10 $\text {GeV}$ and 15 $\text {GeV}$ are selected from $Z \rightarrow ee$ $\gamma$ decays in which an electron has lost much of its energy due to final-state radiation (FSR). At low $E_{\mathrm {T}}$, this topology has less background than $Z \rightarrow ee$ decays. The invariant mass in these cases is computed from three objects: the tag electron, the probe electron and a photon.

#### Event selection

Events are selected using a logical OR between two single-electron triggers, one with an $E_{\mathrm {T}}$ threshold of 24 $\text {GeV}$ requiring medium identification and one with an $E_{\mathrm {T}}$ threshold of 60 $\text {GeV}$ and loose identification requirements.^9^

Events are required to have at least two reconstructed electron candidates in the central region of the detector, $|\eta |$ < 2.47, with opposite charges (see Sect. 9 for the measurement of the charge misidentification). The tag electron candidate is required to have a transverse energy $E_{\mathrm {T}}$ > 25 $\text {GeV}$, be matched to a trigger electron within $\Delta R$ < 0.15 and be outside the transition region between barrel and endcap of the EM calorimeter, 1.37 < $|\eta |$ < 1.52. Furthermore, it has to pass the Tight identification requirement (Medium for $Z \rightarrow ee\gamma$). The probe electron candidates must have $E_{\mathrm {T}}$ > 10 $\text {GeV}$ and satisfy the track quality criteria. The invariant mass of the tag–probe (tag–probe–photon for $Z \rightarrow ee\gamma$) system is required to be within ±15 $\text {GeV}$ of the $Z$ mass. About 15.5 million probe electron candidates are selected for further analysis.

For the $Z \rightarrow ee\gamma$ method, in addition to the tag and the probe electron candidates, a photon is selected passing Tight photon identification requirement [23] and fulfilling $E_{\mathrm {T}}$ (probe) + $E_{\mathrm {T}}$ (photon) > 30 $\text {GeV}$. Requirements are placed on the angular distance between the photon and the electron candidates to avoid double counting of objects: $\Delta R$ (tag–photon) > 0.4 and $\Delta R$ (probe–photon) > 0.2. The reason for the asymmetry between tag and probe electron requirements is an isolation requirement with a cone size of 0.4 which is applied to the tag electron as one of the variations for assessing the systematic uncertainties. Furthermore, FSR photons from the probe electron tend to be closer to the probe electron than to the tag electron. Further requirements are placed on the tag–probe and tag–photon invariant mass to select events with FSR: *m*(tag + photon) < 80 $\text {GeV}$, *m* (tag + probe) < 90 $\text {GeV}$. All possible tag–probe–photon combinations are used. About 13,000 probe electron candidates with a transverse energy of 10 $\text {GeV}$ < $E_{\mathrm {T}}$ < 15 $\text {GeV}$ are selected integrated over the full $|\eta |$ < 2.47 range.

#### Background estimation and variations for assessing the systematic uncertainties of the $Z_{\mathrm {mass}}$ method

The invariant mass of the tag-and-probe pair (and the photon in the case of $Z \rightarrow ee\gamma$) is used as the discriminating variable between signal electrons and background.

In order to form background templates, reconstructed electron candidates with an associated track, satisfying track quality criteria, are chosen as probes. In addition, identification and isolation requirements are inverted to minimize the contribution of signal electrons. A study was performed on data and simulated samples to test the shape biases of possible background templates due to the inversion of selection requirements and contamination from signal electrons, and the least-biased templates were chosen. The remaining signal electron contamination in the background templates is estimated using simulated events.

The normalization of the background template is determined by a sideband method: for the denominator (defined at the beginning of Sect. 7), the templates are normalized to the invariant-mass distribution above the $Z$ peak (120 $\text {GeV}$ < $m_{ee}$ < 250 $\text {GeV}$ for $Z \rightarrow ee$ and 100 $\text {GeV}$ < $m_{ee\gamma }$ < 250 $\text {GeV}$ for $Z \rightarrow ee\gamma$).

**Fig. 4:**
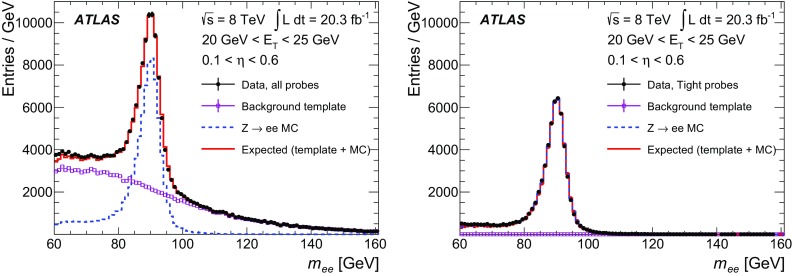
Illustration of the background estimation using the $Z_{\mathrm {mass}}$ method in the 20 $\text {GeV}$ < $E_{\mathrm {T}}$ < 25 $\text {GeV}$, 0.1 < $\eta$ < 0.6 bin, at reconstruction + track-quality level (*left*) and for probe electron candidates passing the cut-based Tight identification (*right*). The background template is normalized in the range 120 $\text {GeV}$ < $m_{ee}$ < 250 $\text {GeV}$. The tag electron passes cut-based Medium and isolation requirements. The signal MC simulation is scaled to match the estimated signal in the $Z$-mass window

**Fig. 5:**
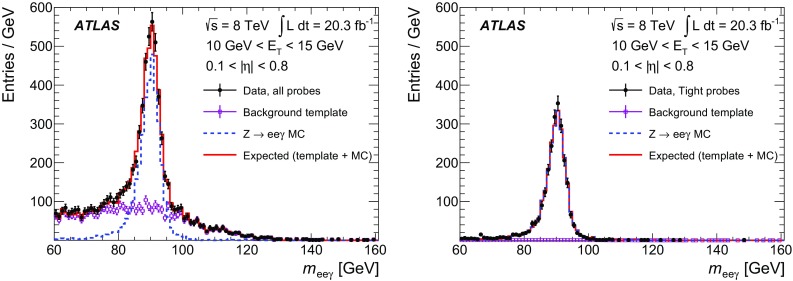
Illustration of the background estimation using the $Z \rightarrow ee\gamma$ method in the 10 $\text {GeV}$ < $E_{\mathrm {T}}$ < 15 $\text {GeV}$, 0.1 < $|\eta |$ < 0.8 bin, at reconstruction + track-quality level (*left*) and for probe electron candidates passing the cut-based Tight identification (*right*). The background template is normalized in the range 100 $\text {GeV}$ < $m_{ee}$ < 250 $\text {GeV}$. The tag electron passes cut-based Medium and isolation requirements. The signal MC simulation is scaled to match the estimated signal in the $Z$-mass window

Care is taken to remove the small contribution of signal electrons in the tails of the distribution of all probes before normalizing the background template to them. Tight probe electrons and Tight data efficiencies are used to perform this subtraction, except for the Tight efficiency extraction, for which the MC efficiency is used. For the numerator, the same templates are used as in the denominator, but they are normalized to the same-sign invariant-mass distribution (all numerator requirements are imposed on the probe). The normalization is done in the same ranges as in the denominator. The same-sign distribution is used as reference because it has less signal contamination than the opposite-sign distribution, an effect that is more important in the numerator. Figure 4 shows the $Z \rightarrow ee$ tag-and-probe invariant-mass distribution in one example bin for both numerator and denominator, including the normalized background template and the MC $Z \rightarrow ee$ prediction. Figure 5 shows the same for the $Z \rightarrow ee\gamma$ invariant-mass distribution.

In order to assess systematic uncertainties, efficiency measurements based on the following variations of the analysis are considered. The mass window is changed from 15 to 10 and 20 $\text {GeV}$ around the $Z$ mass, the tag electron requirement is varied by applying a requirement on the calorimetric isolation variable and, in the $Z \rightarrow ee$ case, by loosening the identification requirement to Medium. Furthermore, for $E_{\mathrm {T}}$ < 30 $\text {GeV}$, two normalization regions, below and above the $Z$ peak are used. The normalization range below the peak is 60 $\text {GeV}$ < $m_{ee}$ < 70 $\text {GeV}$. For $E_{\mathrm {T}}$ > 30 $\text {GeV}$, the number of events in the low-mass region is too small for a reliable normalization, so instead two different background template selections are considered. All possible combinations of these variations are produced and taken into account as described in Sect. 5.2.

#### Background estimation and variations for assessing the systematic uncertainties of the $Z_{\mathrm {iso}}$ method

In this approach, the calorimeter isolation distribution $E_\mathrm {T}^\mathrm {cone0.3}$ of the probe electron candidates is used as the discriminating variable.

**Fig. 6:**
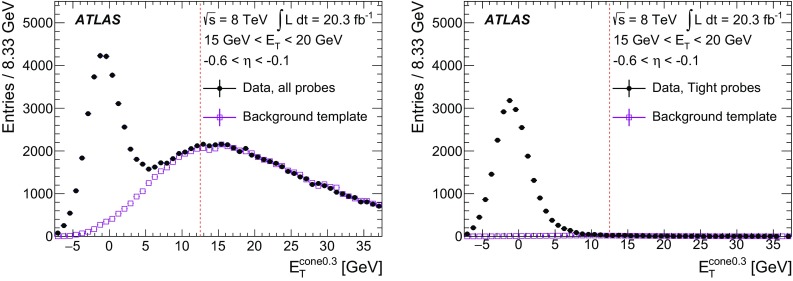
Illustration of the background estimation using the $Z_{\mathrm {iso}}$ method in the 15 $\text {GeV}$ < $E_{\mathrm {T}}$ < 20 $\text {GeV}$, −0.6 < $\eta$ < −0.1 bin, at reconstruction + track-quality level (*left*) and after applying the cut-based Tight identification (*right*). The tag electrons are selected using the cut-based Tight identification and a $Z$-mass window of 15 $\text {GeV}$ is applied. The threshold chosen for the sideband subtraction is $E_{\mathrm {T}}^\mathrm {cone0.3}$ $=$ 12.5 $\text {GeV}$

The background templates are formed as subsets of all probe electron candidates used in the denominator of the identification efficiency calculation. The probes for the background template are required to be reconstructed as electrons with a matching track that satisfies track quality criteria; however, they are required to fail some of the identification requirements, namely the requirements on ${ w}_\mathrm{stot}$ and $F_\text {HT}$. A study was performed on possible background templates and the bias due to the inversion of selection requirements and contamination from signal electrons. The least-biased templates were chosen. As illustrated in Fig. 6, the background templates are normalized to the isolation distribution of the probe electron candidates using the background dominated tail region of the isolation distribution.

To assess the systematic uncertainty of the efficiency, the parameters of the measurement are varied. The threshold for the sideband subtraction is chosen between $E_{\mathrm {T}}^\mathrm {cone0.3}$ $=$ 10 $\text {GeV}$ and $=$ 15 $\text {GeV}$. As in the $Z_{\mathrm {mass}}$ case, the mass window is changed from 15 $\text {GeV}$ to 10 $\text {GeV}$ and 20 $\text {GeV}$ around the $Z$ mass, the tag electron requirement is varied by applying a requirement on the calorimetric isolation variable, $E_\mathrm {T}^\mathrm {cone0.4}$ < 5 $\text {GeV}$.

In addition, different identification requirements are inverted to form two alternative templates and an alternative probe electron isolation distribution $E_\mathrm {T}^\mathrm {cone0.4}$ with a larger isolation cone size ($\Delta R = 0.4$) is used as the discriminant. As in the $Z_{\mathrm {mass}}$ case, all possible combinations of these variations are considered.

For the $Z_{\mathrm {mass}}$ and $Z_{\mathrm {iso}}$ methods together, there are in total 90 variations, which are treated as variations of the same measurement in order to estimate the systematic uncertainty due to the background estimation method.

### Tag-and-probe with $J/\psi \rightarrow ee$ events

$J/\psi \rightarrow ee$ events are used to measure the electron identification efficiency for 7 $\text {GeV}$ $< E_{\mathrm {T}}<$ 20 $\text {GeV}$. At such low energies, the probe sample suffers from a significant background fraction, which can be estimated using the reconstructed dielectron invariant mass ($m_{ee}$) of the selected tag-and-probe pairs. Furthermore, the $J/\psi$ sample is composed of two contributions. In prompt production, the $J/\psi$ meson is produced directly in the proton–proton collision via strong interaction or from the decays of directly produced heavier charmonium states. The electrons from the decay of prompt $J/\psi$ particles are expected to be isolated and therefore to have identification efficiencies close to those of isolated electrons from other physics processes of interest in the same transverse energy range, such as Higgs boson decays. In non-prompt production, the $J/\psi$ meson originates from *b*-hadron decays and its decay electrons are expected to be less isolated.

Experimentally, the two production modes can be distinguished by measuring the displacement of the $J/\psi \rightarrow ee$ vertex with respect to the primary vertex. Due to the long lifetime of *b*-hadrons, electron-pairs from non-prompt $J/\psi$ production have a measurably displaced vertex, while prompt decays occur at the primary vertex. To reduce the dependence on the $J/\psi$ transverse momentum, the variable used in this analysis to discriminate between prompt and non-prompt production, called pseudo-proper time [24], is defined as

3 $$\begin{aligned} \tau = \frac{ L_{xy} \cdot m^{J/\psi }_{\mathrm {PDG}} }{ p^{J/\psi }_\mathrm {T} }. \end{aligned}$$

Here, $L_{xy}$ measures the displacement of the $J/\psi$ vertex with respect to the primary vertex in the transverse plane, while $m^{J/\psi }_{\mathrm {PDG}}$ and $p^{J/\psi }_\mathrm {T}$ are the mass [25] and the reconstructed transverse momentum of the $J/\psi$ particle.

Two methods have been developed to measure the electron efficiency using $J/\psi \rightarrow ee$ decays. The short-$\tau$ method, already used in Refs. [2, 13], considers only events with short pseudo-proper time, selecting a subsample dominated by prompt $J/\psi$ production. The remaining non-prompt contamination is estimated using MC simulation and the measurement of the non-prompt fraction in $J/\psi \rightarrow \mu \mu$ events [26]. The $\tau$-fit method, used in Ref. [2], utilizes the full $\tau$-range and extracts the non-prompt fraction by fitting the pseudo-proper time distribution both before and after applying the identification requirements.

#### Event selection

Events are selected by five dedicated $J/\psi \rightarrow ee$ triggers. These require tight trigger electron identification^10^ and an electron $E_{\mathrm {T}}$ above a threshold for one of the two trigger objects, while only requiring an EM cluster above a certain $E_{\mathrm {T}}$ threshold for the other.

Events with at least two electron candidates with $E_{\mathrm {T}}>5$ $\text {GeV}$ and $|\eta |<2.47$ are considered.

The tag electron candidate must be matched to a tight trigger electron object within $\Delta R < 0.005$ and satisfy the cut-based Tight identification selection. To further clean the tag electron sample an isolation criterion is applied in most of the analysis variations. The other electron candidate, the probe, needs to satisfy the track quality criteria. It is also required to match an EM trigger object of the $J/\psi \rightarrow ee$ triggers within $\Delta R < 0.005$ and have a transverse energy that is at least 1 $\text {GeV}$ higher than the corresponding trigger threshold. To ensure that the measured efficiency corresponds to well-isolated electrons an isolation requirement is imposed on the probe electron candidate as well. The isolation criterion has less than 1% effect on the identification efficiency in simulated events. It is further required that the tag and probe electron candidates are separated by $\Delta R_\mathrm {tag-probe} > 0.2$ to prevent one electron from affecting the identification of the other. The pseudo-proper time of the reconstructed $J/\psi$ candidate is restricted to −1 ps $< \tau < 3$ ps in the $\tau$-fit method and typically to −1 ps $< \tau < 0.2$ ps in the short-$\tau$ method. The negative values of the pseudo-proper times are due to the finite resolution of $L_{xy}$. At this stage no requirement is made on the charge of the electrons and all possible tag-and-probe pairs are considered. About 700,000 probe electron candidates are selected for $E_{\mathrm {T}}=7$–20 $\text {GeV}$, of which about 190,000 pass the Tight selection, within the range of −1 ps $< \tau < 3$ ps and integrated over $|\eta |<2.47$.

#### Background estimation and variations for assessing the systematic uncertainties

**Fig. 7:**
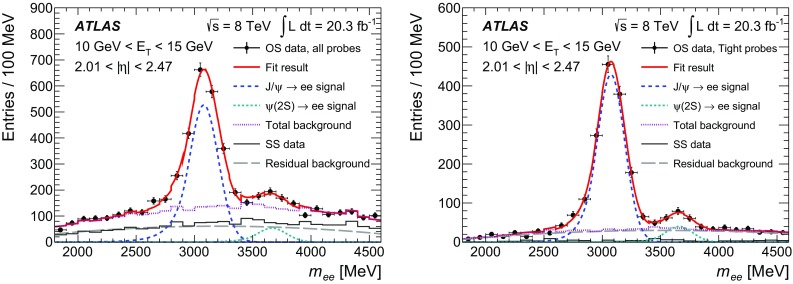
The figure demonstrates the background subtraction as carried out in the short-$\tau$ method. Shown is the dielectron invariant-mass fit for all probe electron candidates having a good track quality (*left*) and for probe electron candidates passing the cut-based Tight identification (*right*) for 10 $\text {GeV}$< $E_{\mathrm {T}}$ < 15 $\text {GeV}$ and 2.01 < $|\eta |<$ 2.47. A track isolation requirement of $p_\mathrm {T}^\mathrm {cone0.2}/E_\mathrm {T} < 0.15$ is placed on the probe electron candidate. The pseudo-proper time is required to be −1 ps $< \tau <0.2$ ps. *Dots with error bars* represent the opposite-sign (OS) pairs for data, the fitted $J/\psi$ signal is shown by the *dashed blue* and the $\psi$(2S) by the *dashed light blue lines* (both modelled by a Crystal-Ball function). A background fit is carried out using the sum of the same-sign (SS) distribution (*solid grey*) from data and a Chebyshev polynomial of 2nd order describing the residual background (*dashed grey*). The sum of the two background contributions is depicted as a *purple dotted line*

**Fig. 8:**
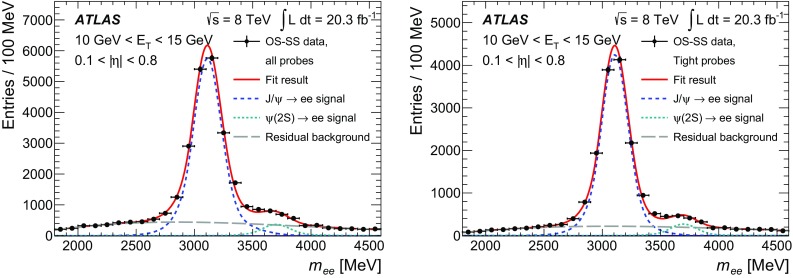
Illustration of the background determination for the $J/\psi$ analysis, in the $\tau$-fit method. The dielectron invariant-mass fit for all probe electron candidates passing track-quality requirements (*left*) and for probe electron candidates passing the cut-based Tight identification (*right*) for 10 $\text {GeV}$ $<E_{\mathrm {T}}<15$ $\text {GeV}$ and $0.1<\eta <0.8$ is shown. A track isolation requirement of $p_\mathrm {T}^\mathrm {cone0.2}/E_\mathrm {T} < 0.15$ is placed on both the tag and the probe electron candidates. The pseudo-proper time is required to be −1 ps < $\tau$ < 3 ps. *Dots with error bars* represent the OS minus SS data, the fitted $J/\psi$ signal is shown by the *dashed blue* and the $\psi$(2S) by the *dashed light blue lines* (both modelled by a Crystal-Ball + Gaussian function). The residual background (Chebyshev polynomial of 2nd order) is shown by the *dashed grey line*

The invariant mass of the tag-and-probe pair is used to discriminate between signal electrons and background. The most important contribution to the background, even after requiring the tag-and-probe pair to have opposite-sign (OS) charges, comes from random combinations of two particles. This can be evaluated – assuming charge symmetry – using the mass spectrum of same-sign (SS) charge pairs. The remaining background is small and can be described using an analytical model. For this, the invariant-mass distribution of the two electron candidates is fitted with the sum of three contributions: $J/\psi$, $\psi$(2S) and background, typically in the range of 1.8 $\text {GeV}$ < $m_{ee}$ < 4.6 $\text {GeV}$. To model the $J/\psi$ component, a Crystal-Ball [27, 28] function is used. In the $\tau$-fit method to better describe the tail, a Crystal-Ball + Gaussian function is used instead. The $\psi$(2S) is modelled with the same shape except for an offset corresponding to the mass difference between the $J/\psi$ and $\psi$(2S) states. Finally the residual background is modelled by a Chebyshev polynomial (as variation by an exponential function) with the parameters determined from a combined signal + background fit to the data. The background estimated using SS pairs is either added to the residual background in the binned fit (see Fig. 7 for the short-$\tau$ method) or subtracted explicitly before performing the unbinned fit (see Fig. 8 for the $\tau$-fit method). The number of $J/\psi$ candidates is counted within a mass window of 2.8 $\text {GeV}$ < $m_{ee}$ < 3.3 $\text {GeV}$.

**Fig. 9:**
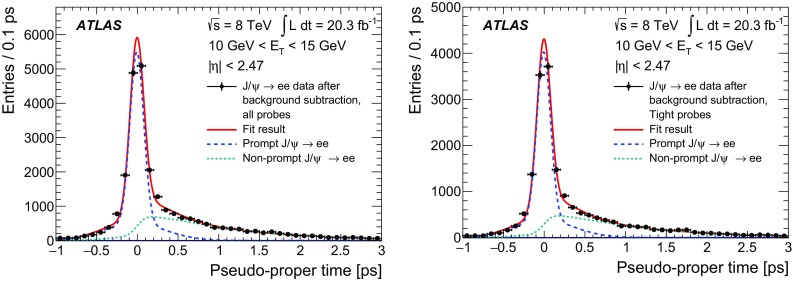
Pseudo-proper time fit for all probe electron candidates passing reconstruction + track-quality requirements (*left*) and for probe electron candidates passing the Tight identification (*right*) for 10 $\text {GeV}$ $<E_{\mathrm {T}}\ <15$ $\text {GeV}$, integrated over $|\eta |<2.47$. A calorimetric isolation requirement of $E_\mathrm {T}^\mathrm {cone0.2}/E_\mathrm {T} < 0.2$ is placed on the probe electron candidate. *Dots with error bars* represent the OS minus SS data with the residual background subtracted using the reconstructed dielectron mass distribution sidebands. The prompt signal component is shown by the *dashed blue line* (sum of two Gaussian functions) and the non-prompt signal component is shown by the *light blue dashed line* (exponential decay function convolved with the sum of two Gaussian functions)

In the $\tau$-fit method, the prompt component is then extracted by an unbinned fit of the pseudo-proper time distribution in the range −1 ps < $\tau$ < 3 ps, after correcting the contribution for the estimated background by subtracting the $\tau$ distribution in the mass sidebands 2.3 $\text {GeV}$ < $m_{ee}$ < 2.5 $\text {GeV}$ and 4.0 $\text {GeV}$ < $m_{ee}$ < 4.2 $\text {GeV}$ normalized to the estimated background within the signal mass window as given by the $m_{ee}$ fit. The non-prompt component is modelled by an exponential decay function convolved with the sum of two Gaussian functions, while the shape of the prompt component is described by the sum of the same Gaussian functions describing the detector resolution, as shown in Fig. 9.

In the short-$\tau$ method, strict requirements on $\tau$ are made, requiring it to be below 0.2 or 0.4 ps. The resulting non-prompt contamination is below $\sim$20%, decreasing with decreasing probe electron $E_{\mathrm {T}}$. The measured efficiency is compared to the prediction of the MC simulation after mixing the simulated prompt and non-prompt $J/\psi \rightarrow ee$ samples according to the ATLAS measurement of the non-prompt $J/\psi$ fraction in the dimuon final state at $\sqrt{s}=7$ $\text {TeV}$ [26].

Systematic uncertainties arise predominantly from the background estimation and the probe electron definition. They are estimated by varying the tag-and-probe selection (such as the isolation and the $\tau$ requirements), the fit parameters (background and signal shapes, fit window and sideband definitions) and the size of the mass window (changed by ±40%) for signal counting after the mass fit. In total, 186 variations were considered in each ($E_{\mathrm {T}}$, $|\eta |$) bin, using the two methods, to determine the efficiency and its uncertainty.

### Combination

To calculate the final results for the identification efficiency, the data-to-MC correction factors are combined. The following measurements are used in the different $E_{\mathrm {T}}$ bins:

- 7–10 $$\text {GeV}$$: $$J/\psi \rightarrow ee$$,
- 10–15 $$\text {GeV}$$: $$J/\psi \rightarrow ee$$ and $$Z \rightarrow ee\gamma $$,
- 15–20 $$\text {GeV}$$: $$J/\psi \rightarrow ee$$ and $$Z \rightarrow ee$$,
- 20–25 $\text {GeV}$ and bins above: $Z \rightarrow ee$.

Only the two $E_{\mathrm {T}}$ bins 10–15 and 15–20 $\text {GeV}$ allow a combination of independent measurements, which is done using a program originally developed for the HERA experiment [29] and used in Ref. [2]. It performs a $\chi ^2$ fit over all bins, separately for the bins below and above 20 $\text {GeV}$, adjusting the input values taking into account correlations of the systematic uncertainties in $\eta$ and $E_{\mathrm {T}}$ bins.

Both the $\chi ^2$ (ranging from 3.4 to 12.3 for 12 degrees of freedom, depending on the identification selection) and the pulls of the combination indicate good agreement for the measurements in the 10–15 and 15–20 $\text {GeV}$ bins.

### Results

The combined data efficiencies are derived by applying the combined data-to-MC efficiency ratios to the MC efficiency prediction from simulated $Z \rightarrow ee$ decays. Similarly, when comparing the results of different efficiency measurements, the measured data-to-MC efficiency ratios are used to correct the $Z \rightarrow ee$ MC sample.

The measured efficiencies for the various identification criteria are presented as functions of the electron $\eta$, $E_{\mathrm {T}}$ and the number of reconstructed primary collision vertices in the event. The latter is a measure of the amount of activity due to overlapping collisions which affects the reconstructed electrons, for example by making the calorimeter shower shapes more background-like due to nearby particles. The efficiency dependence in bins of primary vertices is only measured for electrons with $E_{\mathrm {T}}$ > 15 $\text {GeV}$ using $Z \rightarrow ee$ events with the $Z_{\mathrm {mass}}$ method, as the $J/\psi \rightarrow ee$ sample size is not large enough.

**Fig. 10:**
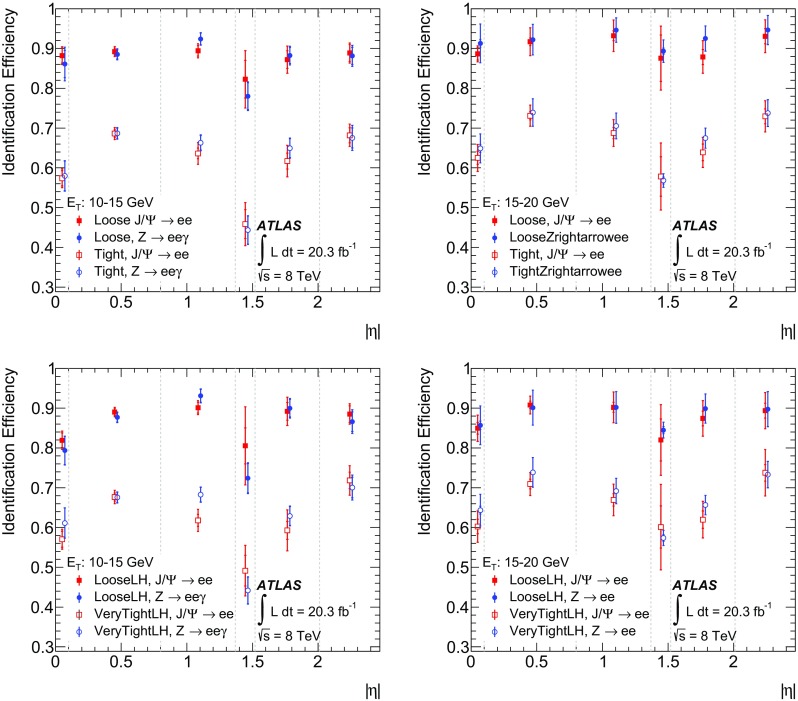
Measured identification efficiency as a function of $|\eta |$ for $E_{\mathrm {T}}=10$–15 $\text {GeV}$ (*left*) and $E_{\mathrm {T}}=15$–20 $\text {GeV}$ (*right*) for the cut-based Loose and Tight selections (*top*) and for Loose LH and Very Tight LH (*bottom*). The data efficiency is derived by applying the measured data-to-MC efficiency ratios, determined with either the $J/\psi$ or the $Z$ methods, to the prediction of the MC simulation from $Z \rightarrow ee$ decays. The uncertainties are statistical (*inner error bars*) and statistical + systematic (*outer error bars*). The *dashed lines* indicate the bins in which the efficiencies are calculated. For better visibility, the measurement points are displayed as slightly shifted with respect to each other

Figure 10 shows a comparison between efficiencies computed for $Z \rightarrow ee$ decays in the two $E_{\mathrm {T}}$ bins in which different measurements overlap. The methods agree well.

**Fig. 11:**
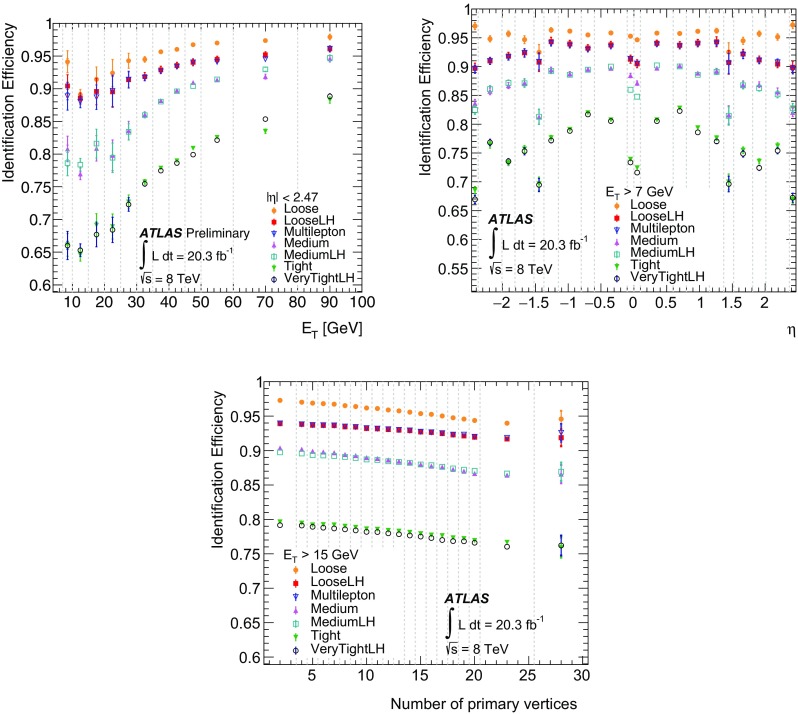
Measured identification efficiency for the various cut-based and LH selections as a function of $E_{\mathrm {T}}$ (*top left*), $\eta$ (*top right*) and the number of reconstructed primary vertices (*bottom*). The data efficiency is derived from the measured data-to-MC efficiency ratios and the prediction of the MC simulation from $Z \rightarrow ee$ decays. The uncertainties are statistical (*inner error bars*) and statistical + systematic (*outer error bars*). The last bin in $E_{\mathrm {T}}$ and number of primary vertices includes the overflow. The *dashed lines* indicate the bins in which the efficiencies are calculated

The efficiencies integrated over $E_{\mathrm {T}}$ or $\eta$, as well as the dependence on the number of primary vertices is shown in Fig. 11. These distributions assume the ($E_{\mathrm {T}}$, $\eta$) distribution of electrons from $Z \rightarrow ee$ decays and treat the total uncertainties as fully correlated between bins, as done for most analyses.

**Fig. 12:**
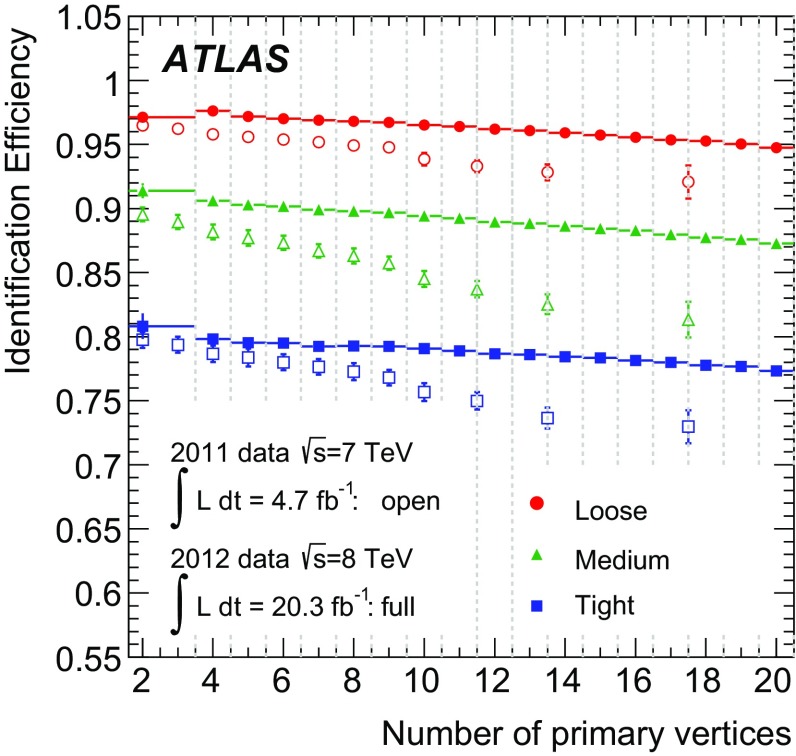
Identification efficiency for the various cut-based selections measured with 2011 and 2012 data as a function of the number of reconstructed primary vertices

With tighter requirements on more variables, the overall identification efficiency decreases, while the dependence on $E_{\mathrm {T}}$ and $\eta$ increases, as expected. The efficiency of the cut-based Multilepton selection shows less variation with the number of primary vertices than the cut-based Loose selection, as it relies less on the pile-up-sensitive variables $R_\eta$ and $R_\text {had}$. Overall, the 2012 update of the cut-based menu (see Sect. 4.1) has been successful: the efficiencies and rejections could be kept at values similar to those in 2011, while the remaining pile-up dependence is small (variation below 4% for 1 to 30 vertices). The improvement of the 2012 menu regarding the pile-up robustness of the requirements is demonstrated in Fig. 12, where the efficiencies for the cut-based Loose, Medium and Tight selections as a function of the number of reconstructed primary vertices are compared for 2011 and 2012.

The Loose LH is tuned to match the efficiencies of the cut-based Multilepton selection, while the (Medium LH) Very Tight LH is tuned to match those of the cut-based (Medium) Tight selection. The efficiency figures show that this tuning is successful in almost all bins. While the efficiencies match, the background rejection of the LH selections is better. The background efficiencies are reduced by a factor of about two when comparing the cut-based identification to the corresponding LH selections (see Sect. 8).

The efficiencies as a function of $E_{\mathrm {T}}$ and $\eta$, as presented in Fig. 11, show some well-understood features. The identification efficiencies in general rise as a function of $E_{\mathrm {T}}$ because electrons with higher $E_{\mathrm {T}}$ are better separated from the background in many of the discriminating variables. For the lowest (7–10 $\text {GeV}$) as well as for the highest (above 80 $\text {GeV}$) $E_{\mathrm {T}}$ bin, a significant and somewhat discontinuous increase in the identification efficiency is observed. This is explained by the fact that at very low and very high $E_{\mathrm {T}}$ some requirements are relaxed. For the high $E_{\mathrm {T}}$ bin the *E* / *p* requirement is removed, because the measurement of the electron’s track momentum is less precise for high-$p_{\mathrm {T}}$ tracks and can therefore not safely be used to distinguish electrons from backgrounds. It was checked that the data-to-MC correction factor measured for electrons above 80 $\text {GeV}$ is applicable to electrons even at $E_{\mathrm {T}}$ greater than 400 $\text {GeV}$ using the $Z_{\mathrm {iso}}$ method. Within the large statistical uncertainties, data-to-MC correction factors binned in $E_{\mathrm {T}}$ for the high-$E_{\mathrm {T}}$ region were found to agree with the combined data-to-MC correction factor above 80 $\text {GeV}$ that is presented in this paper. The lowest $E_{\mathrm {T}}$ bin (7–10 $\text {GeV}$) was tuned separately from the other bins, choosing the signal efficiency to be a few percentage points higher. This leads to higher background contamination.

The shape of the identification efficiency distributions as a function of $\eta$ is mainly due to features of the detector design and the selection optimization procedure that is typically based on the signal-to-background ratio. A small gap between the two calorimeter half-barrels and in the TRT around $|\eta | \approx 0$ explains the slight drop in efficiency. Another, larger drop in efficiency is observed for $1.37<|\eta |<1.52$, where the transition region between the barrel and endcap calorimeters is situated. At high $|\eta |$ the efficiencies are lower due to the larger amount of material in front of the endcap calorimeters.

**Fig. 13:**
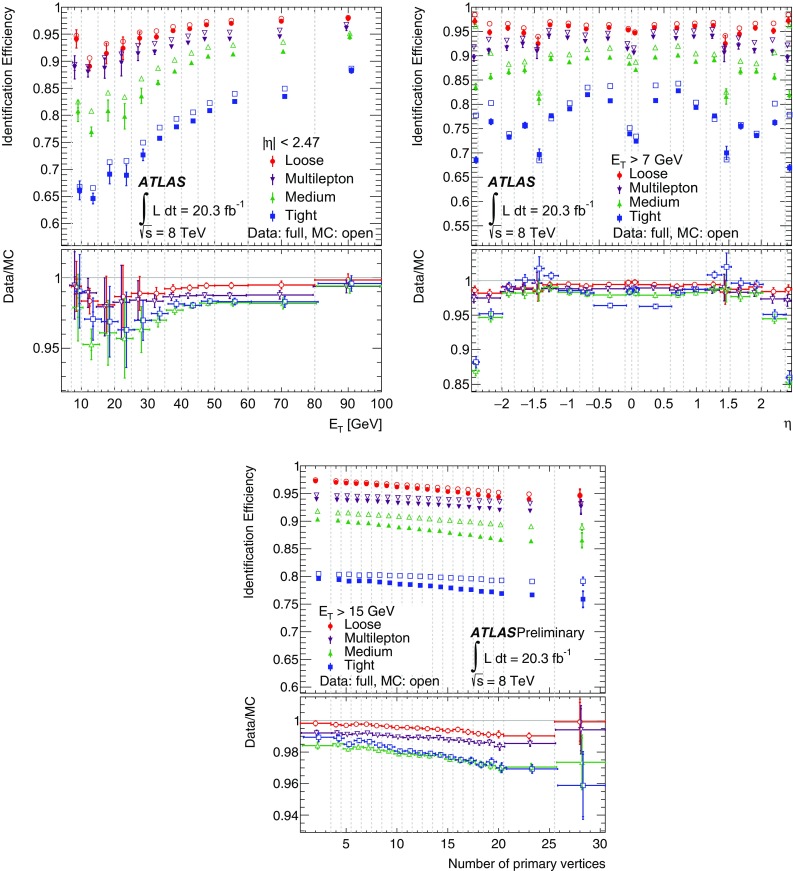
Identification efficiency in data as a function of $E_{\mathrm {T}}$ (*top left*), $\eta$ (*top right*) and the number of reconstructed primary vertices (*bottom*) for the cut-based Loose, Multilepton, Medium and Tight selections, compared to predictions of the MC simulation for electrons from $Z \rightarrow ee$ decay. The *lower panel* shows the data-to-MC efficiency ratios. The data efficiency is derived from the measured data-to-MC efficiency ratios and the prediction of the MC simulation for electrons from $Z \rightarrow ee$ decays. The last bin in $E_{\mathrm {T}}$ and number of primary vertices includes the overflow. The uncertainties are statistical (*inner error bars*) and statistical + systematic (*outer error bars*). The *dashed lines* indicate the bins in which the efficiencies are calculated

**Fig. 14:**
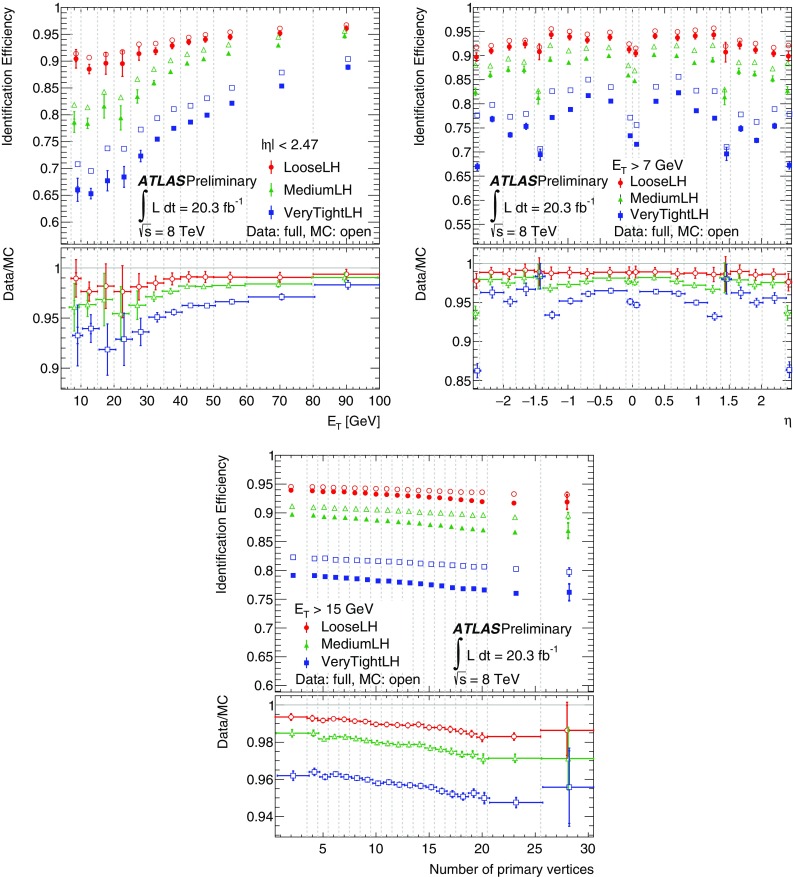
Identification efficiency in data as a function of $E_{\mathrm {T}}$ (*top left*), $\eta$ (*top right*) and the number of reconstructed primary vertices (*bottom*) for Loose LH, Medium LH and Very Tight LH selections, compared to predictions of the MC simulation for electrons from $Z \rightarrow ee$ decay. The *lower panel* shows the data-to-MC efficiency ratios. The data efficiency is derived from the measured data-to-MC efficiency ratios and the prediction of the MC simulation for electrons from $Z \rightarrow ee$ decays. The last bin in $E_{\mathrm {T}}$ and number of primary vertices includes the overflow. The uncertainties are statistical (*inner error bars*) and statistical + systematic (*outer error bars*). The *dashed lines* indicate the bins in which the efficiencies are calculated

Figures 13 and 14 show the identification efficiencies when integrated over $E_{\mathrm {T}}$ or $\eta$, and as a function of the number of reconstructed primary vertices. These figures depict in their lower panels the data-to-MC correction factors. As can be seen, the correction factors are close to one, with cut-based selections showing better data–MC agreement than the LH. Only for low $E_{\mathrm {T}}$ or high values of $\eta$, corrections reaching 10% have to be applied for the more stringent selection criteria. The combined statistical and systematic uncertainties in the data-to-MC correction factors range from 0.5 to 10%, with the highest uncertainties found at low $E_{\mathrm {T}}$, and in the transition region of the calorimeter, 1.37 $<|\eta |<$ 1.52. At low $E_{\mathrm {T}}$, a large contribution to the uncertainties is statistical in nature and can be considered uncorrelated between bins when propagating the uncertainties to the final results of analyses (in the presented figures the uncertainties are treated as fully correlated between bins).

As discussed in Ref. [13], the difference between identification efficiencies in data and MC simulation can be traced back to differences in the distribution of the variables used in the identification, particularly the shower shape variables and the TRT high-threshold hit ratio $F_\text {HT}$, the latter being defined only for $|\eta |<2$. The distributions of the lateral shower shapes are not well modelled by the GEANT4-based simulation of the detector: in comparison to predictions of the MC simulation, most shower shapes in data are wider and centred at values closer to the background distributions. These effects lead to higher efficiencies in MC simulation. $F_\text {HT}$, on the other hand, is underestimated in the simulation for $|\eta |$ > 1, leading to higher efficiencies in data than in the simulation. These two effects cancel each other, as can be seen in Fig. 13, where the data and MC efficiency values of the cut-based Tight selection are quite close to each other for 1 $<|\eta |<$ 2.

Figures 13 and 14 show that the data has a more significant dependence on pile-up than predicted by simulation. For the cut-based Multilepton and Loose selections, the data-to-MC ratio is almost constant as a function of the number of primary vertices, while it decreases for the cut-based Medium and Tight selections as well as the LH selections by about 2% from 1 to 30 primary vertices. This effect is primarily caused by the mismodelling in MC simulation of the $R_\mathrm {Had(1)}$, ${ w}_\mathrm{stot}$ and $F_\text {HT}$ variables. The $F_\text {HT}$ variable is sensitive to the pile-up conditions due to higher occupancies in events with many vertices, which can lead to hit overlaps in the TRT straws increasing the chance of passing the high threshold. The effect is not well modelled by the simulation, independent of the modelling of the pile-up itself. Both the $R_\mathrm {Had(1)}$ and ${ w}_\mathrm{stot}$ variables, as well as additional energy deposits from pile-up particles, are not well modelled by the GEANT4 simulation of the calorimeter, leading to differences as a function of pile-up between data and MC simulation. The pile-up profile of the collision data analyses which use the results of these efficiency measurements is very close to the pile-up profile of the efficiency measurements presented here. The data-to-MC correction factors will therefore adjust the MC efficiencies in the collision data analyses for the residual pile-up dependence.

In general, the mismodelling of the distributions affects cut-based and LH selections differently. For cut-based selections, a mismodelling in MC simulation is reflected in the efficiency only if it occurs around the cut value. In the case of the LH, a mismodelling anywhere in the distribution can affect the efficiency. The harder the requirement on the discriminant of the LH, the larger the effect of the differences between data and MC distributions on the data-to-MC correction factors, as can be seen in Figs. 13 and 14.

## Identification efficiency for background processes

The three main categories of electron background (in descending order of abundance after electron reconstruction) are light-flavour hadrons, electrons from conversions and Dalitz decays (referred to as background electrons in the following), and non-isolated electrons from heavy-flavour decays. The background efficiencies of the different identification selections were studied using both MC simulation and data.

### Background efficiency from Monte Carlo simulation

The efficiencies of the different identification selections for backgrounds were studied using MC simulation of all relevant $2\rightarrow 2$ QCD processes filtered at particle level to mimic a level-1 EM trigger requirement. The sample is enriched in electron backgrounds, with electrons from *W* and $Z$ decays excluded at particle level using generator-level simulation information. Furthermore, the sample is required to pass a set of electron and photon triggers without identification criteria, to allow better comparison with data-driven measurements. The estimated background efficiency and the composition of the background are shown in Table 3 for reconstructed electron candidates passing track quality requirements with transverse energies between 20 and 50 $\text {GeV}$. The quoted uncertainties are statistical only. The composition of this background-enriched sample is categorized according to simulation information: non-isolated electrons from heavy-flavour decays, electrons from conversions and Dalitz decays, and hadrons. No explicit isolation requirement is applied. In analyses of collision data, the background efficiencies translate to background from multijet processes of typically 2–10% for leptonic *W* and semileptonic $t\bar{t}$ decays, where the cut-based Tight identification and some moderate isolation requirements have been applied. For a typical selection for a *Z* cross-section measurement that relies on the cut-based Medium identification, the multijet background is below 0.5% in the *Z* mass peak region.

**Table 3:** Background efficiency of different identification selections taken from a MC simulation containing all relevant $2\rightarrow 2$ QCD processes. The reconstructed electron candidates are required to have transverse energies between 20 and 50 $\text {GeV}$ and electrons from *W* and $Z$ decays are removed at particle level. Furthermore, the sample is required to pass a set of electron and photon triggers without identification criteria, to allow better comparison with data-driven measurements. The composition of the sample is categorized according to MC simulation information: non-isolated electrons from heavy-flavour decays, background electrons from photon conversions and Dalitz decays, and hadrons. The background efficiency for each category is also quoted. The efficiency is always quoted with respect to reconstructed electron candidates passing the track quality requirement. For completeness, the isolated electron efficiency for $Z\rightarrow ee$ decays, measured from data, is also given. The uncertainties are statistical only

Selection	Data efficiency (%)	MC efficiency (%)	Background composition (%)			MC efficiency (%) for background categories		
	$Z\rightarrow ee$ signal (prompt iso *e*)	Background (prompt *e* excluded)	Non-iso *e*	bkg *e*	Hadron	Non-iso *e*	bkg *e*	Hadron
20 $<E_{\mathrm {T}}<50$ $\text {GeV}$								
Track quality	100	100	1.1	16.1	82.8	100	100	100
Loose requirements	95.7 ± 0.2	4.76 ± 0.04	7.4	48.4	44.2	32.5±0.8	14.3±0.2	2.54±0.03
Multilepton requirements	92.9 ± 0.2	1.64 ± 0.02	22.5	34.5	43.0	34.2±0.8	3.51±0.08	0.85±0.02
Medium requirements	88.1 ± 0.2	1.11 ± 0.02	25.8	50.5	23.7	26.5±0.8	3.46±0.08	0.32±0.01
Tight requirements	77.5 ± 0.2	0.46 ± 0.01	54.5	29.9	15.6	23.0±0.7	0.85±0.04	0.086±0.006
Loose LH	92.8 ± 0.2	0.94 ± 0.02	40.2	42.0	17.9	34.8±0.8	2.44±0.07	0.20±0.01
Medium LH	87.8 ± 0.3	0.51 ± 0.01	48.8	40.6	10.7	23.1±0.7	1.29±0.05	0.066±0.005
Very Tight LH	77.0 ± 0.3	0.29 ± 0.01	63.7	28.9	7.4	16.9±0.7	0.51±0.03	0.026±0.003

After applying the looser cut-based selections, the background generally consists of hadrons and background electrons in similar fractions, with a small contribution of electrons from heavy-flavour decays. As the cut-based selections get tighter, heavy-flavour decays begin to dominate the remaining background, followed by background electrons. In contrast, the Loose LH selection retains significantly less hadronic background than its cut-based counterpart; instead, non-isolated and background electrons dominate in this regime. After the Very Tight LH selection, hadrons are highly suppressed and the sample is dominated by non-isolated electrons. To suppress these further, in many analyses isolation and tighter impact parameter requirements are added to the electron identification selection.

To estimate absolute background efficiencies, it is necessary to determine the efficiency for background objects to pass the denominator requirement of the relative efficiencies listed in Table 3. An unfiltered MC sample consisting of minimum-bias, single- and double-diffractive events is used. The numerator consists of reconstructed electron candidates passing the trigger and track quality requirements with transverse electron energy $E_{\mathrm {T}}>20$ $\text {GeV}$. The denominator is defined as the numerator plus any object reconstructed as a hadronic jet using the anti-$k_t$ jet reconstruction algorithm [30], with a radius parameter $R =$ 0.4, and transverse jet energy $E_{\mathrm {T},\mathrm {jet}}>20$ $\text {GeV}$. Jets overlapping with reconstructed electron candidates within a $\Delta R$ of 0.4 are removed to prevent double-counting. Reconstructed objects matched to simulated electrons from *W* and *Z* decays are also removed from the calculation. Using this methodology, it is found that $8.89\%\pm 0.16\%$ (stat.) of the simulated jets built from hadrons, photon conversions or heavy-flavour decays are reconstructed as electrons with $E_{\mathrm {T}}$ > 20 $\text {GeV}$ and pass trigger and track quality requirements. The efficiencies in Table 3 can be multiplied by this number to obtain absolute background efficiencies for jets with $E_{\mathrm {T}}$ > 20 $\text {GeV}$.

### Background efficiency ratios measured from collision data

Studying the electron backgrounds in MC simulation can give an approximate estimate of the background efficiency. However, the description of the MC simulation has several limitations: misidentification efficiencies depend on the tails of the distributions of many discriminating variables, which are typically more susceptible to mismodelling than the core of the distribution. Furthermore, a small deviation in shape can lead to a large data-to-MC efficiency correction factor due to the low fraction of candidates in the tails. A data-driven estimate of the background efficiency is therefore essential. In this section, the ratio of background efficiencies from cut-based and LH menus is determined using data.

An inclusive background sample is selected by a set of electron and photon triggers with different $E_{\mathrm {T}}$ thresholds and no identification requirement. To prevent contamination from isolated electrons from *W* and $Z$ decays, the reconstructed electron candidate (matched to the trigger electron) is rejected if it forms a pair with an invariant mass of 40–140 $\text {GeV}$ with an electron candidate passing the Medium requirement. Likewise, the electron candidate is also rejected if there is significant missing transverse momentum in the event ($E_{\mathrm {T}}^{\mathrm {miss}}> 25$ $\text {GeV}$ 11), or if the transverse mass calculated using $E_{\mathrm {T}}^{\mathrm {miss}}$ is compatible with *W*-boson production ($m_{\mathrm {T}}>40$ $\text {GeV}$). In order to remove the residual true electron contamination, these kinematic requirements are furthermore applied to simulated $Z \rightarrow ee$ and $W \rightarrow e\nu$ samples; the surviving events are scaled to the corresponding integrated luminosity and subtracted from the data yields before the background efficiency calculation.

**Fig. 15:**
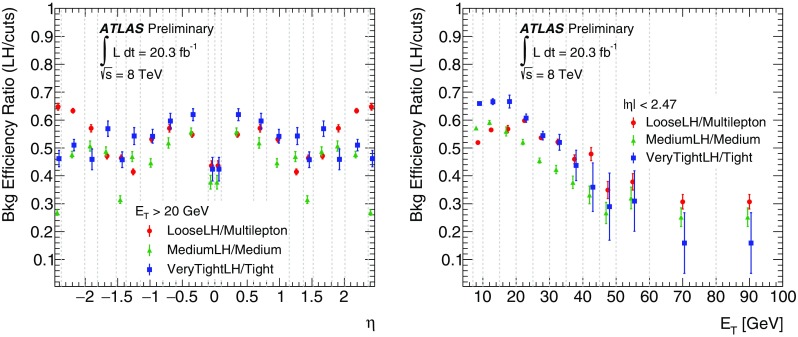
Ratio of background efficiencies for a LH to that of the closest-efficiency cut-based selections as a function of $\eta$ (*left*) and $E_{\mathrm {T}}$ (*right*), as obtained using an inclusive background sample (see text). The uncertainties are statistical as well as systematic: a systematic uncertainty of 21% is assigned to the subtraction of signal events using the simulation; this uncertainty is dominated by the mismodelling of the missing transverse momentum

The background sample is dominated by light-flavour hadrons, followed by photon conversions and a small fraction of heavy-flavour decays. The ratio of the background efficiency for a LH to that for the closest-efficiency cut-based selection is shown in Fig. 15. It can be seen that the LH selections let through only about 40–60% of the background compared to the cut-based selections, while it is shown in Sect. 7.4 that they retain approximately the same signal electron efficiency. These results cannot be directly compared to those derived from MC simulation and given in Table 3, as the composition of the samples might differ. Nonetheless, the data-driven and MC-based estimates show the same trend when comparing the background rejection of cut-based and LH selections.

## Determination of the charge misidentification probability

Charge misidentification occurs if an isolated prompt electron is reconstructed with a wrong charge assignment. The misidentification is mostly caused by the emission of bremsstrahlung at a small angle with a subsequent conversion of the emitted photon and the mismatching of one of the conversion tracks to the cluster of the original electron. In addition, for high $E_{\mathrm {T}}$ and therefore increasingly straight tracks, charge misidentification can be caused by a failure to correctly determine the curvature of the track matched to the electron. For electrons with transverse energies of $E_{\mathrm {T}}<300$ $\text {GeV}$, the causes of charge misidentification are predominantly conversions combined with inefficiencies in matching the correct track to the electron.

Various physics analyses such as measurements of same-sign *WW* scattering [31] or *Z* polarization [32] as well as searches for supersymmetry in final states with two same-sign leptons [33] rely on correct charge assignment. Therefore the measurement of the charge misidentification rate and its description in MC simulation is crucial.

In the range of $E_{\mathrm {T}}$ for which the *Z* decays yield a sufficiently large sample, and which is used by most analyses, the charge misidentification probability is dominated by material effects, rather than the precision of the measurement of the track curvature, as studies using MC simulation have shown. Therefore the charge misidentification rate is determined as a function of $\eta$ rather than $E_{\mathrm {T}}$ using electrons with $E_{\mathrm {T}}$ greater than 15 $\text {GeV}$.

**Fig. 16:**
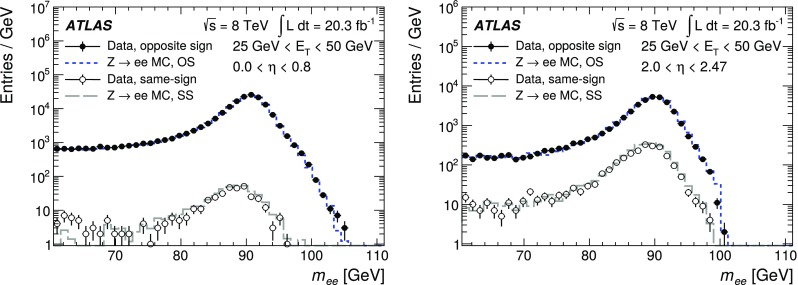
Distribution of the invariant mass $m_{ee}$ of the selected opposite-sign (OS) or same-sign (SS) electron pairs in data and MC simulation for 25 $\text {GeV}$ < $E_{\mathrm {T}}$ < 50 $\text {GeV}$ in the 0.0 < $\eta$ < 0.8 bin (*left*) and in the 2.0 < $\eta$ < 2.47 bin (*right*). Tag and probe electron candidates are required to pass the cut-based Tight identification and a track isolation requirement of $p_\mathrm {T}^\mathrm {cone0.2}/E_\mathrm {T} < 0.14$

The event selection described in Sect. 7.1.1 is applied to select a sample of $Z \rightarrow ee$ events, except for the opposite-charge requirement. Additionally, both the tag and probe electron candidates are required to satisfy certain identification and isolation criteria. Figure 16 shows the $m_{ee}$ distribution of the selected OS and SS electron pairs for a representative selection.

The probability for an electron to be charge misidentified in a certain bin *i* in $\eta$ and $E_{\mathrm {T}}$ is referred to as $\epsilon _i$. The probabilities $\epsilon _i$ in the different regions are statistically independent. The average number of SS events $N^{\mathrm {SS}}_{ij}$ that is expected for a pair of electrons in the bins *i* and *j* follows from the number of total events $N^{\mathrm {OS+SS}}_{ij}$, where no charge requirement is applied, using the respective charge misidentification probabilities $\epsilon _{i,j}$ as:

4 $$\begin{aligned} N^{\mathrm {SS}}_{ij} = N^{\mathrm {OS+SS}}_{ij}[(1-\epsilon _i)\epsilon _j + (1-\epsilon _j)\epsilon _i]. \end{aligned}$$

$N^{\mathrm {SS+OS}}_{ij}$ is taken from data after background subtraction. A likelihood function can be constructed using a Poissonian approximation of the probability to observe a specific number of SS events $n^{\mathrm {SS,obs}}_{ij}$ in data if the electrons are reconstructed in the bins *i* and *j*:

5 $$\begin{aligned} L = \prod _{i,j}L_{ij} =\prod _{i,j} \frac{(N^{\mathrm {SS}}_{ij} + N^{\mathrm {SS, bkg}}_{ij})^{n^\mathrm {SS,obs}_{ij}}\times e^{N^{\mathrm {SS}}_{ij}+ N^{\mathrm {SS, bkg}}_{ij}}}{ n^\mathrm {SS,obs}_{ij}}! \end{aligned}$$

The likelihood function is maximized to estimate the charge misidentification probabilities $\epsilon _i$ in each bin *i*.

As in the other efficiency measurements, the backgrounds originate from hadronic jets as well as from photon conversions, Dalitz decays and semileptonic heavy-flavour hadron decays. The backgrounds for total and same-sign candidate events are estimated by extrapolating linearly the number of events from equally sized sidebands of the invariant-mass distributions above and below the *Z* mass peak to the signal region. As an estimate of the uncertainties, the measurement is performed by varying the invariant-mass window from 15 to 10 and 20 $\text {GeV}$ around the $Z$ mass, the width of the sidebands used in the background subtraction is changed to be 20, 25, or 30 $\text {GeV}$. All variations have very small effects on the measured rates. The average value of these variations is taken as the measured value, the RMS as the systematic uncertainty. The uncertainty returned by the minimization is accounted for as a statistical uncertainty.

The charge misidentification rate is determined for three representative sets of requirements applied in analyses:

- **Medium** Medium identification requirements.
- **Tight + isolation** Tight identification requirements combined with selection criteria for the track isolation of $p_\mathrm {T}^\mathrm {cone0.2}/E_\mathrm {T} < 0.14$.
- **Tight + isolation + impact parameter** Tight identification combined with calorimetric and track isolation criteria of $E_\mathrm {T}^\mathrm {cone0.3}/E_\mathrm {T} < 0.14$ and $p_\mathrm {T}^\mathrm {cone0.2}/E_\mathrm {T} < 0.07$ and in addition requirements on the track impact parameters of $|z_0| \times \sin \theta <0.5$ mm and $|d_0|/\sigma _{d_0}<$ 5.0.

**Fig. 17:**
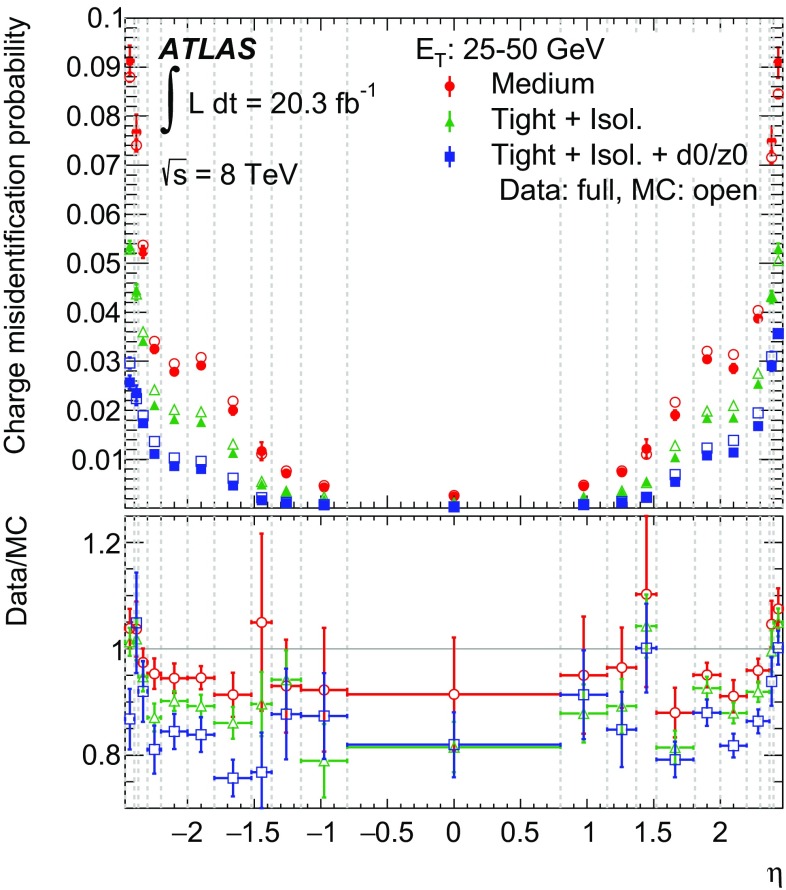
Charge misidentification probability in data as a function of $\eta$ for three different sets of selection requirements (Medium, Tight + Isolation and Tight + Isolation + impact parameter), compared to the expectation of the MC simulation as measured on a sample of electron pairs from $Z \rightarrow ee$ decays. The *lower panel* shows the data-to-MC charge misidentification probability ratios. The uncertainties are the total uncertainties from the sum in quadrature of statistical and systematic uncertainties. The *dashed lines* indicate the bins in which the efficiencies are calculated

Figure 17 shows the charge misidentification probability for the three working points as determined by the measurement in data and MC simulation. Since the charge misidentification probability is correlated with the amount of bremsstrahlung and thus with the amount of traversed material, the probabilities are quite low in the central region of the detector but can reach almost 10% for high values of $|\eta |$. The energy in a cone around the electron can be indicative of energy deposited by bremsstrahlung. Equally, large values of the track impact parameters can mean that the track matched to the electron is not a prompt track from the primary vertex but from a secondary interaction or bremsstrahlung and a subsequent conversion. Thus, tighter selection criteria, in particular requirements on the isolation or track parameters, can decrease the charge misidentification probability by a factor of up to four, depending on the additional selection requirements.

## Reconstruction efficiency measurement

### Tag-and-probe with $Z \rightarrow ee$ events

Electrons are reconstructed from EM clusters that are matched to tracks in the ID, as described in Sect. 3. The tracks are required to satisfy the track quality criteria, i.e. to have at least one hit in the pixel detector and in total at least seven hits in the pixel and SCT detectors. The measurement of the efficiency to detect an energy cluster in the EM calorimeter using the sliding-window algorithm is very challenging in data and not performed here. In MC simulation, it is found to be above 99% for $E_{\mathrm {T}}$ > 15 $\text {GeV}$ as discussed in Sect. 3. EM clusters form the starting point of the reconstruction efficiency measurement.

The reconstruction efficiency is defined as the ratio of the number of electrons reconstructed as a cluster matched to a track satisfying the track quality criteria (numerator) to the number of clusters with or without a matching track (denominator). This reconstruction efficiency is measured using a tag-and-probe analysis which is very similar to the $Z_{\mathrm {mass}}$ method introduced in Sect. 7. In comparison to the measurement of the identification efficiency, the probe definition is relaxed to include all EM clusters. The background estimation is adapted to include the contribution of EM clusters with no associated track. The measurement is only performed for probe electron candidates with $E_{\mathrm {T}}$ > 15 $\text {GeV}$, as the background contamination of the sample becomes too high at lower $E_{\mathrm {T}}$.

#### Event selection

The general event selection as well as the criteria for the tag electron are identical to the ones used in the $Z_{\mathrm {mass}}$ method, described in Sect. 7.1.1.

Each event is required to have at least one tag electron candidate and one probe, which in this case is an EM cluster. In order to veto EM clusters from converted photons, no other cluster within $\Delta R$ $=$ 0.4 of a reconstructed electron candidate is considered. No requirement on the charge of the tag and the probe electron candidates is applied, since there is no charge associated with EM clusters unless they are matched to a track.

#### Background estimation and variations for assessing the systematic uncertainties

The background estimation for the numerator of the reconstruction efficiency (electrons passing the reconstruction requirements) follows that of the $Z_{\mathrm {mass}}$ method described in Sect. 7.1.2. However, for the denominator (all reconstructed EM clusters) an additional contribution from photon candidates must be determined separately. The total background at the denominator level is the sum of two contributions: background to electrons reconstructed as a cluster with and without an associated track. The background estimation for these two contributions is explained below.

**Fig. 18:**
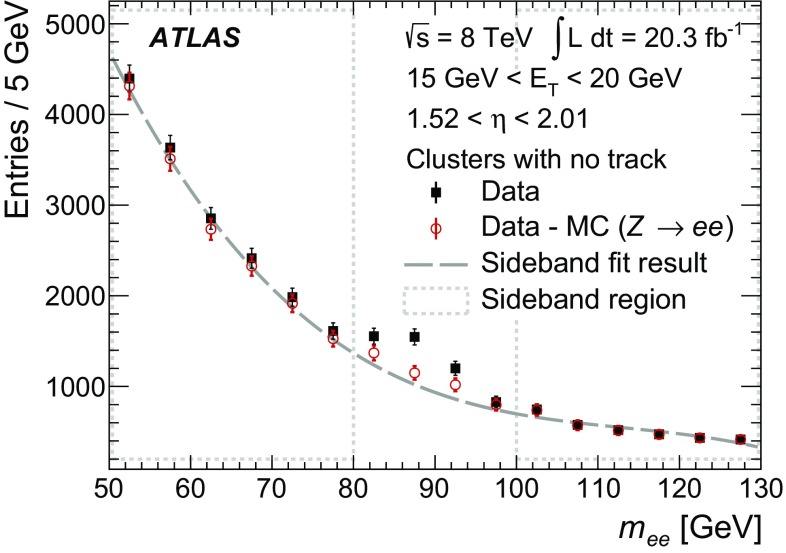
Estimate of the background to the selected EM clusters with no associated track for 15 $\text {GeV}$ < $E_{\mathrm {T}}$ < 20 $\text {GeV}$ and 1.52 < $\eta$ < 2.01. A polynomial fit (shown by a *dashed dark grey line*) is carried out in the sideband region (indicated by *dashed light grey boxes*) of the invariant-mass distribution of data events from which genuine electrons have been subtracted using MC simulation (the data are shown by *filled squares* before the subtraction of the prediction of the MC simulation and by *open circles* afterwards). In the signal region, defined as the events with an invariant mass of 80 $\text {GeV}$ to 100 $\text {GeV}$, the fit result is used to obtain a data-driven estimate, which is compared to the data minus the prediction of the MC simulation. Only statistical uncertainties are shown for the data minus the MC prediction; the systematic uncertainty in the scaling of the MC simulation and description of the MC simulation of the inefficiency to match an electron with a track is 10–20%

**Background estimate for electrons reconstructed as clusters with no associated track** Electrons reconstructed as EM clusters but not matched to any track are interpreted as photons. In order to estimate the photon background, which, unlike the signal electrons, has a smoothly falling invariant-mass shape, a third-order polynomial is fitted to the invariant-mass distribution of the selected electron–photon pairs (corresponding to the tag and the probe electron candidates). The fit is carried out using the two sideband regions above and below the $Z$ mass peak, as illustrated in Fig. 18. Residual signal electron contamination in the background-dominated sideband regions is subtracted using MC simulation before the fit. Systematic uncertainties in the scaling of the MC simulation and description of the MC simulation of the inefficiency to match an electron with a track are 10–20% and are not shown in Fig. 18. These uncertainties explain the small difference in the signal region between the data minus the MC prediction and the polynomial fit to the sidebands. The prediction of the MC simulation enters only in the subtraction of the very small residual signal in the sideband regions used to perform the polynomial fit. The resulting uncertainty in the measured reconstruction efficiency is negligible.

**Background estimate for electrons reconstructed as clusters with an associated track** The method to estimate the background to EM clusters with an associated track is almost the same as for the identification efficiency measurement, described in Sect. 7.1.2: A background template is selected in data by inverting identification selection criteria for the probes and normalized to the data in a control region of the invariant-mass distribution of the tag-and-probe pair.

The backgrounds in the signal region are determined separately for clusters with tracks satisfying or not satisfying the track quality selection criteria. Therefore, the track quality selection criteria must be satisfied (not satisfied) in the background template selection for the invariant-mass distribution of EM clusters passing (failing) the electron reconstruction procedure.

**Fig. 19:**
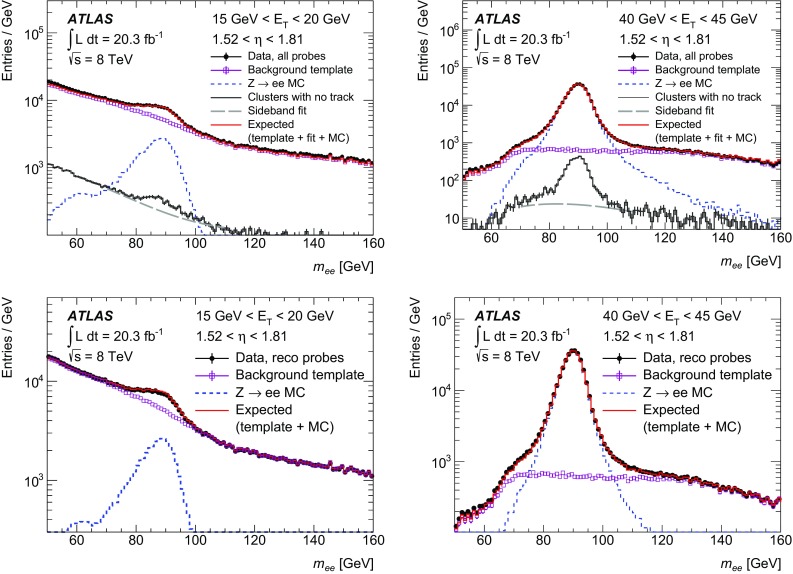
Invariant-mass distributions of the tag-and-probe pairs for probe EM clusters with 1.52 < $\eta$ < 1.81 and 15 $\text {GeV}$ $< E_{\mathrm {T}}< 20$ $\text {GeV}$ (*left*) or 40 $\text {GeV}$ $< E_{\mathrm {T}}< 45$ $\text {GeV}$ (*right*), before (*top*) and after (*bottom*) applying the reconstruction criteria. The data (*black dots with error bars*) at the *all probes* level is composed of two components: clusters with no matching track (*dark grey histogram with error bars*) and clusters with a matching track. The background is evaluated separately for these two components. A third-order polynomial (*grey dashed line* spanning the region from 70 $\text {GeV}$ to 110 $\text {GeV}$) depicts the estimated photon background from a fit performed in the sideband regions as explained in Sect. 10.1.2 and shown in Fig. 18. A background template normalized in this case to the high-mass tail (*magenta markers*) is used to estimate the background with a matching track. This background template is obtained by requiring some of the identification criteria not to be satisfied. Additionally, probes must pass or fail the track quality selection requirements depending on whether the background to the electrons passing or failing the reconstruction requirements is determined (see Sect. 10.1.2). The shown magenta distribution is the sum of both components. For illustration only, the signal prediction of the MC simulation (*blue dashed line*) is also displayed. The sum of the normalized background template and the signal prediction of the MC simulation (*red line*, shown for comparison but not used in the measurement) agrees well with the data points

Figure 19 shows the invariant-mass distributions of the tag-and-probe pairs for probe EM clusters (composed of clusters with or without a track match at the probe level) for two selected bins both at the probe level and before and after applying the reconstruction criteria to the probe electron candidate. The estimates of the two background components are also depicted. As demonstrated by the figure, the measured data agree well with the prediction, and the background subtraction procedure performs well.

The systematic uncertainty is estimated as for the identification efficiency. In addition to the variations listed in Sect. 7.1.2, the sidebands for the polynomial fit used for the estimation of the background to electrons without an associated track are varied among these choices: $[70,80\, \text {GeV}]$ and $[100,110\, \text {GeV}]$, $[60,80\, \text {GeV}]$ and $[100,120\, \text {GeV}]$, $[50,80 \,\text {GeV}]$ and $[100,130\, \text {GeV}]$, $[55,70\, \text {GeV}]$ and $[110,125\, \text {GeV}]$.

### Results

**Fig. 20:**
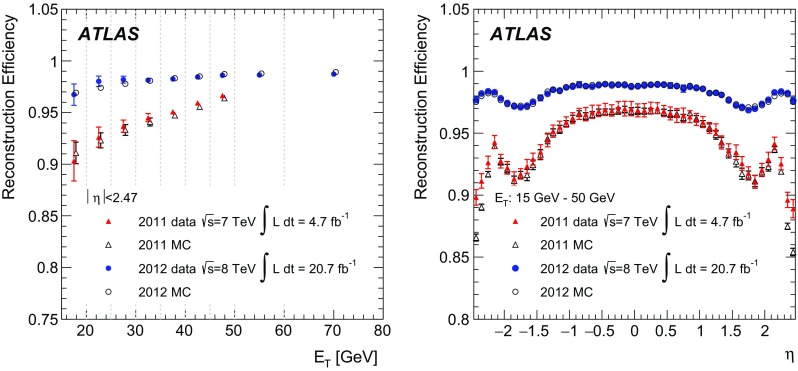
Measured reconstruction efficiencies as a function of $E_{\mathrm {T}}$ integrated over the full pseudorapidity range (*left*) and as a function of $\eta$ for 15 $\text {GeV}$ < $E_{\mathrm {T}}$ < 50 $\text {GeV}$ (*right*) for the 2011 (*triangles*) and the 2012 (*circles*) data sets. For illustration purposes a finer $\eta$ binning is used. The *dashed lines in the left plot* indicate the bins in which the efficiencies are calculated

The reconstruction efficiency, like the identification efficiency, is measured differentially in ($E_{\mathrm {T}}$, $\eta$) bins. The efficiency to reconstruct an electron associated with a track of good quality varies from 95% to 99% between the endcap and barrel regions for low-$E_{\mathrm {T}}$ electrons ($E_{\mathrm {T}}$ < 20 $\text {GeV}$). For very high $E_{\mathrm {T}}$ electrons ($E_{\mathrm {T}}$ > 80 $\text {GeV}$) the efficiency is $\sim$99% over the whole $\eta$ range. The results are shown in Fig. 20, projected in $E_{\mathrm {T}}$ and $\eta$. The measured efficiency agrees well with the prediction of the MC simulation. The data-to-MC correction factors are at most 1–2% different from unity and in most of the measurements they are within only a few permille of one. The total uncertainty is < 0.5% for electrons with $E_{\mathrm {T}}$ between 25 and 80 $\text {GeV}$. It is larger at lower $E_{\mathrm {T}}$, varying between 0.5 and 2.0%. The statistical and systematic uncertainties are of the same order. Good data–MC agreement observed for $E_{\mathrm {T}}> 15$ $\text {GeV}$ gives confidence in the description of the MC simulation of the detector response, which is relied on for electrons with $E_{\mathrm {T}}< 15$ $\text {GeV}$. In this low-$E_{\mathrm {T}}$ region, the data-to-MC correction factor is assumed to be 1.0 with an uncertainty of 2% in the barrel and 5% in the endcap region.

**Fig. 21:**
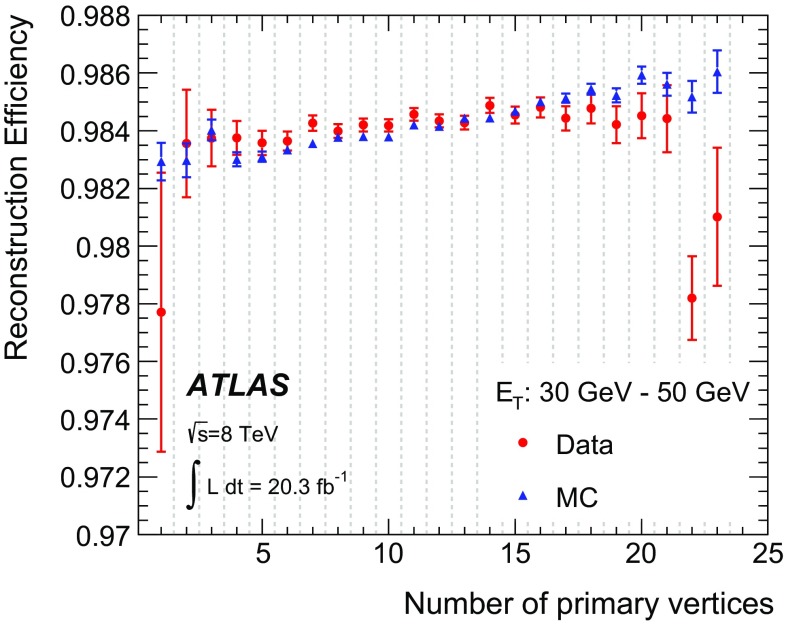
Measured reconstruction efficiency (*red circles*) as a function of the number of reconstructed primary vertices for 30 $\text {GeV}$ < $E_{\mathrm {T}}$ < 50 $\text {GeV}$ and integrated over $\eta$, compared to the prediction of the MC simulation (*blue triangles*). The uncertainties are statistical + systematic. The *dashed lines* indicate the bins in which the efficiencies are calculated

As described in Sect. 3, for the 2012 data, a new track reconstruction algorithm has been introduced in order to improve the reconstruction of electrons that have undergone significant bremsstrahlung. Figure 20 also compares the reconstruction efficiencies measured in the 2011 and 2012 data. The new track fitting algorithm improves the overall electron reconstruction efficiency by $\sim$5%. Most of this improvement is in the low-$E_{\mathrm {T}}$ range, where the electron reconstruction efficiency increases by more than $\sim$7%. This constitutes a significant gain for important measurements such as the determination of Higgs boson properties in the channel $H \rightarrow ZZ^* \rightarrow 4\ell$ [34].

The gain in efficiency from the new track reconstruction algorithm flattens the distribution of the reconstruction efficiency in $\eta$. For the 2011 data, a large drop in efficiency was observed for the endcap regions, where more bremsstrahlung occurs due to a higher amount of material. For the 2012 data, this drop has become much smaller. Furthermore, the 2012 results are more precise than the final 2011 results, partly because of the increase in the size of the available data sample, but also due to improvements in the background subtraction method.

The efficiencies are also measured as a function of the number of primary vertices in order to investigate the dependence of the electron reconstruction on pile-up. Figure 21 shows that for data, the reconstruction efficiency for electrons with $E_{\mathrm {T}}$ > 30 $\text {GeV}$ does not change with the number of primary vertices.

## Combined reconstruction and identification efficiencies

**Fig. 22:**
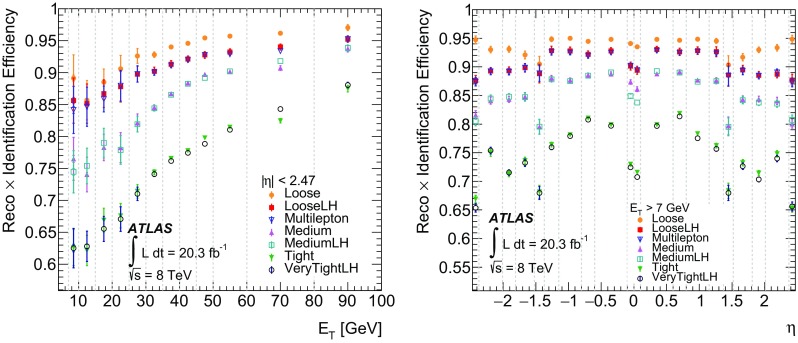
Measured combined reconstruction and identification efficiency for the various cut-based and LH selections as a function of $E_{\mathrm {T}}$ (*left*) and $\eta$ (*right*) for electrons. The data efficiency is derived from the measured data-to-MC efficiency ratios and the prediction of the MC simulation from $Z \rightarrow ee$ decays. The uncertainties are statistical (*inner error bars*) and statistical + systematic (*outer error bars*). The last $E_{\mathrm {T}}$ bin includes the overflow

**Fig. 23:**
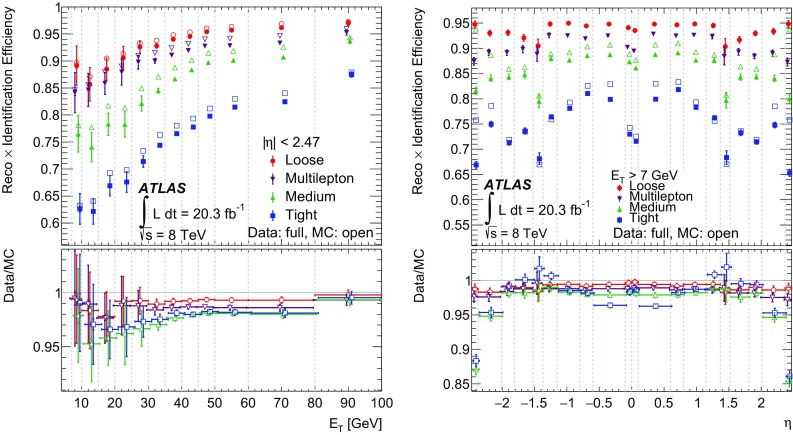
Measured combined reconstruction and identification efficiency as a function of $E_{\mathrm {T}}$ (*left*) and $\eta$ (*right*) for the cut-based Loose, Multilepton, Medium and Tight selections, compared to expectation of the MC simulation for electrons from $Z \rightarrow ee$ decay. The lower panel shows the data-to-MC efficiency ratios. The data efficiency is derived from the measured data-to-MC efficiency ratios and the prediction of the MC simulation for electrons from $Z \rightarrow ee$ decays. The uncertainties are statistical (*inner error bars*) and statistical + systematic (*outer error bars*). The last $E_{\mathrm {T}}$ bin includes the overflow

**Fig. 24:**
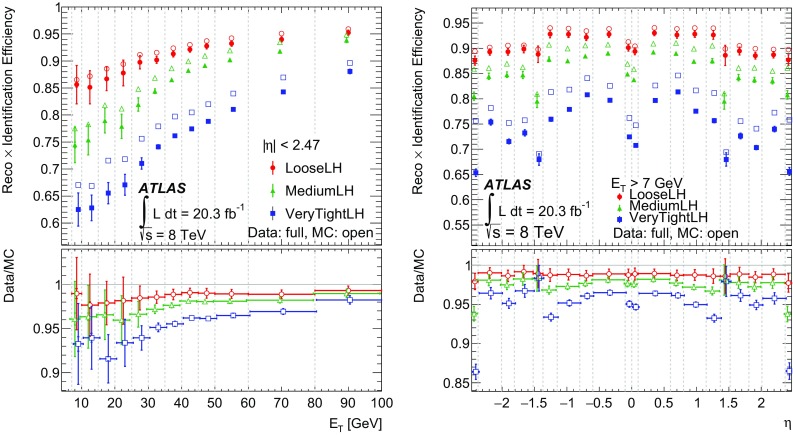
Measured combined reconstruction and identification efficiency as a function of $E_{\mathrm {T}}$ (*left*) and $\eta$ (*right*) for the Loose LH, Medium LH and Very Tight LH selections, compared to predictions of the MC simulation for electrons from $Z \rightarrow ee$ decay. The lower panel shows the data-to-MC efficiency ratios. The data efficiency is derived from the measured data-to-MC efficiency ratios and the prediction of the MC simulation for electrons from $Z \rightarrow ee$ decays. The uncertainties are statistical (*inner error bars*) and statistical + systematic (*outer error bars*). The last $E_{\mathrm {T}}$ bin includes the overflow

Figure 22 shows the combined efficiencies to reconstruct and identify electrons with respect to reconstructed energy clusters in the EM calorimeter for all identification selections. The efficiencies are shown as a function of $E_{\mathrm {T}}$ and $\eta$. As described in Sect. 7.4, the measured data-to-MC correction factors are applied to a simulated $Z \rightarrow ee$ sample. The resulting efficiencies correspond to the measured data efficiencies and can be compared to the efficiencies of simulated electrons in $Z \rightarrow ee$ events as done in Figs. 23 and  24. For electrons with $E_{\mathrm {T}}< 15$ $\text {GeV}$, the reconstruction efficiency cannot be measured and is taken instead from the MC simulation.

The combined efficiency to reconstruct and identify an electron from $Z \rightarrow ee$ with $E_{\mathrm {T}}$ around 25 $\text {GeV}$ is about 92% for the Loose cut-based identification and around 68% for the Tight cut-based identification as well as the Very Tight LH selection. It is lower (higher) at lower (higher) $E_{\mathrm {T}}$, with a sharper turn-on as well as a greater $\eta$ dependence for the tighter selections. Since the reconstruction efficiency is constant, the shapes are mainly determined by the variation of the identification efficiency (see Sects. 7, 10).

## Summary

Using the full 2012 data set, 20.3 $\mathrm{fb}^{-1}$ of 8 $\text {TeV}$ *pp* collisions produced by the LHC, the reconstruction, identification, and charge misidentification efficiencies of central electrons in the ATLAS detector are determined using a tag-and-probe method. Reconstruction and charge misidentification efficiencies are measured for electrons from $Z \rightarrow ee$ decays. The identification efficiency measurements from $J/\psi$ and $Z$ decays are combined using data-to-MC efficiency ratios, improving the precision of the results.

In 2012, a new track reconstruction algorithm and improved track-cluster matching were introduced to recover efficiency losses due to electrons undergoing bremsstrahlung. As a result, the overall electron reconstruction efficiency is increased by roughly 5% with respect to the 2011 efficiency. Averaged over $\eta$, it is about 97% for electrons with $E_{\mathrm {T}}=15$ $\text {GeV}$ and reaches about 99% at $E_{\mathrm {T}}= 50$ $\text {GeV}$. For electrons with $E_{\mathrm {T}}$ > 15 $\text {GeV}$, the reconstruction efficiency varies from 99% at low $|\eta |$ to 95% at high $|\eta |$.

The uncertainty on the reconstruction efficiency is below 0.5% for $E_{\mathrm {T}}$ > 25 $\text {GeV}$, and between 0.5–2% at lower transverse energy. Below 15 $\text {GeV}$, the reconstruction efficiency is not measured due to the overwhelming background contamination of the sample.

The electron identification was improved by loosening the selection criteria for the shower shapes in the EM calorimeter that are most affected by the increased instantaneous luminosities provided by the LHC in 2012. To compensate for the loss in rejection power, new selection criteria were introduced and requirements on variables less sensitive to pile-up were tightened. Additionally, new identification selections were developed: the cut-based Multilepton selection, optimized for low-energy electrons, as well as an identification based on the likelihood (LH) approach. Using the LH identification selections, the background rejection is significantly improved while maintaining the same signal efficiency as that of the cut-based selections. The identification efficiency has a strong dependence on $E_{\mathrm {T}}$ and, for the tighter criteria, on $\eta$. Calculated with respect to reconstructed electrons satisfying quality criteria for their tracks, it averages between 96% (cut-based Loose) and 78% (Very Tight LH) for electrons with $E_{\mathrm {T}}$ > 15 $\text {GeV}$. The measured pile-up dependence is below 4% for 1–30 reconstructed primary collision vertices per bunch crossing for all sets of selection criteria. Some differences between the behaviour in data and MC simulation are observed, but understood. The total uncertainties in the identification efficiency measurements are 5–6% (1–2%) for electrons below (above) $E_{\mathrm {T}}$ = 25 $\text {GeV}$.

Charge misidentification of electrons in the probed $E_{\mathrm {T}}$ range is mostly caused by the emission of bremsstrahlung. The charge misidentification depends strongly on the applied selection criteria as well as on the $\eta$ of the electron. For representative selections the probability is at sub-percent level for $|\eta | < 1$ and can be as high as 10% for $|\eta | \sim$ 2.5.

The measured data-to-MC efficiency ratios are applied as correction factors in analyses, such as the measurement of the properties of the Higgs boson, and their uncertainties are propagated accordingly. The scale factors are close to unity with deviations larger than a couple of percent from unity occurring only for low-$E_{\mathrm {T}}$ or high-$|\eta |$ regions.

## Footnotes

1 ATLAS uses a right-handed coordinate system with its origin at the nominal *pp* interaction point at the centre of the detector. The positive *x*-axis is defined by the direction from the interaction point to the centre of the LHC ring, with the positive *y*-axis pointing upwards, while the beam direction defines the *z*-axis. The azimuthal angle $\phi$ is measured around the beam axis and the polar angle $\theta$ is the angle from the *z*-axis. The pseudorapidity is defined as $\eta = -\ln \tan (\theta /2)$. The radial distance between two objects is defined as $\Delta R = \sqrt{{(\Delta \eta )}^2+{(\Delta \phi )}^2}$. Transverse energy is computed as $E_{\mathrm {T}}= E \cdot \sin \theta$.

2 As in the 2011 electron reconstruction algorithm, clusters must satisfy loose requirements on the maximum fraction of energy deposited in the different layers of the EM calorimeter system: 0.9, 0.8, 0.98, 0.8 for the presampler detector, the strip, the middle and the back EM accordion calorimeter layers, respectively.

3 The transverse momentum threshold for tracks reconstructed with the pion hypothesis is 400 $\text {MeV}$ based on the pattern recognition.

4 Throughout the paper, when counting hits in the pixel and SCT detectors, non-operational modules that are traversed by the track are counted as hits.

5 Unconverted (converted) photon clusters, which are used in the reconstruction efficiency measurement in Sect. 10, are built using $3 \times 5$ ($3 \times 7$) cells in the barrel and $5 \times 5$ ($5 \times 5$) cells in the endcap.

6 The shower depth is defined as $X = \Sigma _i X_iE_i/ \Sigma _i E_i$ where $E_i$ is the cluster energy in layer *i* and $X_i$ is the approximate calorimeter thickness (in radiation lengths) from the interaction point to the middle of layer *i*, including the presampler detector layer where present.

7 Here an “event” refers to a triggered bunch crossing with all its hard and soft *pp* interactions, as recorded by the detector.

8 Another selection, Tight LH, was originally also developed with the background rejection matching the background rejection of the Tight cut-based selection, but it was never used. Therefore, for the LHC Run 2, Very Tight LH was renamed to Tight LH.

9 The electron identification selection in the trigger is looser than or equivalent to the corresponding analysis requirements.

10 The tight electron identification selection applied in the $J/\psi$ trigger is looser than the corresponding analysis requirements. In particular, no selection is applied to $\Delta \phi$, *E* / *p* and isConv.

11 The $E_{\mathrm {T}}^{\mathrm {miss}}$ is the magnitude of the negative vectorial sum of the transverse momenta from calibrated objects, such as identified electrons, muons, photons, hadronic decays of tau leptons, and jets. Clusters of calorimeter cells not associated with any object are also included.
